# Supplementary material for: Impact of hepatopathy in pediatric patients after surgery for complex congenital heart disease
Source: PLoS One. 2021 Mar 25;16(3):e0248776. doi: 10.1371/journal.pone.0248776 (PMC7993827; doi:10.1371/journal.pone.0248776)
Supplement: S1 Material — (ZIP) [file pone.0248776.s001.zip › CardiacHepatopathy_analysis_R.pdf]

Auswertung für das „HerzLeber“ - Projekt

Gerhard Schön[a]

Version 2.15 vom 3. Februar 2021

Zusammenfassung

Dieses Papier enhält die Auswertung für das „HerzLeber“ - Projekt. Die Aufbereitung der Daten ist in der Datei `HerzLeberDatenaufbereitung.Vers.1.6.pdf` dokumentiert. Alle Berechnungen und Plots wurden mit dem Statistikprogramm **R** Version 4.0.3[1] durchgeführt.

Version history

Version	Datum	Änderungen
2.15	03.02.2021	Anpassung für die Dokumentation zur Primärpublikation.
2.14	29.12.2020	Korrektur der Modelle.
2.13	29.12.2020	2 multivariate Modelle hinzugefügt.
2.12	28.12.2020	Für die bivariate Darstellung die Variablen „`lh_dysfct`“ (vgl. Abschnitt 1.50 auf Seite 63) und „`hypotens`“ (vgl. Abschnitt 4.42 auf Seite 85) eingefügt. Mean, sd, Test für zusätzliche Laborwerte (ab Abschnitt 4.41). Kaplan-Meier-Analyse für INR (vgl. Abschnitt 3.10 auf Seite 76) und Kreatinin (vgl. Abschnitt 3.11 auf Seite 77 eingefügt.
2.11	28.09.2020	Abbildung 68 auf Seite 73 und Abbildung 69 auf Seite 74 aktualisiert.
2.10	26.09.2020	Abbildung 65 auf Seite 70 aktualisiert.
2.9	25.09.2020	Abbildung 65 auf Seite 70 aktualisiert. Variablen „`syndr_assc`“ (vgl. Abschnitt 4.3 auf Seite 78) und „`proc_rel_fact`“ (vgl. Abschnitt 4.37 auf Seite 84) korrigiert.

[a] Universitätsklinikum Hamburg-Eppendorf, Institut für Medizinische Biometrie und Epidemiologie, Kontakt: g.schoen@uke.de

2.8	24.09.2020	Abbildungen 63 auf Seite 68 bis Abbildungen 70 auf Seite 75 aktualisiert. Variable „pro_rel_fact“ dichotomisiert dargestellt; vgl. Abschnitt 4.37 auf Seite 84. Variable „death“ (vgl. Abschnitt 4.26 auf Seite 82), Variable „syndr_assc“ (vgl. Abschnitt 4.3 auf Seite 78) und Variable „sex“ (vgl. Abschnitt 4.2 auf Seite 78) dargestellt. Die bivariaten logistischen Regressionen für die logarithmierten Variablen „transf_ekz“ (vgl. 1.18 auf Seite 23), „transf_ffp“ (vgl. 1.20 auf Seite 25) und „transf_tkz“ (vgl. 1.22 auf Seite 27) durchgeführt.
2.7	28.08.2020	Korrektur der Datenbasis. Abbildung 62 auf Seite 67 aktualisiert.
2.6	24.07.2020	2 weitere multivariate Modelle hinzugefügt.
2.5	24.06.2020	Deskription und *p*-Werte für Table 1 hinzugefügt; vgl. Abschnitt 4 auf Seite 78.
2.4	24.06.2020	Logistische Regressionen hinzugefügt; vgl. Abschnitt 1.41 auf Seite 46 bis Abschnitt 4.23 auf Seite 82.
2.3	17.01.2020	Weitere Multivariate Modelle hinzugefügt. Zeitachse bei Abbildung 62 auf Seite 67 angepasst.
2.2	24.09.2019	Kaplan-Meier Auswertungen hinzugefügt; vgl. Abschnitt 3 auf Seite 67.
2.1	23.09.2019	Auswertung nur für die Zeilen der Index-OP.
2.0	26.08.2019	Neue Analyse mit neuer Datenlieferung vom 26.07.2019.
1.1	12.12.2018	Cluster-Effekt „Person“ berücksichtigt. Fehlende unabängige Variablen dargestellt.
1.0	19.09.2018	Erste Darstellung.

Inhaltsverzeichnis

1 Bivariate Darstellung 4
1.1 Sex 4
1.2 Age 5
1.3 cat_chd 6
1.4 sts_eacts 7
1.5 unsched_reopb 8
1.6 hc_pre_op 9
1.7 time_cpb [h] 10
1.8 time_act [h] 11
1.9 time_reperf [h] 12
1.10 ecmo 13
1.10.1 ecmo Zeit für alle ecmo=yes 14
1.11 secTC 15
1.12 rh.dysfct 16
1.13 vent_duration 17
1.14 vent_before_op 18
1.15 dialy 19
1.15.1 dialy Zeit für alle dialy=yes 20
1.16 hosp_time 21
1.17 transf_ekz 22
1.18 transf_ekz, logarithmiert 23
1.19 transf_ffp 24
1.20 transf_ffp, logarithmiert 25
1.21 transf_tkz 26
1.22 transf_tkz, logarithmiert 27
1.23 mix.zirk 28
1.24 zvs_min 29
1.25 lakt_max 30
1.26 lak_norm 31
1.27 infect 32
1.28 bnp.min 33
1.29 bnp.max 34
1.30 tnt.min 35
1.31 tnt.max 36
1.32 gldh.min 37
1.33 gldh.max 38
1.34 quick.min 39
1.35 alb.min 40
1.36 bili.max 41
1.37 got.max 42
1.38 gpt.max 43
1.39 ggt.max 44

1.40 proc.rel.fact 45
1.41 proc.rel.fact.ple 46
1.42 proc.rel.fact.cyt 47
1.43 proc.rel.fact.diap 49
1.44 proc.rel.fact.throm 51
1.45 proc.rel.fact.hrs 53
1.46 use.nor 55
1.47 use.supra 57
1.48 use.milr 59
1.49 tpn 61
1.50 lh.dysfct 63
1.51 hypotens 64

2 Multivariates Modell 65

3 Time to event 67
3.1 BNP 67
3.2 TNT 68
3.3 GLDH 69
3.4 Prothrombin time 70
3.5 ALB 71
3.6 BILI 72
3.7 GOT 73
3.8 GPT 74
3.9 GGT 75
3.10 INR 76
3.11 KREA 77

4 p-Werte für table 1 78
4.1 age 78
4.2 sex 78
4.3 syndr.assc 78
4.4 sts.eacts 78
4.5 time.cpb 79
4.6 time.act 79
4.7 ecmo 79
4.8 dialy 79
4.9 secTC 79
4.10 transf.ekz 79
4.11 transf.ffp 80
4.12 transf.tkz 80
4.13 rh.dysfct 80
4.14 zvs.min 80
4.15 lakt.max 80

4.16 proc.rel.fact.ple 80
4.17 proc.rel.fact.cyt 81
4.18 proc.rel.fact.diap 81
4.19 proc.rel.fact.throm 81
4.20 proc.rel.fact.hrs 81
4.21 unsched.reop 81
4.22 infect 81
4.23 tpn 82
4.24 vent.duration 82
4.25 hosp.time 82
4.26 death 82
4.27 bnp.max 82
4.28 tnt.max 82
4.29 gldh.max 83
4.30 quick.min 83
4.31 alb.min 83
4.32 bili.max 83
4.33 got.max 83
4.34 gpt.max 83
4.35 ggt.max 83
4.36 proc.rel.fact 84
4.37 proc.rel.fact2 84
4.38 use.nor 84
4.39 use.supra 84
4.40 use.milr 84
4.41 lh.dysfct 84
4.42 hypotens 85
4.43 inr.prop 85
4.44 inr.max 85
4.45 krea.prop 85
4.46 krea.postop 85
4.47 bnp.min 85
4.48 tnt.min 85
4.49 gldh.min 86
4.50 alb.prop 86
4.51 bili.prop 86
4.52 got.prop 86
4.53 gpt.prop 86
4.54 ggt.prop 86

Abbildungsverzeichnis **87**

1 Bivariate Darstellung

Die Person mit der ID = 7 wird aus den Daten entfernt.

1.1 Sex

```
Häufigkeitstabelle: sex
   0  1     0     1
0 23  7 76.67 23.33
1  7 13 35.00 65.00
```

```
Logistische Regression: sex
              OR 2.5 % 97.5 % p-value
(Intercept) 0.30  0.12   0.67   0.006
sex         6.10  1.82  22.67   0.005
```

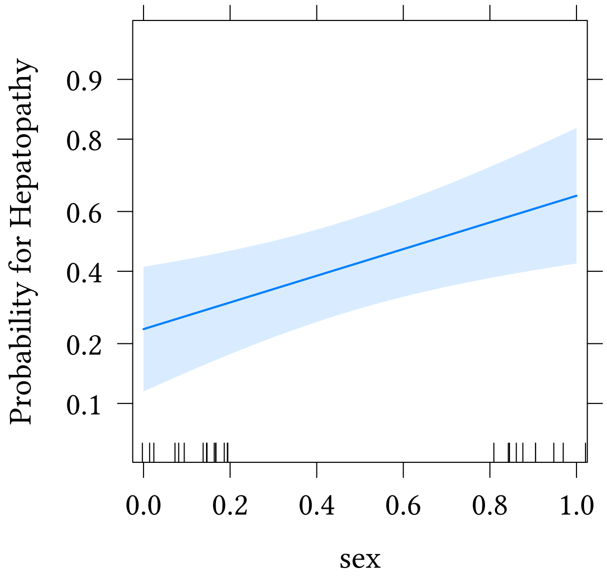

Abbildung 1: Wahrscheinlichkeit für Hepatopathy
(sex.pdf, sex.png)

1.2 Age

```
Summary: age
$`0`
  n mean   sd median  25%  75%  min  max range
 30 0.32 0.32    0.2 0.04 0.51 0.01 1.11   1.1

$`1`
  n mean   sd median  25%  75%  min  max range
 20 0.67 0.79   0.42 0.14 0.87 0.01 2.74  2.74
```

```
Logistische Regression: age
              OR 2.5 % 97.5 % p-value
(Intercept) 0.38  0.16   0.82   0.019
age         3.42  1.12  15.50   0.060
```

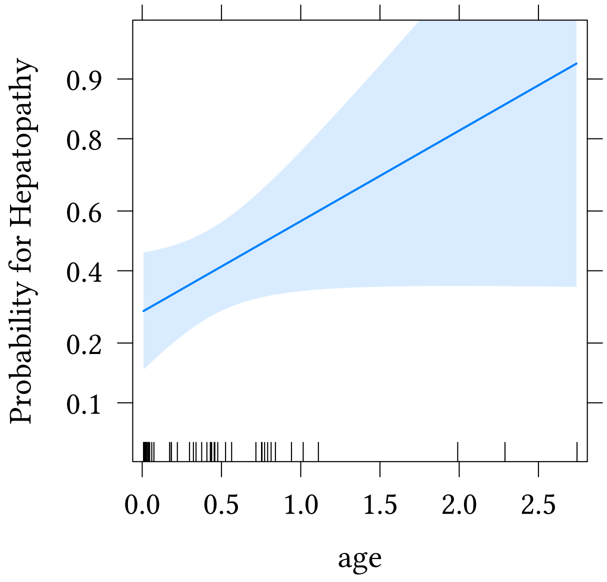

Abbildung 2: Wahrscheinlichkeit für Hepatopathy
(age.pdf, age.png)

1.3 cat_chd

```
Häufigkeitstabelle: cat.chd
      0 1      0      1
I     3 4  42.86  57.14
II    0 1   0.00 100.00
III   1 0 100.00   0.00
V     0 1   0.00 100.00
VII  17 5  77.27  22.73
VIII  2 1  66.67  33.33
IX    7 7  50.00  50.00
X     0 1   0.00 100.00
```

```
Logistische Regression: cat.chd
                           OR 2.5 % 97.5 % p-value
(Intercept)              1.33  0.29   6.77   0.706
cat.chd: II       31908609.26  0.00     NA   0.997
cat.chd: III             0.00    NA    Inf   0.996
cat.chd: V        31908609.25  0.00     NA   0.997
cat.chd: VII             0.22  0.03   1.31   0.100
cat.chd: VIII            0.37  0.01   5.92   0.497
cat.chd: IX              0.75  0.11   4.69   0.758
cat.chd: X        31908609.26  0.00     NA   0.997
```

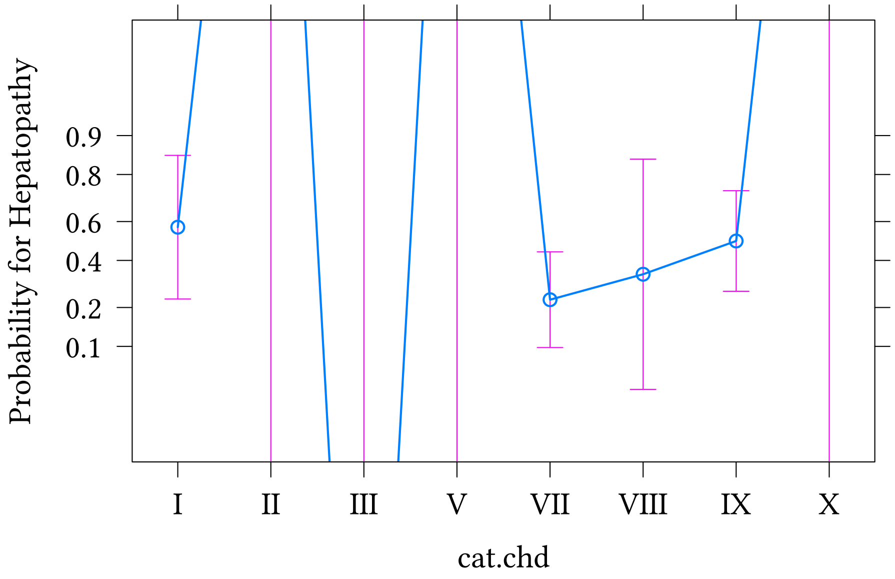

Abbildung 3: Wahrscheinlichkeit für Hepatopathy
(cat.chd.pdf, cat.chd.png)

1.4 sts_eacts

```
Häufigkeitstabelle: sts.eacts
   0  1      0      1
2  4  5 44.44  55.56
3 18  3 85.71  14.29
4  8 11 42.11  57.89
5  0  1  0.00 100.00
────────────────────
```

```
Logistische Regression: sts.eacts
              OR 2.5 % 97.5 % p-value
(Intercept) 0.14  0.01   1.76   0.144
sts.eacts   1.60  0.75   3.62   0.232
```

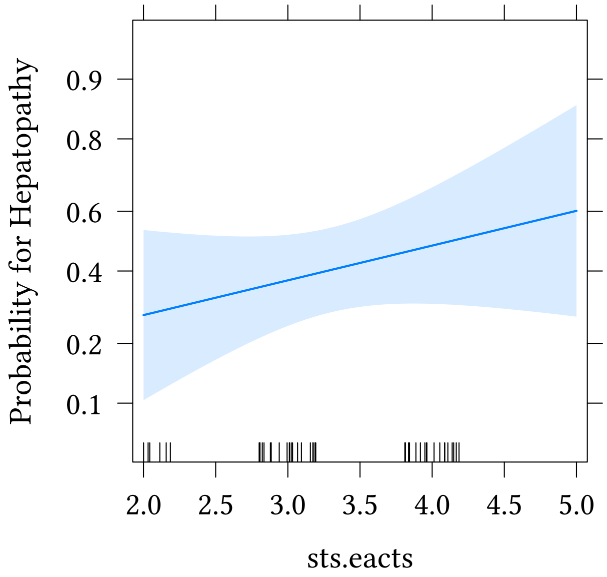

Abbildung 4: Wahrscheinlichkeit für Hepatopathy
(sts.eacts.pdf, sts.eacts.png)

1.5 unsched_reopb

```
Häufigkeitstabelle: unsched.reop
   0  1      0      1
0 26  9 74.29 25.71
1  4 11 26.67 73.33
```

```
Logistische Regression: unsched.reop
                 OR 2.5 % 97.5 % p-value
(Intercept)   0.35  0.15   0.71   0.006
unsched.reop 7.94  2.15  35.01   0.003
```

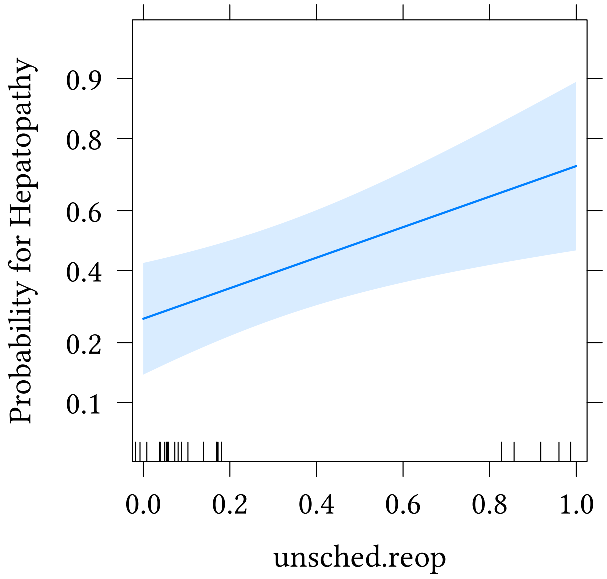

Abbildung 5: Wahrscheinlichkeit für Hepatopathy
(unsched.reop.pdf, unsched.reop.png)

1.6 hc_pre_op

```
Häufigkeitstabelle: hc.pre.op
   0 1      0      1
0 13 7 65.00 35.00
1  6 8 42.86 57.14
2 11 5 68.75 31.25
────────────────────
```

```
Logistische Regression: hc.pre.op
              OR 2.5 % 97.5 % p-value
(Intercept) 0.70  0.29   1.59   0.394
hc.pre.op   0.95  0.48   1.87   0.891
```

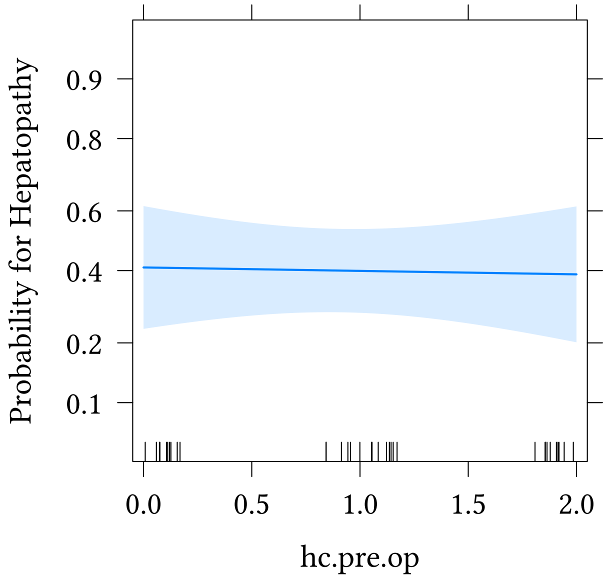

Abbildung 6: Wahrscheinlichkeit für Hepatopathy
(hc.pre.op.pdf, hc.pre.op.png)

1.7 time_cpb [h]

```
Summary: time.cpb
$`0`
  n mean   sd median 25%  75%  min  max range
 30 3.37 0.98   3.28 2.7 3.75 1.33 6.23   4.9

$`1`
  n mean  sd median 25%  75%  min  max range
 20 4.85 1.9   4.56 3.5 6.22 1.92 8.27  6.35
```

```
Logistische Regression: time.cpb
              OR 2.5 % 97.5 % p-value
(Intercept) 0.04  0.00   0.24   0.002
time.cpb    2.07  1.32   3.69   0.005
```

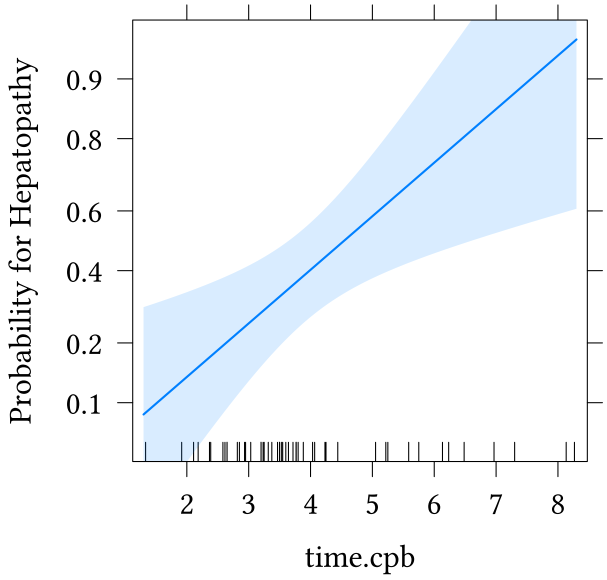

Abbildung 7: Wahrscheinlichkeit für Hepatopathy
(time.cpb.pdf, time.cpb.png)

1.8 time_act [h]

```
Summary: time.act
$`0`
  n mean   sd median 25%  75% min  max range
 30  1.9 0.64   1.87 1.6 2.11   0 3.17  3.17

$`1`
  n mean   sd median  25%  75% min  max range
 20 2.28 0.95   2.28 1.63 3.08   0 3.67  3.67
```

```
Logistische Regression: time.act
              OR 2.5 % 97.5 % p-value
(Intercept) 0.16  0.02   0.87   0.047
time.act    1.97  0.92   4.67   0.096
```

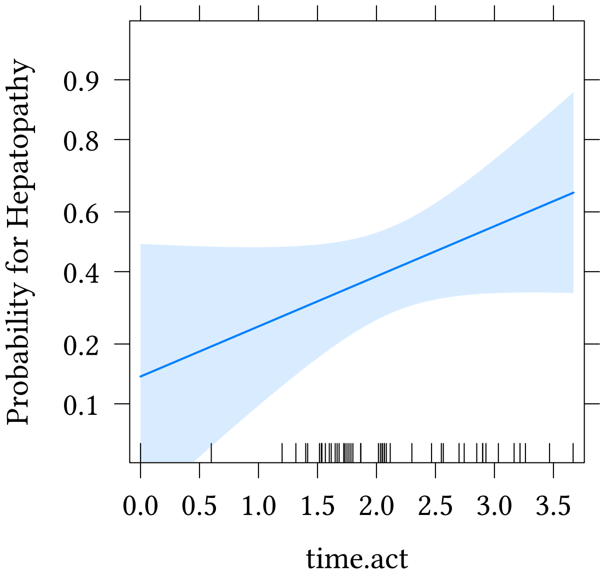

Abbildung 8: Wahrscheinlichkeit für Hepatopathy (time.act.pdf, time.act.png)

1.9 time_reperf [h]

```
Summary: time.act
$`0`
  n mean   sd median 25%  75% min  max range
 30  1.9 0.64   1.87 1.6 2.11   0 3.17  3.17

$`1`
  n mean   sd median  25%  75% min  max range
 20 2.28 0.95   2.28 1.63 3.08   0 3.67  3.67

____________________

Logistische Regression: time.act
              OR 2.5 % 97.5 % p-value
(Intercept) 0.16  0.02   0.87   0.047
time.act    1.97  0.92   4.67   0.096
```

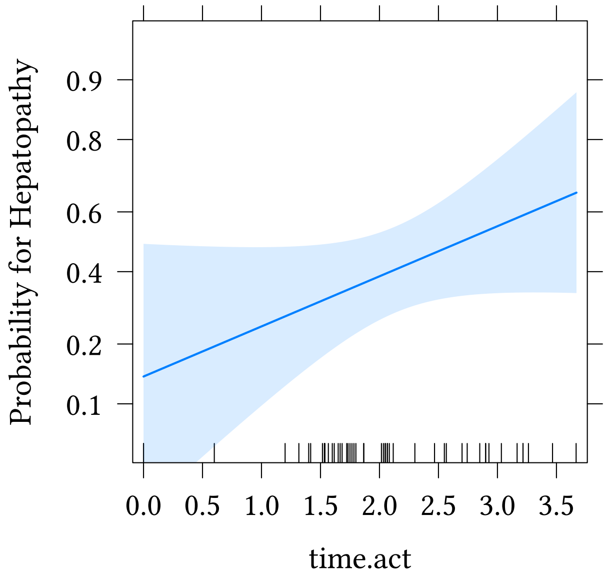

Abbildung 9: Wahrscheinlichkeit für Hepatopathy
(time.act.pdf, time.act.png)

1.10 ecmo

```
Häufigkeitstabelle: ecmo
   0  1     0     1
0 26 13 66.67 33.33
1  4  7 36.36 63.64
```

```
Logistische Regression: ecmo
              OR 2.5 % 97.5 % p-value
(Intercept) 0.50  0.25   0.96   0.041
ecmo        3.50  0.89  15.49   0.079
```

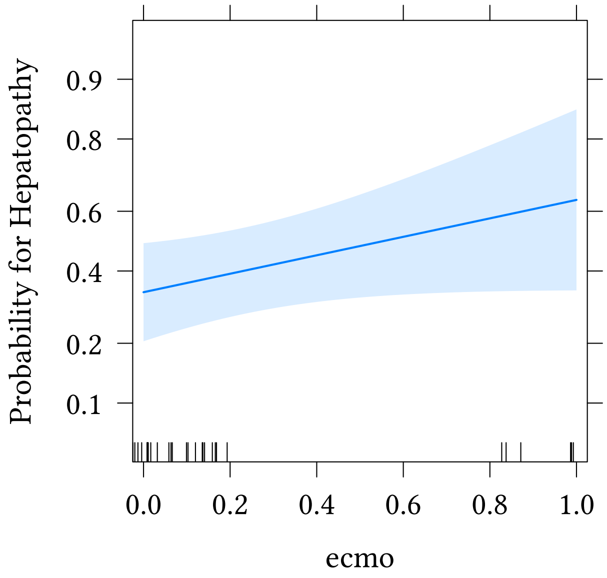

Abbildung 10: Wahrscheinlichkeit für Hepatopathy
(ecmo.pdf, ecmo.png)

1.10.1 ecmo Zeit für alle ecmo=yes

```
Summary: ecmo.dur
$`0`
 n mean   sd median  25%  75% min max range
 4    7 4.97    5.5 3.75 8.75   3  14    11

$`1`
 n mean   sd median 25% 75% min max range
 7    7 4.12      6   5   8   2  15    13

____________________

Logistische Regression: ecmo.dur
              OR 2.5 % 97.5 % p-value
(Intercept) 1.75  0.14  25.63   0.658
ecmo.dur    1.00  0.73   1.42   1.000
```

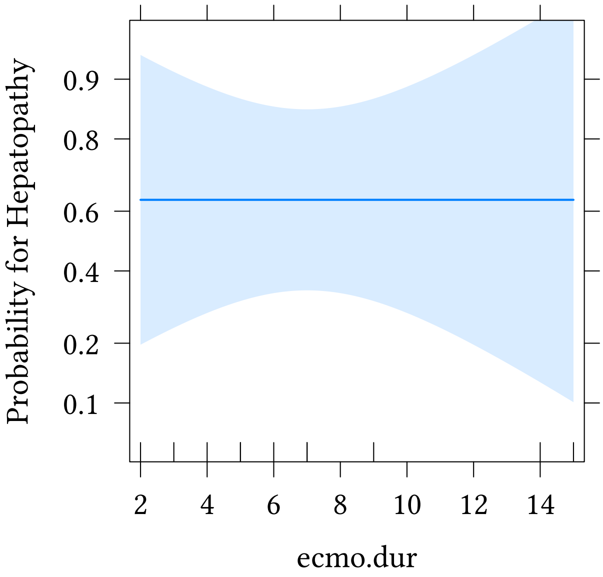

Abbildung 11: Wahrscheinlichkeit für Hepatopathy
(ecmo.dur.pdf, ecmo.dur.png)

1.11 secTC

```
Häufigkeitstabelle: secTC
     0  1     0     1
no  15  5 75.00 25.00
yes 15 15 50.00 50.00
```

```
Logistische Regression: secTC
              OR 2.5 % 97.5 % p-value
(Intercept) 0.33  0.11   0.86   0.033
secTC: yes  3.00  0.91  11.19   0.082
```

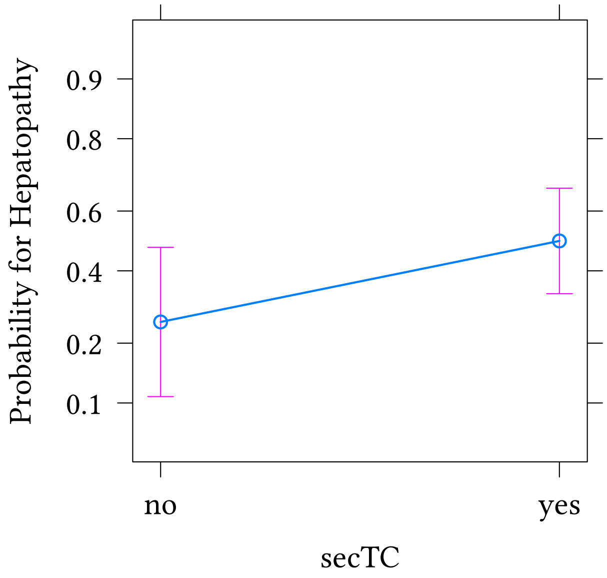

Abbildung 12: Wahrscheinlichkeit für Hepatopathy
(secTC.pdf, secTC.png)

1.12 rh.dysfct

```
Summary: rh.dysfct
$`0`
  n mean   sd median 25% 75% min max range
 30 1.07 0.74      1   1   1   0   3     3

$`1`
  n mean   sd median  25% 75% min max range
 20 2.15 0.81      2 1.75   3   1   3     2

____________________

Logistische Regression: rh.dysfct
              OR 2.5 % 97.5 % p-value
(Intercept) 0.05  0.01   0.21  <0.001
rh.dysfct   5.40  2.32  15.88  <0.001
```

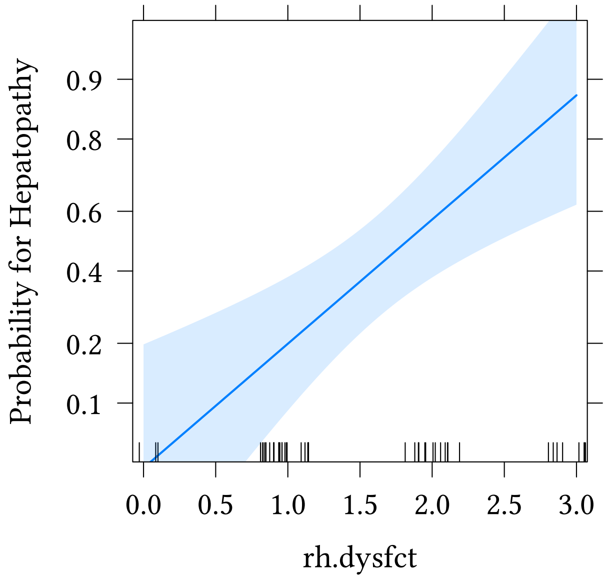

Abbildung 13: Wahrscheinlichkeit für Hepatopathy (rh.dysfct.pdf, rh.dysfct.png)

1.13 vent_duration

```
Summary: vent.duration
$`0`
  n mean   sd median  25%   75% min   max range
 30 8.34 6.9    6.96 2.31 13.38   0 22.83 22.83

$`1`
  n mean   sd median 25%  75% min max range
 20 4.77 4.98   3.88   2 5.81  -1  21    22
```

```
Logistische Regression: vent.duration
                OR 2.5 % 97.5 % p-value
(Intercept)   1.29  0.55   3.14   0.558
vent.duration 0.90  0.80   1.00   0.063
```

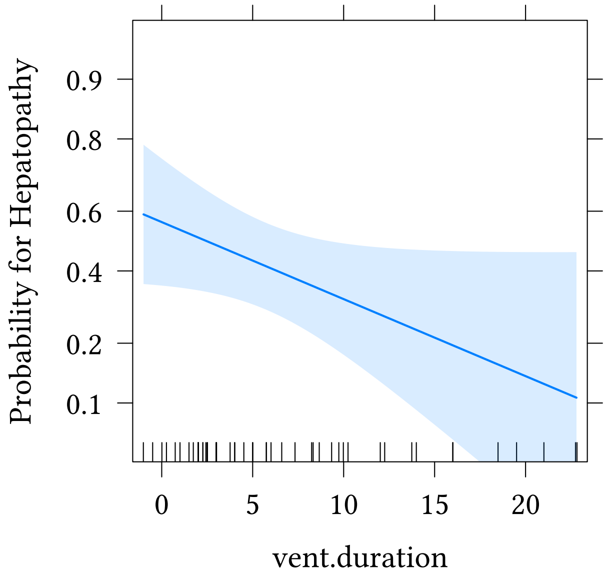

Abbildung 14: Wahrscheinlichkeit für Hepatopathy (vent.duration.pdf, vent.duration.png)

1.14 vent_before_op

```
Häufigkeitstabelle: vent.before.op
     0  1     0     1
no   23 14 62.16 37.84
yes   7  6 53.85 46.15
```

```
Logistische Regression: vent.before.op
                      OR 2.5 % 97.5 % p-value
(Intercept)         0.61  0.31   1.17   0.143
vent.before.op: yes 1.41  0.38   5.11   0.599
```

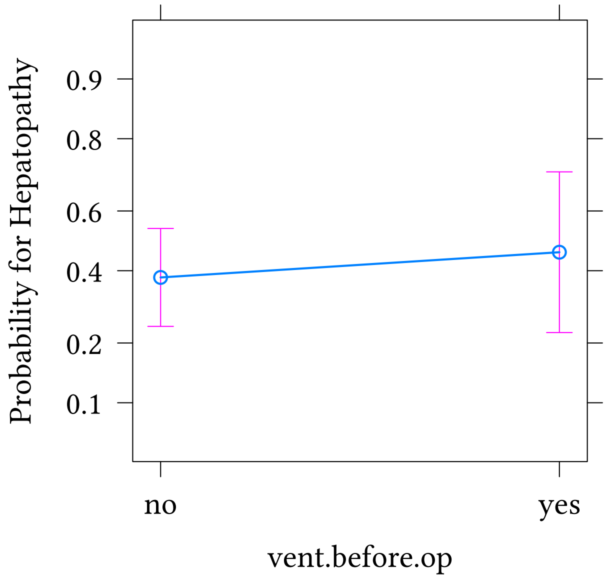

Abbildung 15: Wahrscheinlichkeit für Hepatopathy
(vent.before.op.pdf, vent.before.op.png)

1.15 dialy

```
Häufigkeitstabelle: dialy
     0  1     0     1
no  27 16 62.79 37.21
yes  3  4 42.86 57.14
```

```
Logistische Regression: dialy
              OR 2.5 % 97.5 % p-value
(Intercept) 0.59  0.31   1.09   0.097
dialy: yes  2.25  0.44  12.68   0.326
```

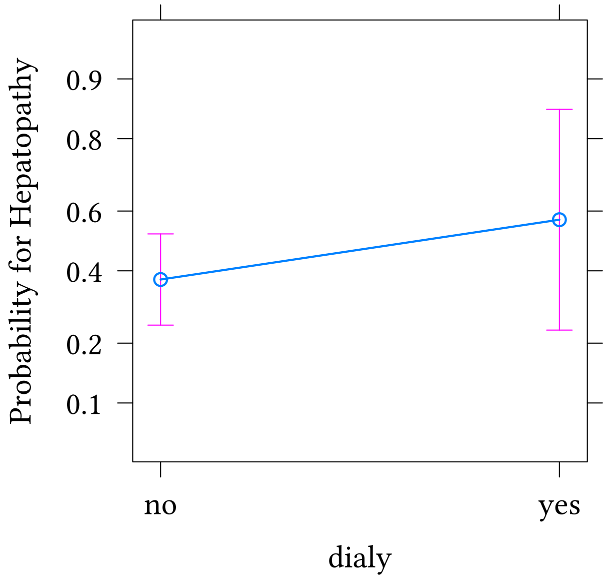

Abbildung 16: Wahrscheinlichkeit für Hepatopathy
(dialy.pdf, dialy.png)

1.15.1 dialy Zeit für alle dialy=yes

```
Summary: dialy.dur
$`0`
 n mean   sd median 25%  75% min max range
 3 8.67 6.35      5   5 10.5   5  16    11

$`1`
 n mean   sd median 25%  75% min max range
 4    8 3.56    7.5   5 10.5   5  12     7
```

```
Logistische Regression: dialy.dur
              OR 2.5 % 97.5 % p-value
(Intercept) 1.84  0.06  79.94   0.723
dialy.dur   0.96  0.65   1.42   0.833
```

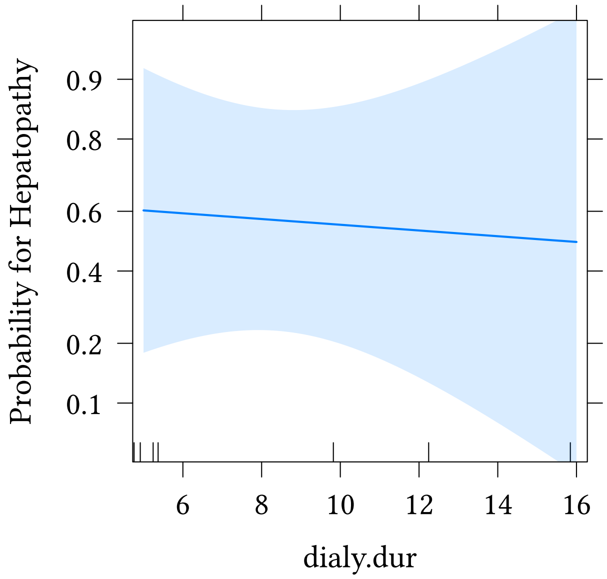

Abbildung 17: Wahrscheinlichkeit für Hepatopathy (dialy.dur.pdf, dialy.dur.png)

1.16 hosp_time

```
Summary: hosp.time
$`0`
  n mean    sd median   25%   75% min max range
 30 29.5 28.67   18.5 12.25 27.75   9 110   101

$`1`
  n  mean    sd median   25%    75% min max range
 20 101.7 72.69     78 56.25 110.25  41 299   258
```

```
Logistische Regression: hosp.time
              OR 2.5 % 97.5 % p-value
(Intercept) 0.06  0.01   0.21  <0.001
hosp.time   1.05  1.02   1.08  <0.001
```

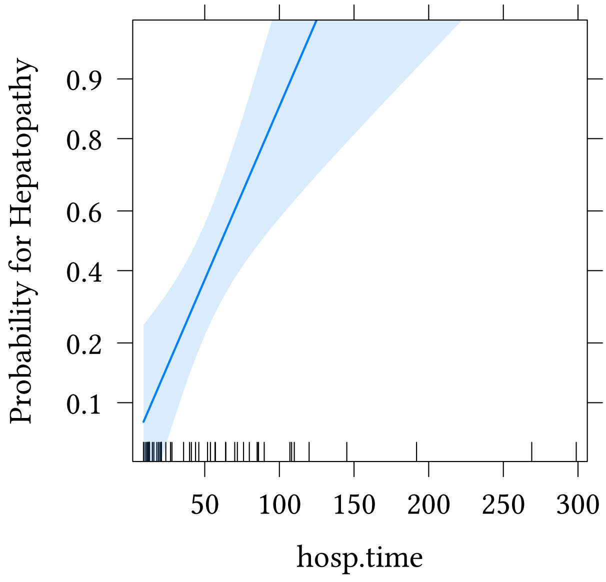

Abbildung 18: Wahrscheinlichkeit für Hepatopathy (hosp.time.pdf, hosp.time.png)

1.17 transf_ekz

```
Summary: transf.ekz
$`0`
  n   mean    sd median 25% 75% min  max range
 30 485.17 321.2    400 285 590 100 1635  1535

$`1`
  n   mean     sd median 25%  75% min  max range
 20 833.25 530.74  702.5 445 1170 200 2090  1890
```

```
Logistische Regression: transf.ekz
              OR 2.5 % 97.5 % p-value
(Intercept) 0.19  0.05   0.56   0.005
transf.ekz  1.00  1.00   1.00   0.015
```

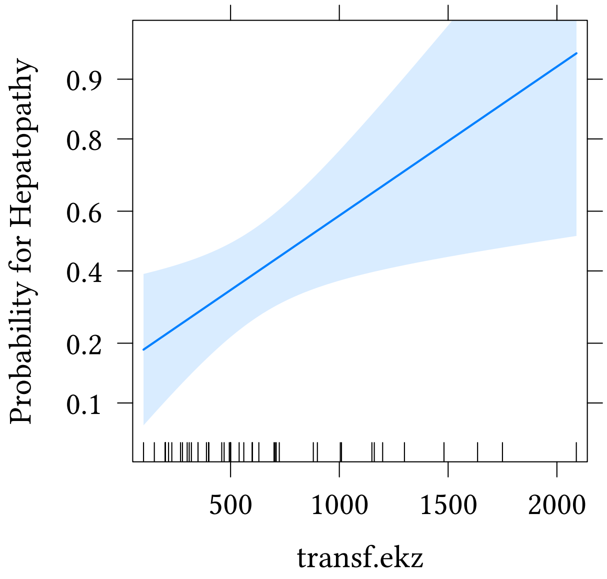

Abbildung 19: Wahrscheinlichkeit für Hepatopathy (transf.ekz.pdf, transf.ekz.png)

1.18 transf_ekz, logarithmiert

```
Summary: transf.ekz.log
$`0`
  n mean   sd median  25%  75%  min max range
 30    6 0.62   5.99 5.65 6.38 4.61 7.4   2.79

$`1`
  n mean   sd median 25%  75% min  max range
 20 6.52 0.68   6.55 6.1 7.06 5.3 7.64  2.35
```

```
Logistische Regression: transf.ekz.log
                  OR 2.5 % 97.5 % p-value
(Intercept)     0.00  0.00   0.08   0.010
transf.ekz.log 3.60  1.41  10.98   0.013
```

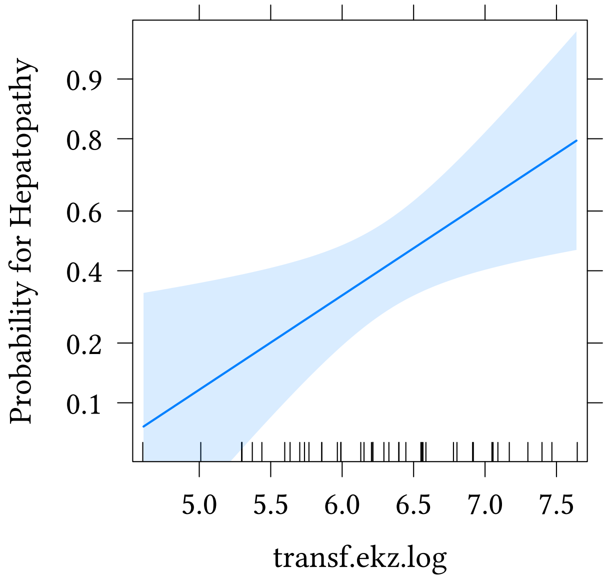

Abbildung 20: Wahrscheinlichkeit für Hepatopathy (transf.ekz.log.pdf, transf.ekz.log.png)

1.19 transf_ffp

```
Summary: transf.ffp
$`0`
  n   mean     sd median   25%   75% min  max range
 30 536.67 281.77    500 317.5 737.5 100 1200  1100

$`1`
  n   mean     sd median 25%  75% min  max range
 20 699.25 422.28    600 375 1000  80 1500  1420
```

```
Logistische Regression: transf.ffp
              OR 2.5 % 97.5 % p-value
(Intercept) 0.29  0.08   0.91   0.043
transf.ffp  1.00  1.00   1.00   0.113
```

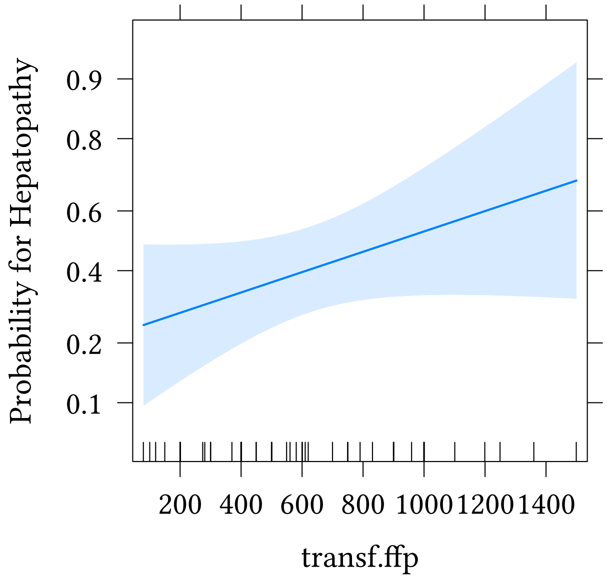

Abbildung 21: Wahrscheinlichkeit für Hepatopathy (transf.ffp.pdf, transf.ffp.png)

1.20 transf_ffp, logarithmiert

```
Summary: transf.ffp.log
$`0`
  n mean   sd median  25% 75%  min  max range
 30 6.12 0.62   6.21 5.76 6.6 4.61 7.09  2.48

$`1`
  n mean   sd median  25%  75%  min  max range
 20 6.31 0.79    6.4 5.92 6.91 4.38 7.31  2.93
```

```
Logistische Regression: transf.ffp.log
                  OR 2.5 % 97.5 % p-value
(Intercept)     0.05  0.00   9.51   0.283
transf.ffp.log 1.51  0.65   3.79   0.350
```

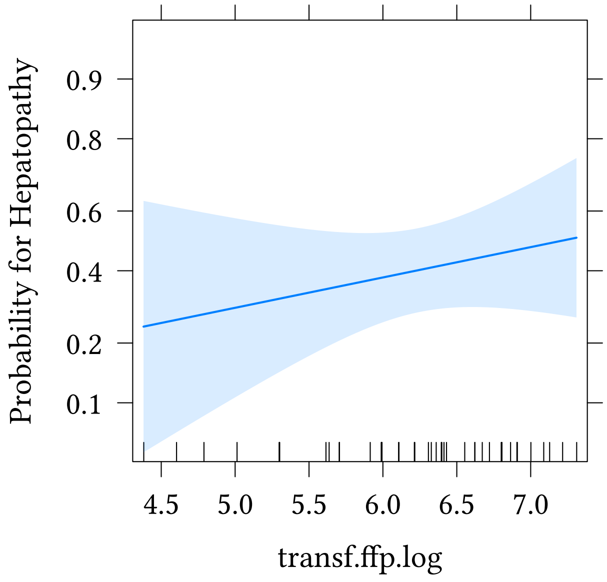

Abbildung 22: Wahrscheinlichkeit für Hepatopathy
(transf.ffp.log.pdf, transf.ffp.log.png)

1.21 transf_tkz

```
Summary: transf.tkz
$`0`
  n  mean     sd median 25% 75% min max range
 30 104.8 135.66     85  50 100   0 750   750

$`1`
  n   mean     sd median 25%   75% min  max range
 20 174.25 219.02    100  50 212.5   0 1000  1000
```

```
Logistische Regression: transf.tkz
              OR 2.5 % 97.5 % p-value
(Intercept) 0.48  0.21   0.99   0.057
transf.tkz  1.00  1.00   1.01   0.212
```

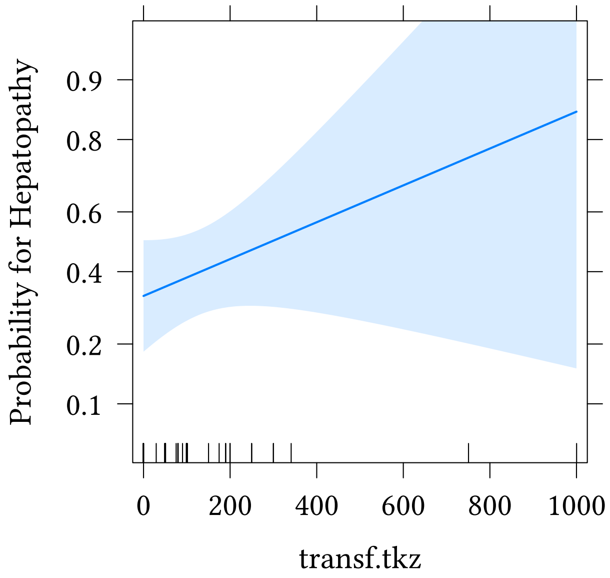

Abbildung 23: Wahrscheinlichkeit für Hepatopathy (transf.tkz.pdf, transf.tkz.png)

1.22 transf_tkz, logarithmiert

Werte von `0` werden auf `0.5` gesetzt.

```
Summary: transf.tkz.log
$`0`
  n mean   sd median  25%  75%   min  max range
 30 3.95 1.69   4.44 3.91 4.61 -0.69 6.62  7.31

$`1`
  n mean   sd median  25%  75%   min  max range
 20 4.13 2.21   4.61 3.91 5.35 -0.69 6.91   7.6

____________________

Logistische Regression: transf.tkz.log
                  OR 2.5 % 97.5 % p-value
(Intercept)     0.54  0.12   2.07   0.386
transf.tkz.log 1.05  0.78   1.47   0.750
```

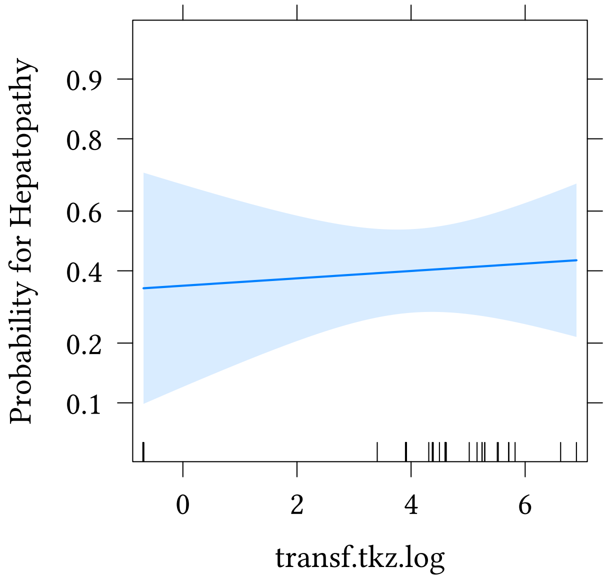

Abbildung 24: Wahrscheinlichkeit für Hepatopathy (transf.tkz.log.pdf, transf.tkz.log.png)

1.23 mix.zirk

```
Häufigkeitstabelle: mix.zirk
     0  1     0     1
no   4  8 33.33 66.67
yes 26 12 68.42 31.58
```

```
Logistische Regression: mix.zirk
                OR 2.5 % 97.5 % p-value
(Intercept)   2.00  0.63   7.49   0.258
mix.zirk: yes 0.23  0.05   0.88   0.037
```

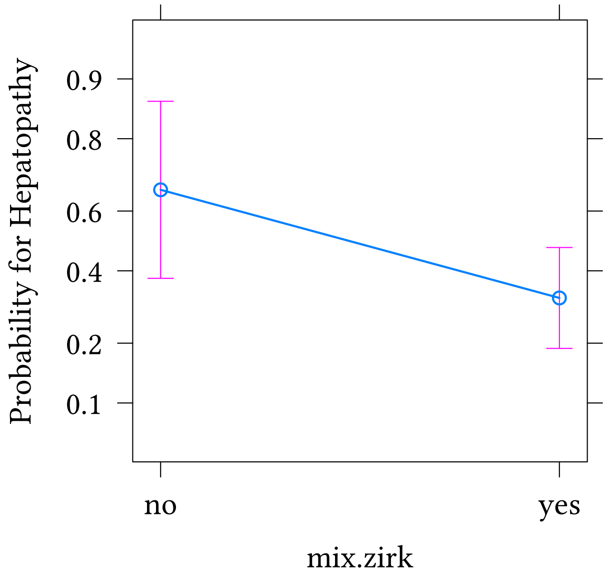

Abbildung 25: Wahrscheinlichkeit für Hepatopathy (mix.zirk.pdf, mix.zirk.png)

1.24 zvs_min

```
Summary: zvs.min
$`0`
  n  mean     sd median   25%   75%  min max range
 30 55.75 12.37  57.25 50.03 64.55 29.1  73  43.9

$`1`
  n  mean   sd median   25%   75%  min  max range
 20 45.22 9.65  46.15 37.43 50.68 28.9 61.6  32.7

____________________

Logistische Regression: zvs.min
               OR 2.5 % 97.5 % p-value
(Intercept) 37.75  2.52 901.20   0.014
zvs.min      0.92  0.87   0.97   0.006
```

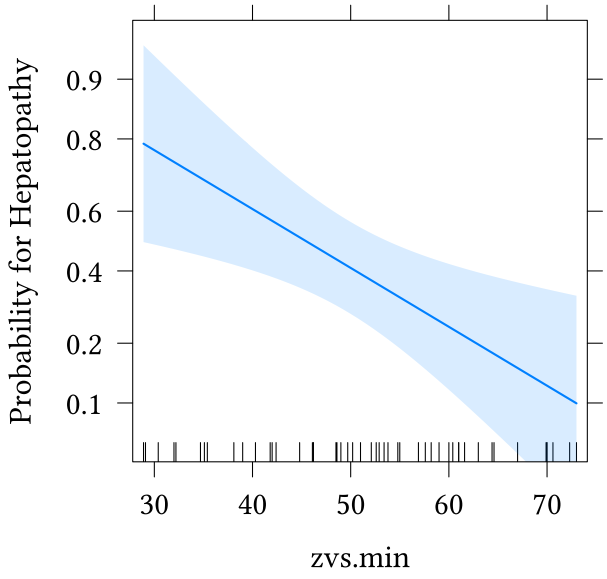

Abbildung 26: Wahrscheinlichkeit für Hepatopathy
(zvs.min.pdf, zvs.min.png)

1.25 lakt_max

```
Summary: lakt.max
$`0`
  n mean   sd median 25%  75% min  max range
 30 4.51 3.08    3.2 2.5 5.93 1.2 14.2    13

$`1`
  n mean   sd median  25%  75% min max range
 20 7.04 4.03    6.4 4.38 8.72 1.5  15  13.5
```

```
Logistische Regression: lakt.max
              OR 2.5 % 97.5 % p-value
(Intercept) 0.21  0.06   0.64   0.009
lakt.max    1.23  1.04   1.50   0.025
```

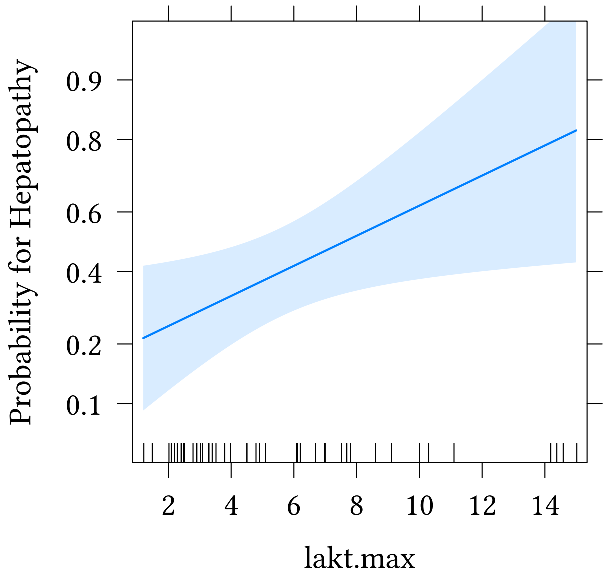

Abbildung 27: Wahrscheinlichkeit für Hepatopathy (lakt.max.pdf, lakt.max.png)

1.26 lak_norm

```
Summary: lak.norm
$`0`
  n mean   sd median 25% 75% min max range
 30  1.1 1.03      1   1   1   0   5     5

$`1`
  n mean   sd median 25% 75% min max range
 20  3.1 4.14    1.5   1   3   0  16    16
```

```
Logistische Regression: lak.norm
              OR 2.5 % 97.5 % p-value
(Intercept) 0.28  0.10   0.69   0.011
lak.norm    1.70  1.12   3.10   0.049
```

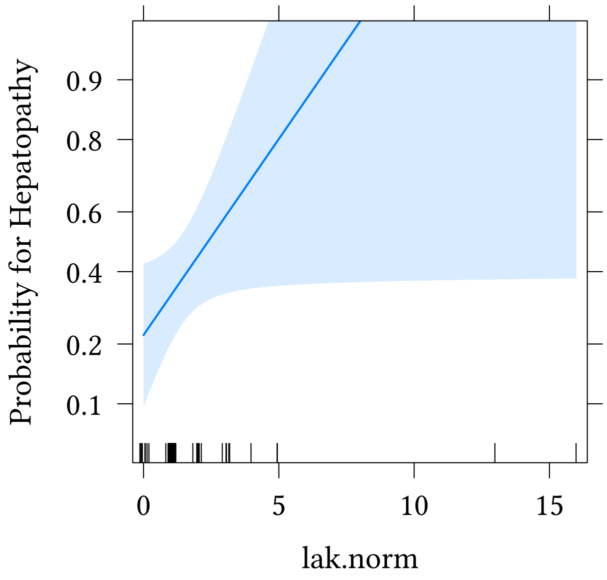

Abbildung 28: Wahrscheinlichkeit für Hepatopathy (lak.norm.pdf, lak.norm.png)

1.27 infect

```
Häufigkeitstabelle: infect
   0  1     0     1
0 22  5 81.48 18.52
1  8 15 34.78 65.22
```

```
Logistische Regression: infect
              OR 2.5 % 97.5 % p-value
(Intercept) 0.23  0.08   0.55   0.003
infect      8.25  2.39  32.92   0.001
```

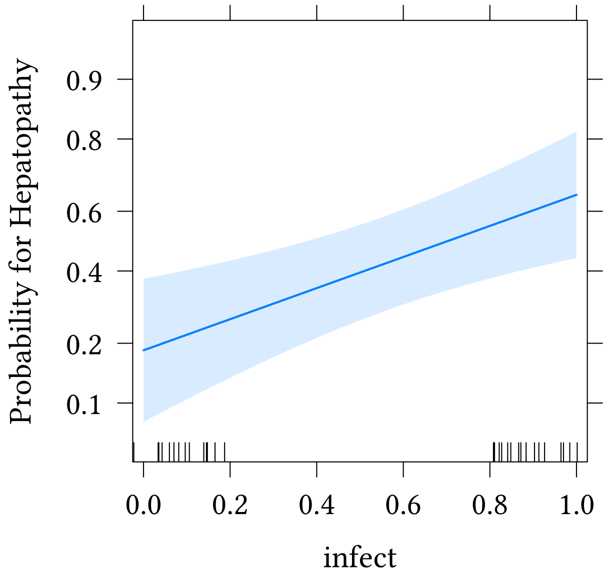

Abbildung 29: Wahrscheinlichkeit für Hepatopathy
(infect.pdf, infect.png)

1.28 bnp.min

```
Summary: bnp.min
$`0`
  n   mean    sd median    25%   75% min   max range
 30 7884.1 10432 3476.5 484.75 11774 107 41711 41604

$`1`
  n   mean    sd median   25%   75% min   max range
 20 8995.5 10210   5021 969.5 11410 177 32616 32439

___________________

Logistische Regression: bnp.min
             OR 2.5 % 97.5 % p-value
(Intercept) 0.61  0.28   1.26   0.187
bnp.min     1.00  1.00   1.00   0.705
```

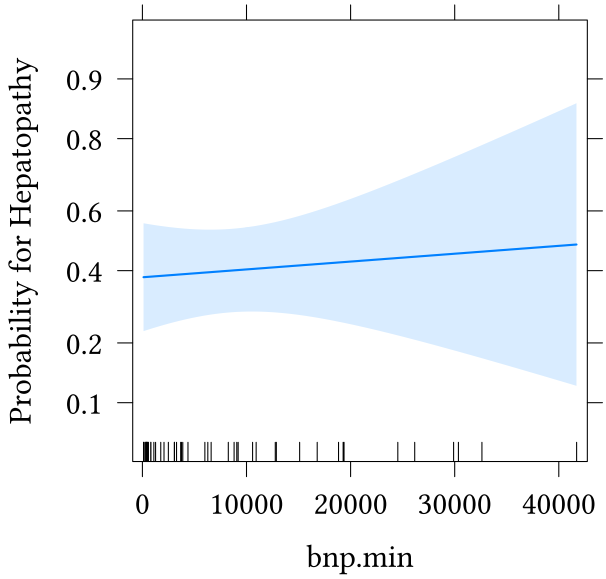

Abbildung 30: Wahrscheinlichkeit für Hepatopathy
(bnp.min.pdf, bnp.min.png)

1.29 bnp.max

```
Summary: bnp.max
$`0`
  n  mean    sd median   25%   75%  min    max  range
 30 32556 29232  25584 13087 39267 4534 121392 116858

$`1`
  n   mean     sd median   25%   75%  min     max   range
 20 123267 298174  44341 29384 78562 3678 1375766 1372088
```

```
Logistische Regression: bnp.max
              OR 2.5 % 97.5 % p-value
(Intercept) 0.28  0.10   0.70   0.011
bnp.max     1.00  1.00   1.00   0.044
```

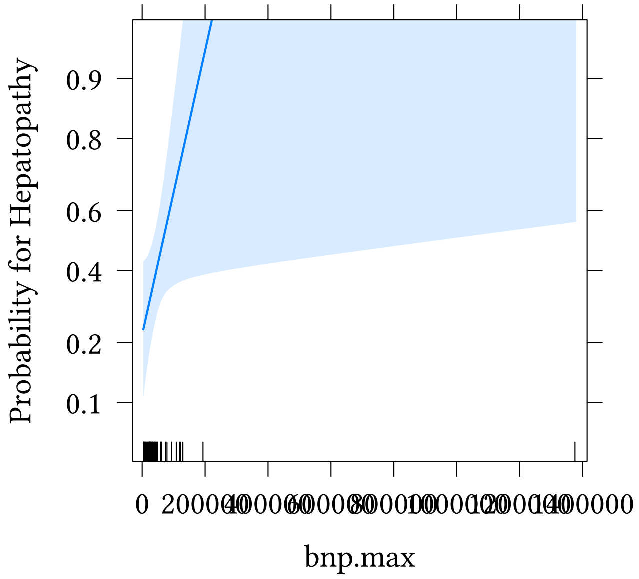

Abbildung 31: Wahrscheinlichkeit für Hepatopathy
(bnp.max.pdf, bnp.max.png)

1.30 tnt.min

```
Summary: tnt.min
$`0`
  n  mean    sd median 25% 75% min max range missing
 29 50.69 52.96     20  12  85   7 184   177       1

$`1`
  n   mean     sd median 25%  75%  min max range
 20 -12.75 234.28     32 7.5 51.5 -999 109  1108
```

____

```
Logistische Regression: tnt.min
              OR 2.5 % 97.5 % p-value
(Intercept) 0.86  0.43   1.96   0.708
tnt.min     0.99  0.98   1.00   0.353
```

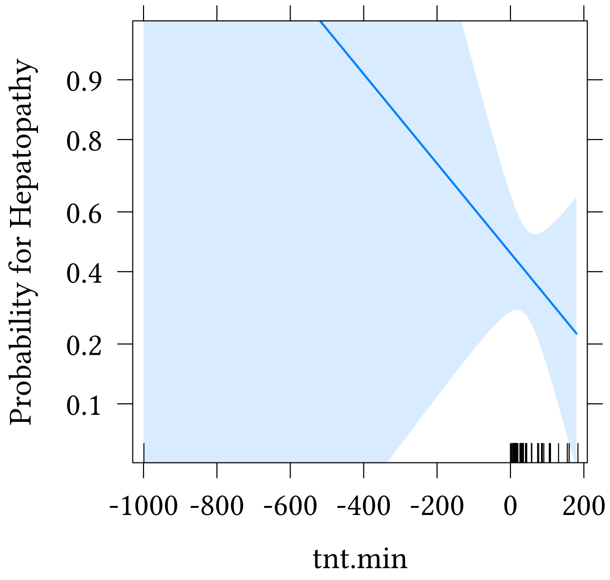

Abbildung 32: Wahrscheinlichkeit für Hepatopathy
(tnt.min.pdf, tnt.min.png)

1.31 tnt.max

```
Summary: tnt.max
$`0`
  n   mean     sd median    25%    75% min   max range
 30 5757.6 3315.3 4731.5 3284.5 9736.2  62 10000  9938

$`1`
  n   mean     sd median    25%   75% min   max range
 20 6507.3 3435.6   6011 3693.2 10000  33 10000  9967

____________________

Logistische Regression: tnt.max
              OR 2.5 % 97.5 % p-value
(Intercept) 0.44  0.12   1.42   0.182
tnt.max     1.00  1.00   1.00   0.435
```

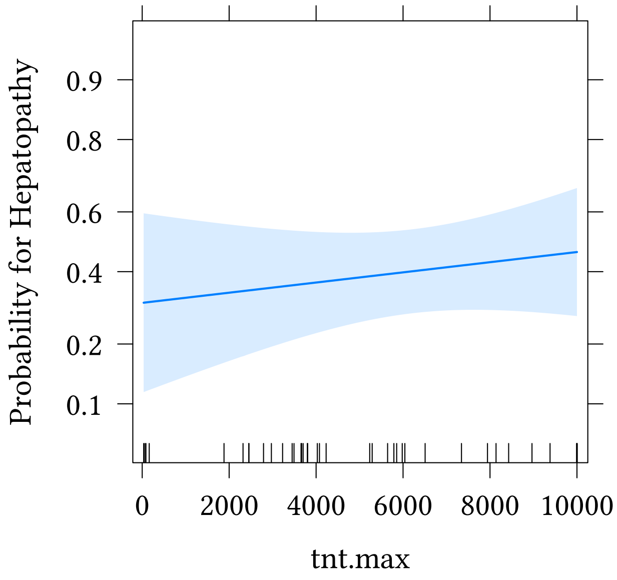

Abbildung 33: Wahrscheinlichkeit für Hepatopathy
(tnt.max.pdf, tnt.max.png)

1.32 gldh.min

```
Summary: gldh.min
$`0`
  n    mean     sd median 25% 75%  min max range
 30 -191.44 410.75   5.55   5  12 -999  42  1041

$`1`
  n mean   sd median 25%  75% min  max range
 20 8.29 5.19   6.15   5 9.12   0 19.3  19.3

____________________

Logistische Regression: gldh.min
              OR 2.5 % 97.5 % p-value
(Intercept) 0.79  0.39   1.43   0.444
gldh.min    1.01  1.00     NA   0.391
```

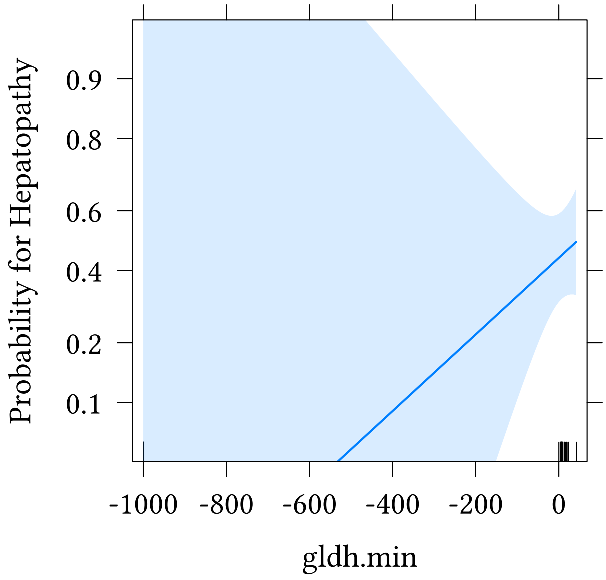

Abbildung 34: Wahrscheinlichkeit für Hepatopathy
(gldh.min.pdf, gldh.min.png)

1.33 gldh.max

```
Summary: gldh.max
$`0`
  n  mean     sd median   25%   75% min max range
 30 98.89 214.34  19.25 10.12 50.05   5 947   942

$`1`
  n   mean     sd median   25%    75% min max range
 20 230.64 185.14  162.5 93.07 395.75 5.9 588 582.1
```

```
Logistische Regression: gldh.max
              OR 2.5 % 97.5 % p-value
(Intercept) 0.41  0.18   0.84   0.019
gldh.max    1.00  1.00   1.01   0.045
```

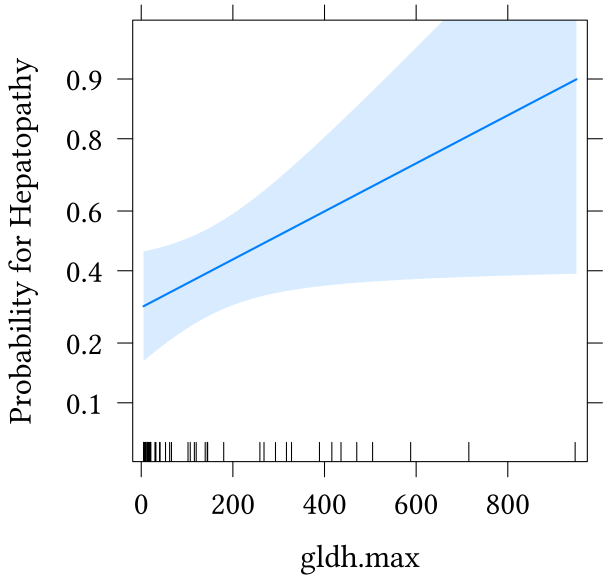

Abbildung 35: Wahrscheinlichkeit für Hepatopathy (gldh.max.pdf, gldh.max.png)

1.34 quick.min

```
Summary: quick.min
$`0`
  n  mean    sd median   25%   75% min   max range
 30 51.66 20.06   47.1 39.65 59.85  21 112.4  91.4

$`1`
  n  mean    sd median   25%   75% min max range
 20 32.94 19.73   30.5 16.75 49.17 7.7  68  60.3
```

```
Logistische Regression: quick.min
              OR 2.5 % 97.5 % p-value
(Intercept) 6.22  1.36  38.32   0.029
quick.min   0.95  0.91   0.98   0.005
```

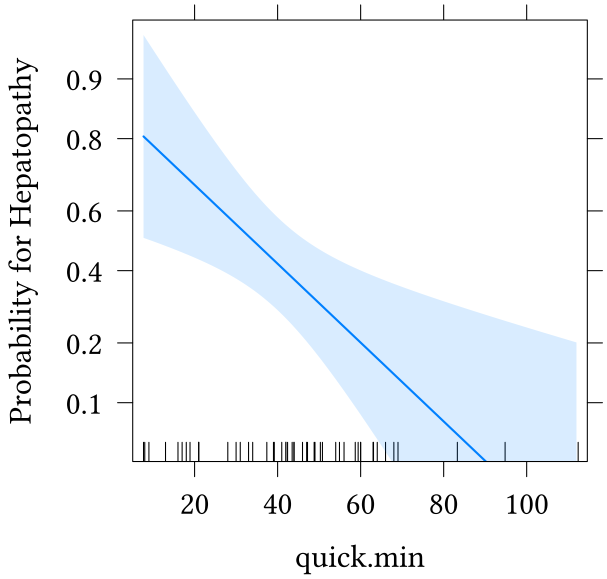

Abbildung 36: Wahrscheinlichkeit für Hepatopathy (quick.min.pdf, quick.min.png)

1.35 alb.min

```
Summary: alb.min
$`0`
  n   mean     sd median   25%   75%  min max range missing
 24 -97.71 348.03  28.15 24.55 35.25 -999  40  1039       6

$`1`
  n   mean     sd median   25%   75%  min max range
 20 -79.09 314.63     22 18.52 25.62 -999  31  1030
```

```
Logistische Regression: alb.min
              OR 2.5 % 97.5 % p-value
(Intercept) 0.85  0.45   1.56   0.595
alb.min     1.00  1.00   1.00   0.850
```

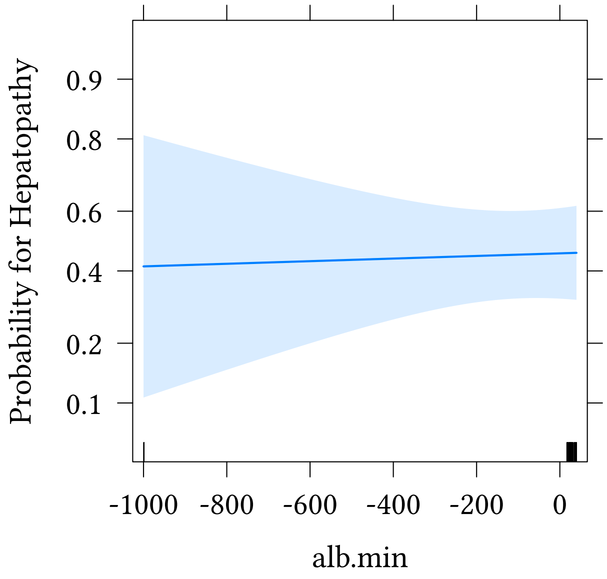

Abbildung 37: Wahrscheinlichkeit für Hepatopathy
(alb.min.pdf, alb.min.png)

1.36 bili.max

```
Summary: bili.max
$`0`
  n mean   sd median 25%  75% min max range
 30 2.44 2.46   1.45   1 3.33 0.4  10   9.6

$`1`
  n mean   sd median  25%   75% min max range
 20  7.8 6.76      6 1.82 11.55 1.3  22  20.7
```

```
Logistische Regression: bili.max
              OR 2.5 % 97.5 % p-value
(Intercept) 0.19  0.07   0.49   0.001
bili.max    1.34  1.13   1.70   0.005
```

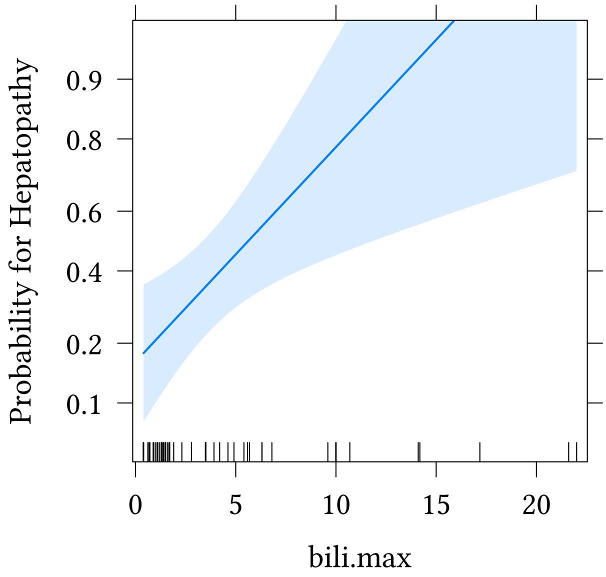

Abbildung 38: Wahrscheinlichkeit für Hepatopathy (bili.max.pdf, bili.max.png)

1.37 got.max

```
Summary: got.max
$`0`
  n  mean     sd median 25% 75% min  max range
 30 355.2 669.97    165 100 228  38 2866  2828

$`1`
  n   mean     sd median 25%    75% min  max range
 20 602.25 600.87  341.5 217 808.25 105 2265  2160
```

```
Logistische Regression: got.max
              OR 2.5 % 97.5 % p-value
(Intercept) 0.51  0.24   1.01   0.060
got.max     1.00  1.00   1.00   0.206
```

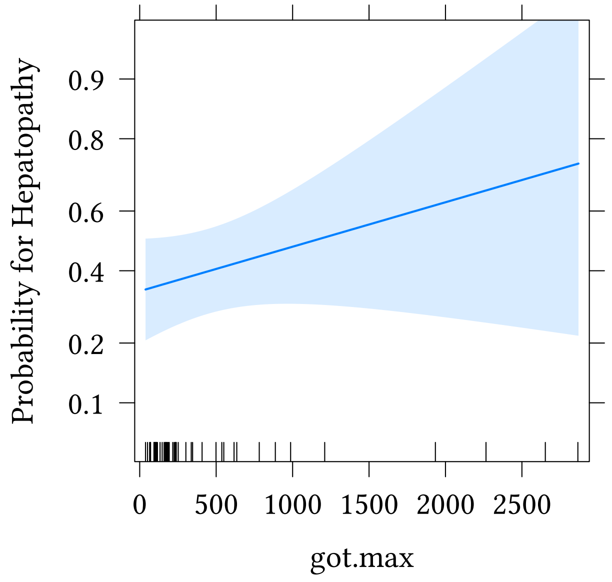

Abbildung 39: Wahrscheinlichkeit für Hepatopathy
(got.max.pdf, got.max.png)

1.38 gpt.max

```
Summary: gpt.max
$`0`
  n  mean     sd median   25%   75% min  max range
 30 153.2 361.4      36 23.25 70.25  19 1795  1776

$`1`
  n  mean      sd median   25%    75% min  max range
 20 425.6 555.11  208.5 101.5 427.75   6 2121  2115
```

```
Logistische Regression: gpt.max
              OR 2.5 % 97.5 % p-value
(Intercept) 0.46  0.22   0.90   0.027
gpt.max     1.00  1.00   1.00   0.072
```

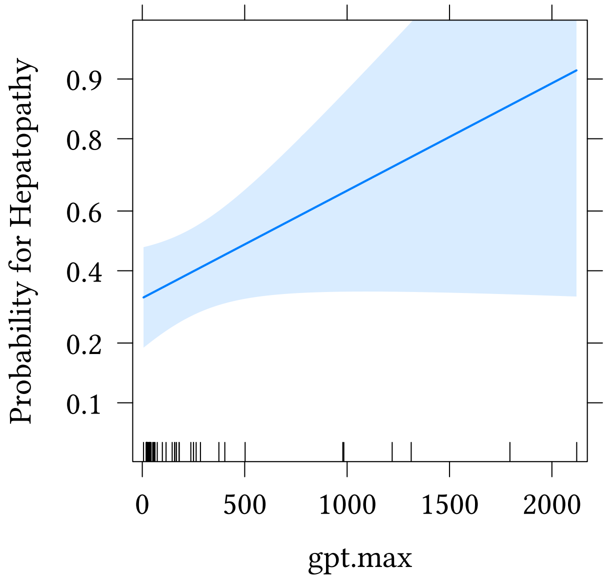

Abbildung 40: Wahrscheinlichkeit für Hepatopathy
(gpt.max.pdf, gpt.max.png)

1.39 ggt.max

```
Summary: ggt.max
$`0`
  n  mean      sd median 25% 75%  min max range missing
 29 21.52 326.17     39  24  72 -999 698  1697       1

$`1`
  n   mean      sd median    25%    75% min  max range
 20 2120.2 1471.5 1804.5 1065.8 3157.5  24 5290  5266
```

```
Logistische Regression: ggt.max
              OR 2.5 % 97.5 % p-value
(Intercept) 0.06  0.01   0.21  <0.001
ggt.max     1.00  1.00   1.01   0.002
```

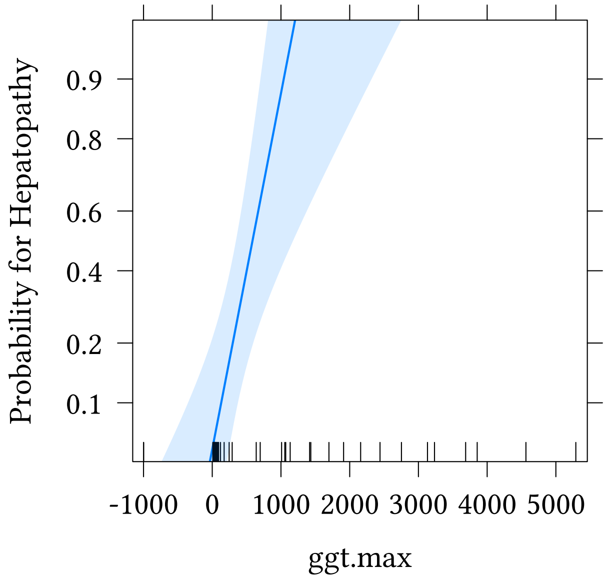

Abbildung 41: Wahrscheinlichkeit für Hepatopathy
(ggt.max.pdf, ggt.max.png)

1.40 proc.rel.fact

1.41 proc.rel.fact.ple

```
Häufigkeitstabelle: proc.rel.fact.ple
     0  1     0     1
no  23  6 79.31 20.69
yes  7 14 33.33 66.67
```

```
Logistische Regression: proc.rel.fact.ple
                         OR 2.5 % 97.5 % p-value
(Intercept)            0.26  0.10   0.60   0.003
proc.rel.fact.ple: yes 7.67  2.25  29.63   0.002
```

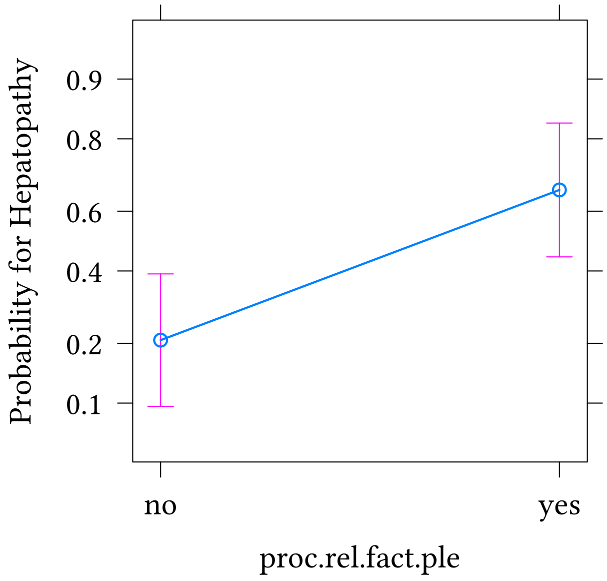

Abbildung 42: Wahrscheinlichkeit für Hepatopathy
(proc.rel.fact.ple.pdf, proc.rel.fact.ple.png)

1.42 proc.rel.fact.cyt

```
Häufigkeitstabelle: proc.rel.fact.cyt
     0  1     0     1
no  26 15 63.41 36.59
yes  4  5 44.44 55.56
```

```
Logistische Regression: proc.rel.fact.cyt
                         OR 2.5 % 97.5 % p-value
(Intercept)            0.58  0.30   1.08   0.090
proc.rel.fact.cyt: yes 2.17  0.50   9.98   0.299
```

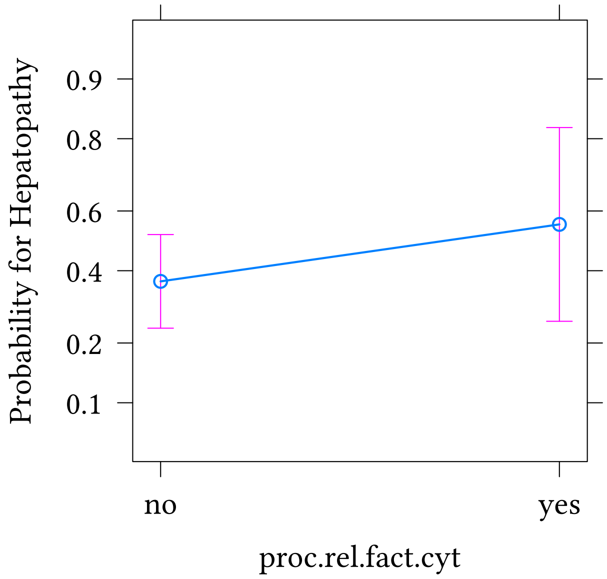

Abbildung 43: Wahrscheinlichkeit für Hepatopathy
(proc.rel.fact.cyt.pdf, proc.rel.fact.cyt.png)

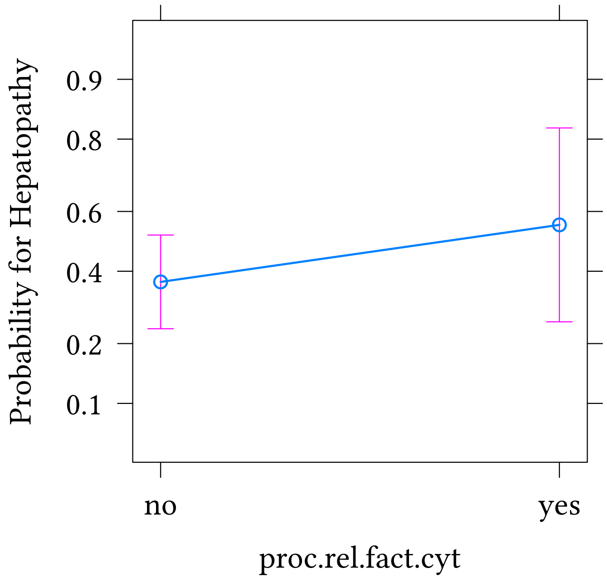

Abbildung 44: Wahrscheinlichkeit für Hepatopathy
(proc.rel.fact.cyt.pdf, proc.rel.fact.cyt.png)

1.43 proc.rel.fact.diap

```
Häufigkeitstabelle: proc.rel.fact.diap
     0  1     0     1
no  28 16 63.64 36.36
yes  2  4 33.33 66.67
```

```
Logistische Regression: proc.rel.fact.diap
                          OR 2.5 % 97.5 % p-value
(Intercept)             0.57  0.30   1.04   0.074
proc.rel.fact.diap: yes 3.50  0.61  27.33   0.174
```

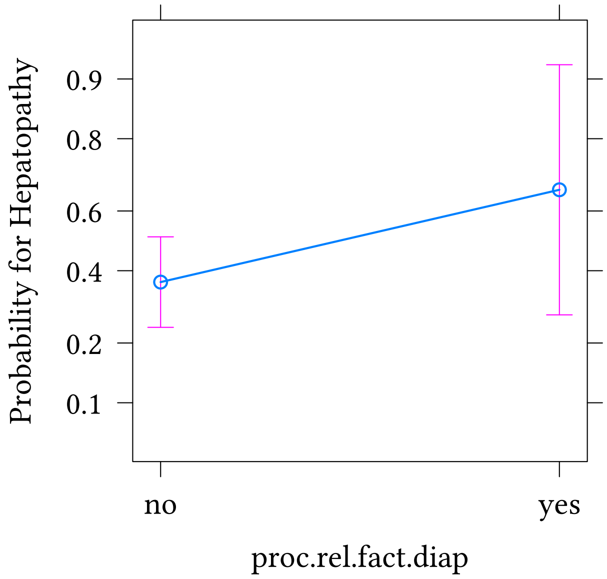

Abbildung 45: Wahrscheinlichkeit für Hepatopathy
(proc.rel.fact.diap.pdf, proc.rel.fact.diap.png)

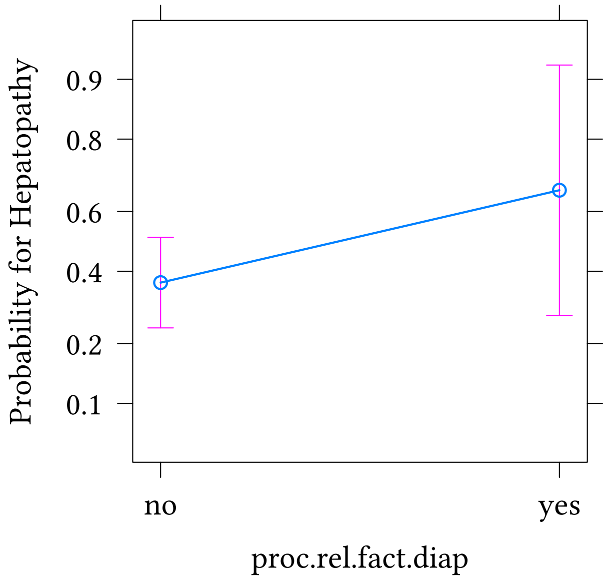

Abbildung 46: Wahrscheinlichkeit für Hepatopathy
(proc.rel.fact.diap.pdf, proc.rel.fact.diap.png)

1.44 proc.rel.fact.throm

```
Häufigkeitstabelle: proc.rel.fact.throm
      0  1     0     1
no   26 19 57.78 42.22
yes   4  1 80.00 20.00
```

```
Logistische Regression: proc.rel.fact.throm
                          OR 2.5 % 97.5 % p-value
(Intercept)             0.73  0.40   1.31   0.299
proc.rel.fact.throm: yes 0.34  0.02   2.55   0.354
```

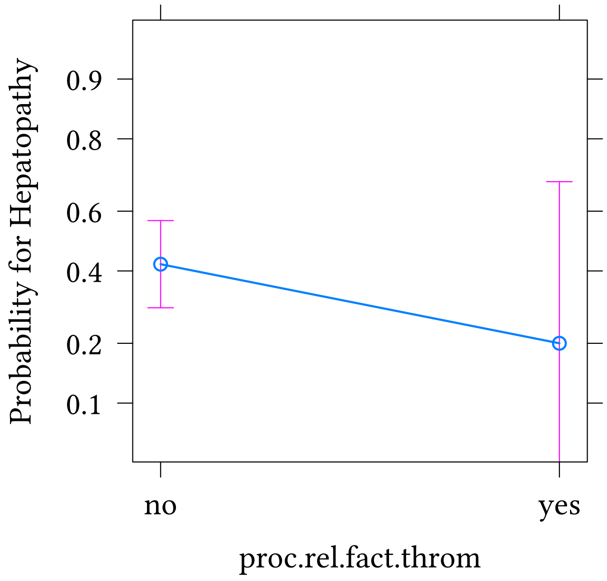

Abbildung 47: Wahrscheinlichkeit für Hepatopathy
(proc.rel.fact.throm.pdf, proc.rel.fact.throm.png)

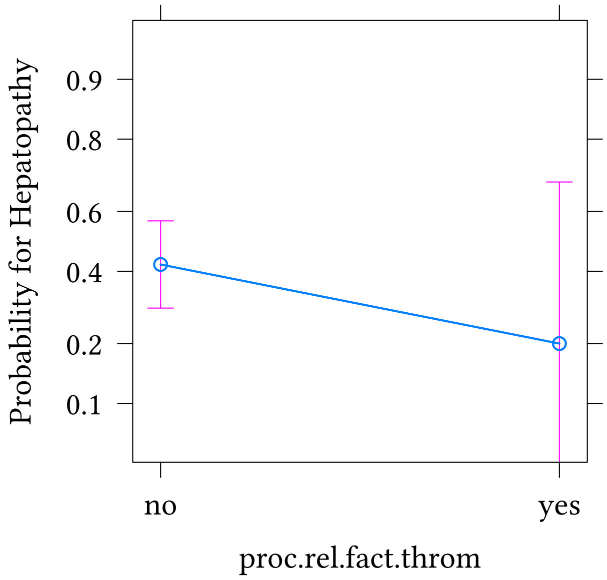

Abbildung 48: Wahrscheinlichkeit für Hepatopathy
(proc.rel.fact.throm.pdf, proc.rel.fact.throm.png)

1.45 proc.rel.fact.hrs

```
Häufigkeitstabelle: proc.rel.fact.hrs
     0  1     0     1
no  19  4 82.61 17.39
yes 11 16 40.74 59.26
```

```
Logistische Regression: proc.rel.fact.hrs
                          OR 2.5 % 97.5 % p-value
(Intercept)             0.21  0.06   0.56   0.005
proc.rel.fact.hrs: yes 6.91  1.97  29.15   0.004
```

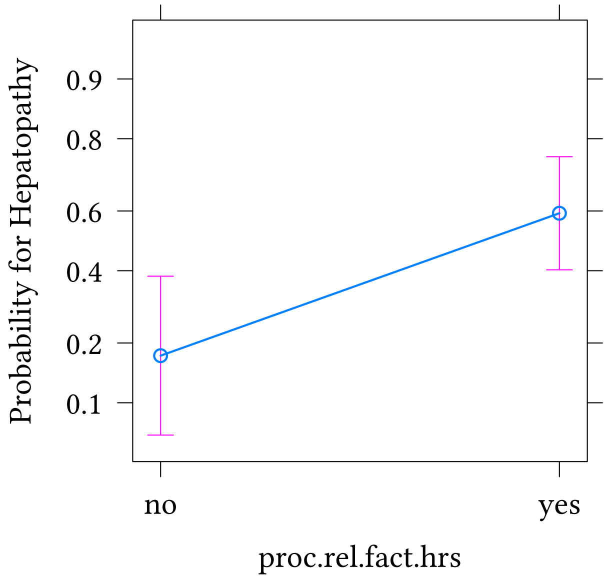

Abbildung 49: Wahrscheinlichkeit für Hepatopathy
(proc.rel.fact.hrs.pdf, proc.rel.fact.hrs.png)

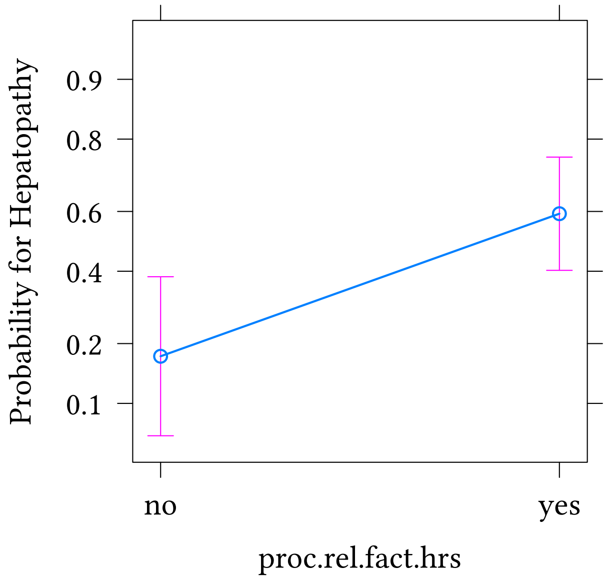

Abbildung 50: Wahrscheinlichkeit für Hepatopathy
(proc.rel.fact.hrs.pdf, proc.rel.fact.hrs.png)

1.46 use.nor

```
Häufigkeitstabelle: use.nor
     0  1     0     1
no  25 14 64.10 35.90
yes  5  6 45.45 54.55
```

```
Logistische Regression: use.nor
               OR 2.5 % 97.5 % p-value
(Intercept)  0.56  0.28   1.06   0.082
use.nor: yes 2.14  0.55   8.70   0.270
```

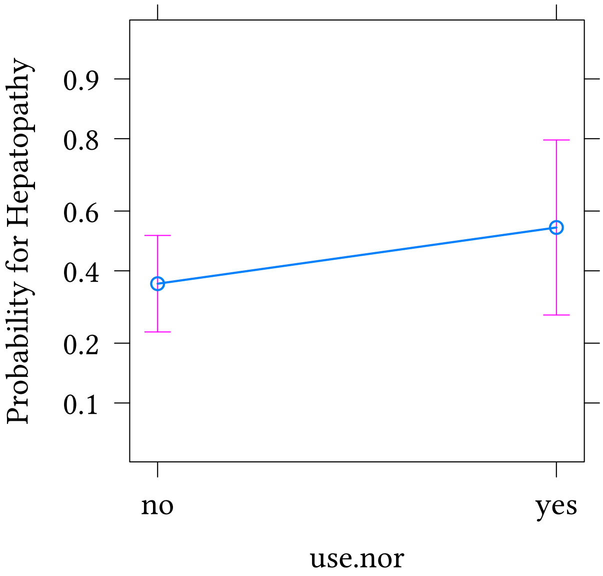

Abbildung 51: Wahrscheinlichkeit für Hepatopathy (use.nor.pdf, use.nor.png)

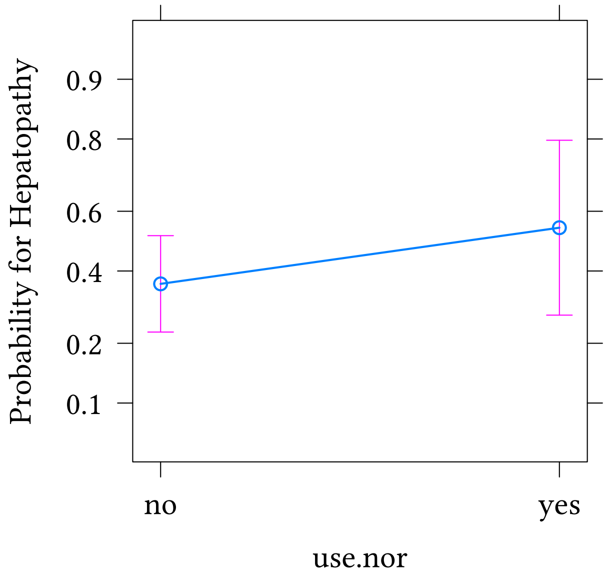

Abbildung 52: Wahrscheinlichkeit für Hepatopathy
(use.nor.pdf, use.nor.png)

1.47 use.supra

```
Häufigkeitstabelle: use.supra
     0  1     0     1
no   5  1 83.33 16.67
yes 25 19 56.82 43.18
```

```
Logistische Regression: use.supra
                 OR 2.5 % 97.5 % p-value
(Intercept)     0.20  0.01   1.24   0.142
use.supra: yes 3.80  0.55  76.04   0.240
```

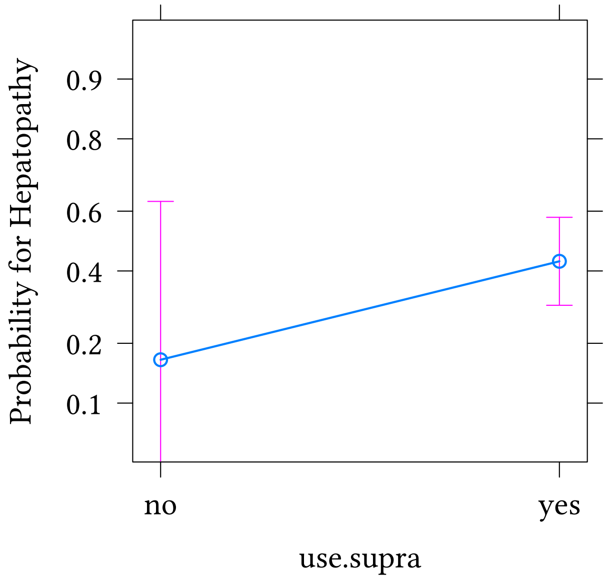

Abbildung 53: Wahrscheinlichkeit für Hepatopathy
(use.supra.pdf, use.supra.png)

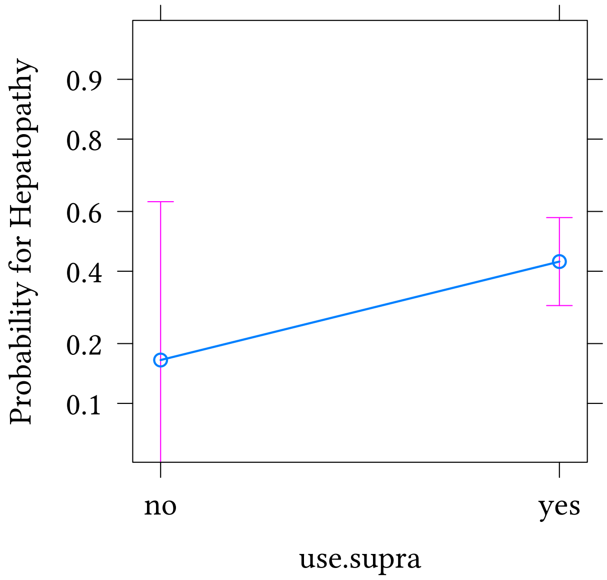

Abbildung 54: Wahrscheinlichkeit für Hepatopathy
(`use.supra.pdf`, `use.supra.png`)

1.48 use.milr

```
Häufigkeitstabelle: use.milr
     0  1     0     1
no   1  1 50.00 50.00
yes 29 19 60.42 39.58
```

```
Logistische Regression: use.milr
                  OR 2.5 % 97.5 % p-value
(Intercept)     1.00  0.04  25.29   1.000
use.milr: yes 0.66  0.02  17.24   0.770
```

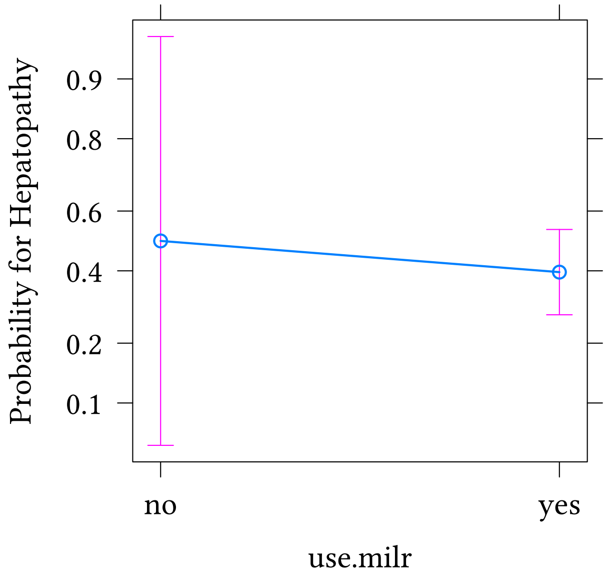

Abbildung 55: Wahrscheinlichkeit für Hepatopathy (use.milr.pdf, use.milr.png)

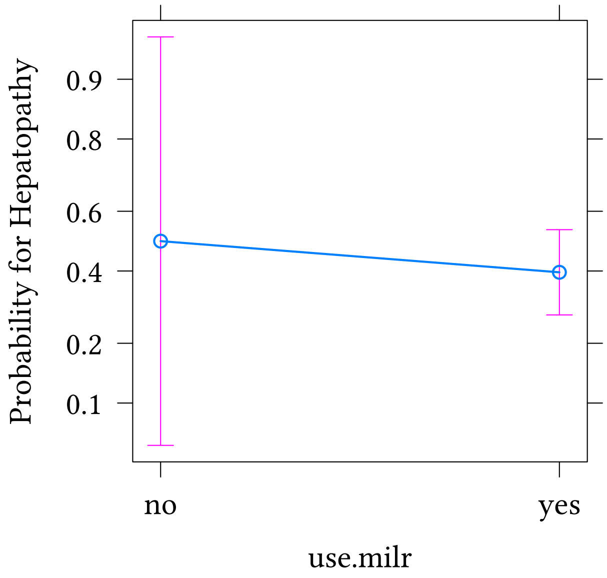

Abbildung 56: Wahrscheinlichkeit für Hepatopathy
(use.milr.pdf, use.milr.png)

1.49 tpn

```
Häufigkeitstabelle: tpn
     0  1     0     1
no  24  4 85.71 14.29
yes  6 16 27.27 72.73
```

```
Logistische Regression: tpn
               OR 2.5 % 97.5 % p-value
(Intercept)  0.17  0.05   0.43  <0.001
tpn: yes    16.00  4.24  74.64  <0.001
```

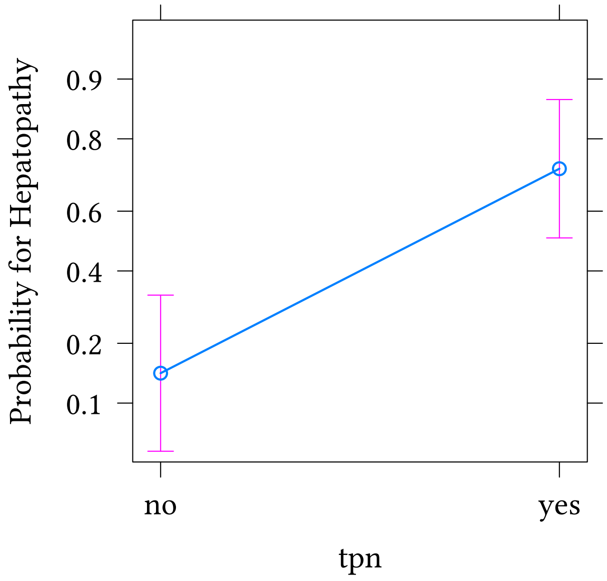

Abbildung 57: Wahrscheinlichkeit für Hepatopathy
(tpn.pdf, tpn.png)

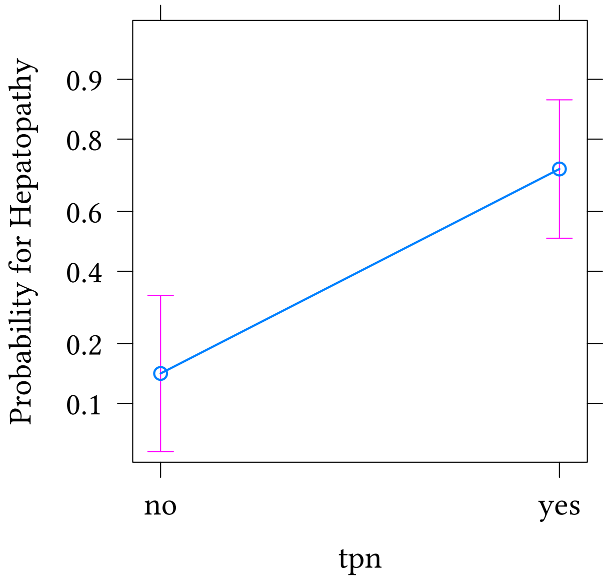

Abbildung 58: Wahrscheinlichkeit für Hepatopathy
(tpn.pdf, tpn.png)

1.50 lh.dysfct

```
Summary: lh.dysfct
$`0`
  n mean   sd median 25% 75% min max range
 30 1.07 0.87      1   1   1   0   3     3

$`1`
  n mean  sd median 25% 75% min max range
 20  1.8 0.7      2   1   2   1   3     2
```

```
Logistische Regression: lh.dysfct
               OR 2.5 % 97.5 % p-value
(Intercept) 0.14  0.03   0.47   0.003
lh.dysfct   3.01  1.44   7.35   0.007
```

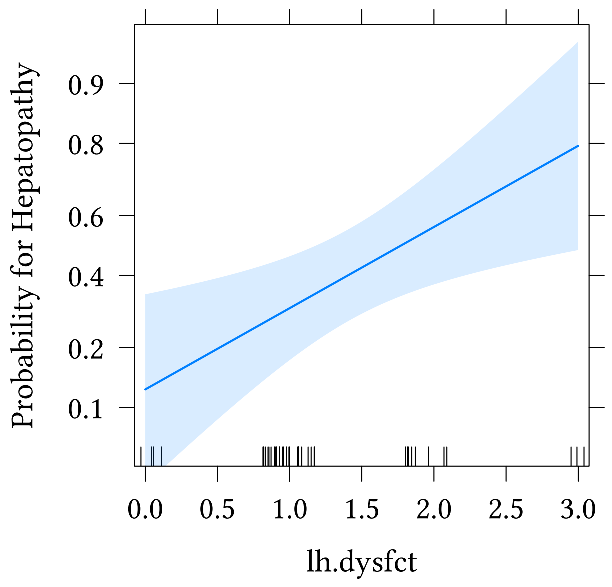

Abbildung 59: Wahrscheinlichkeit für Hepatopathy (`lh.dysfct.pdf`, `lh.dysfct.png`)

1.51 hypotens

```
Summary: hypotens
$`0`
  n mean   sd median 25% 75% min max range
 30  0.9 0.61      1   1   1   0   2     2

$`1`
  n mean   sd median 25% 75% min max range
 20 1.85 0.67      2   1   2   1   3     2
```

```
Logistische Regression: hypotens
               OR 2.5 % 97.5 % p-value
(Intercept)  0.03  0.00   0.15  <0.001
hypotens    10.89  3.41  47.78  <0.001
```

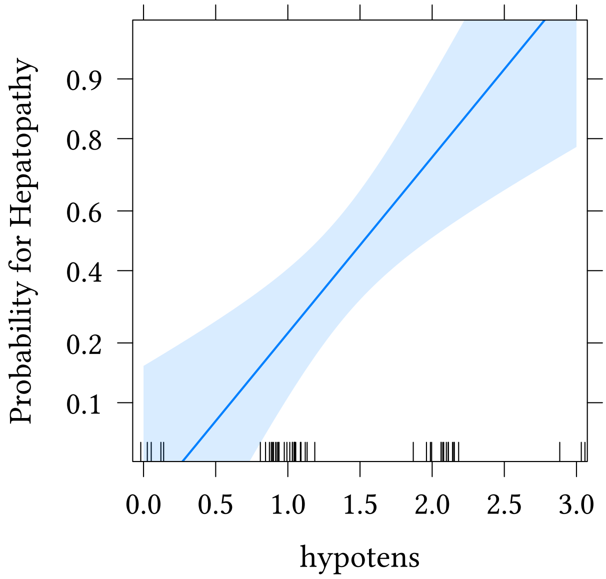

Abbildung 60: Wahrscheinlichkeit für Hepatopathy
(hypotens.pdf, hypotens.png)

2 Multivariates Modell

Das finale multivariate Modell ist:

```
glm(formula = hepatopathy ~ unsched.reop + rh.dysfct + lakt.max +
    sts.eacts + ageM + sex, family = "binomial", data = Dat)
```

Die ORs, Konfidenzintervalle der ORs und p-Werte sind; vgl. auch Abbildung 61 auf der nächsten Seite:

```
                     OR 2.5 %  97.5 % p-value
(Intercept)           0  0.00    0.00   0.015
unsched.reop: yes 86.14  3.44 8691.76   0.020
rh.dysfct          8.98  1.77   99.33   0.027
lakt.max           2.25  1.41    4.74   0.006
sts.eacts          1.61  0.24   12.66   0.616
ageM               2.27  1.41    4.87   0.006
sex: male          0.37  0.02    4.92   0.435
```

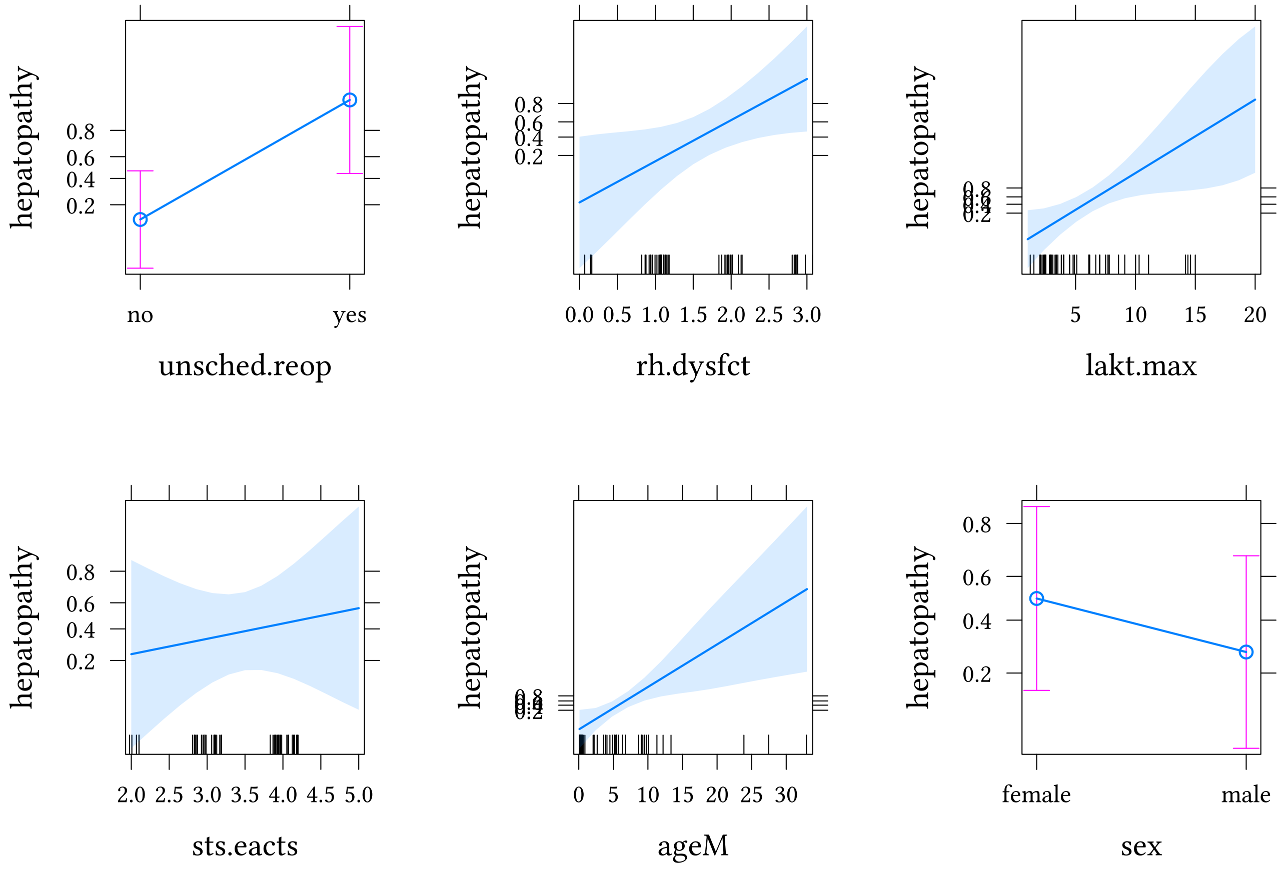

Abbildung 61: Effektplot des finalen Modells; alle unabhängigen Variablen (EffMod1a.pdf, EffMod1a.png)

3 Time to event

3.1 BNP

Das summary der Zeit bis zur Normalisierung ist:

```
  n mean   sd median  25%  75%  min   max range missing
 49 4.76 7.47   1.38 0.23 5.88 0.03 33.87 33.84       1
```

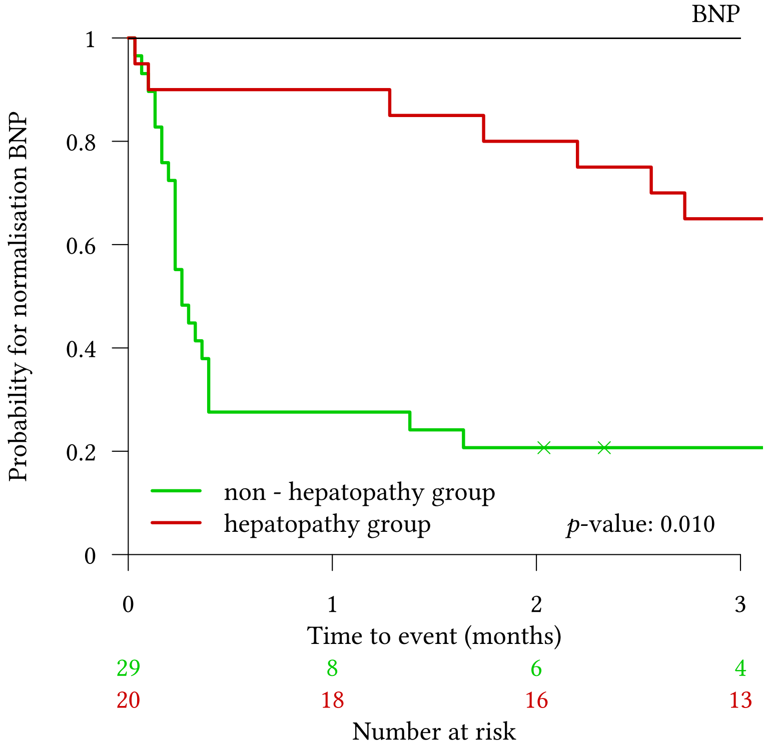

Abbildung 62: Wahrscheinlichkeit für das Erreichen der Normalisierung von BNP (KMbnp.pdf, KMbnp.png)

3.2 TNT

Das summary der Zeit bis zur Normalisierung ist:

```
 n mean   sd median  25% 75%  min   max range
50 1.59 2.64   0.31 0.13 1.7 0.03 11.66 11.63
```

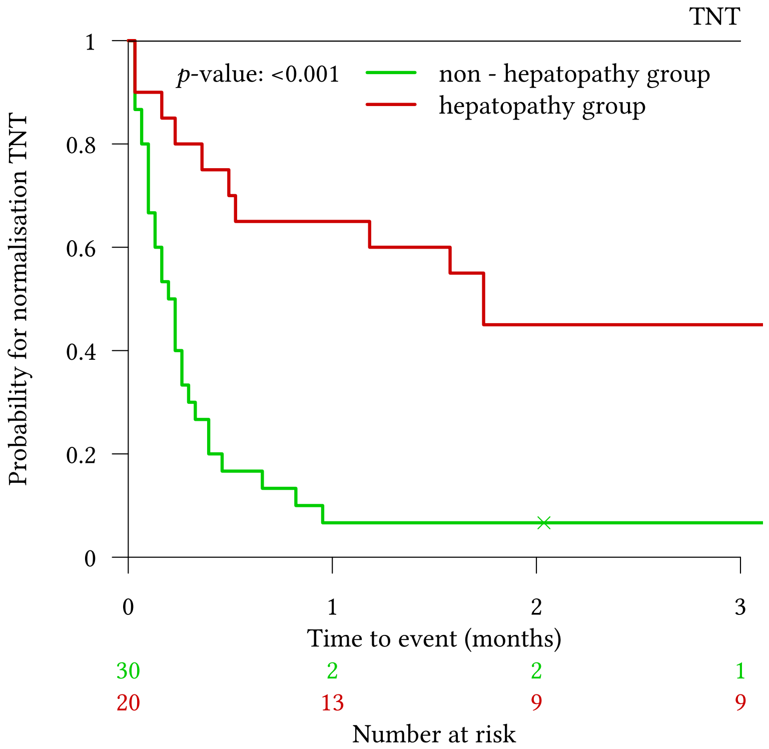

Abbildung 63: Wahrscheinlichkeit für das Erreichen der Normalisierung von TNT (KMtnt.pdf, KMtnt.png)

3.3 GLDH

Das summary der Zeit bis zur Normalisierung ist:

```
 n mean   sd median  25%  75% min  max range missing
49 4.25 9.44   0.26 0.13 2.66   0 47.7  47.7       1
```

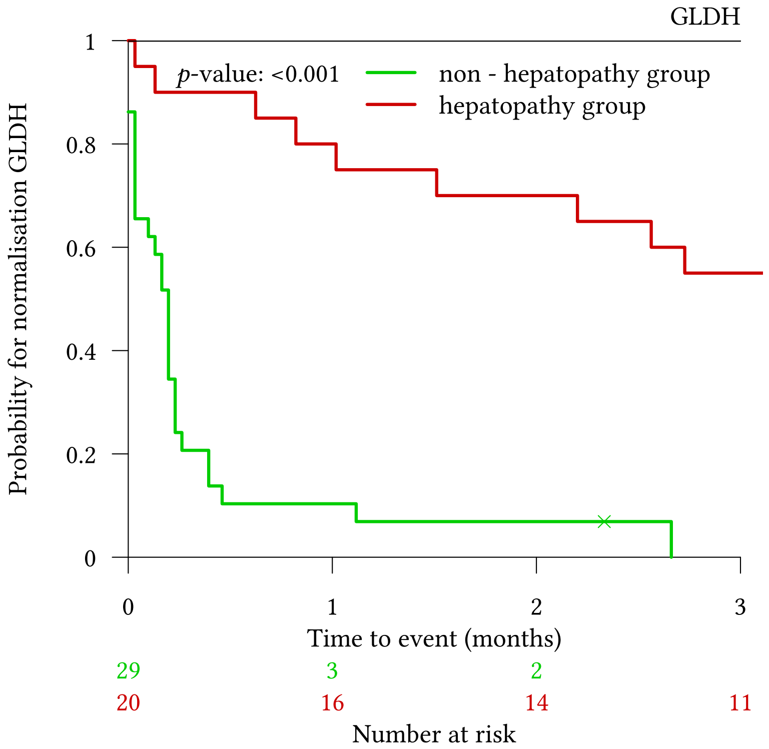

Abbildung 64: Wahrscheinlichkeit für das Erreichen der Normalisierung von GLDH (KMgldh.pdf, KMgldh.png)

3.4 Prothrombin time

Das summary der Zeit bis zur Normalisierung ist:

```
 n mean   sd median  25%  75%  min   max range
50 1.55 3.15    0.2 0.11 1.06 0.03 14.36 14.32
```

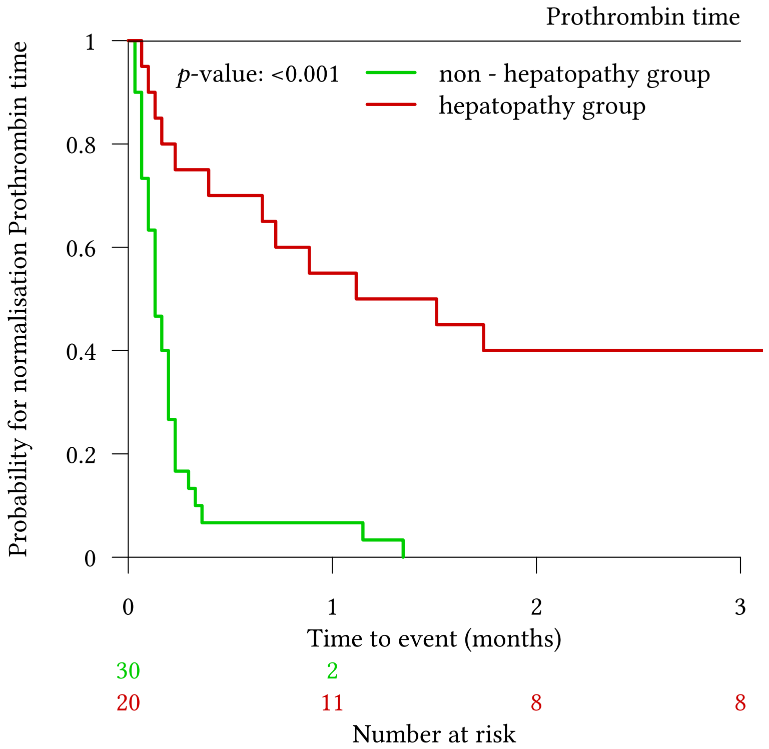

Abbildung 65: Wahrscheinlichkeit für das Erreichen der Normalisierung von QUICK (Prothrombin time) (KMquick.pdf, KMquick.png)

3.5 ALB

Das summary der Zeit bis zur Normalisierung ist:

```
 n mean    sd median  25%   75%  min  max range missing
40  7.1 10.98   1.41 0.25 10.92 0.03 47.7 47.67      10
```

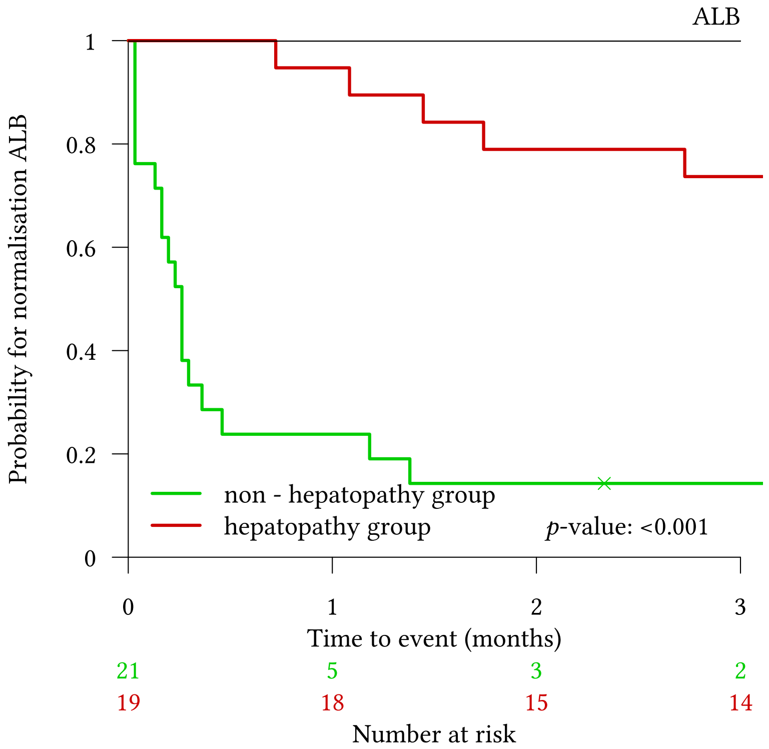

Abbildung 66: Wahrscheinlichkeit für das Erreichen der Normalisierung von ALB (KMalb.pdf, KMalb.png)

3.6 BILI

Das summary der Zeit bis zur Normalisierung ist:

```
 n mean   sd median  25%  75% min   max range
50 2.84 5.58    0.1 0.03 2.12   0 23.33 23.33
```

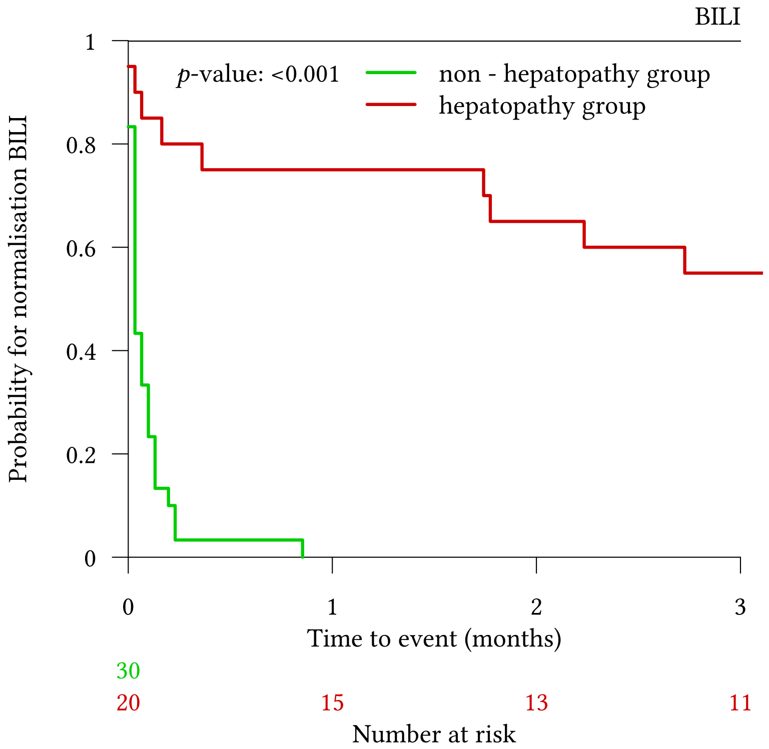

Abbildung 67: Wahrscheinlichkeit für das Erreichen der Normalisierung von BILI (KMbili.pdf, KMbili.png)

3.7 GOT

Das summary der Zeit bis zur Normalisierung ist:

```
 n mean    sd median  25% 75% min  max range
50  5.3 10.02   0.21 0.07 5.4   0 47.7  47.7
```

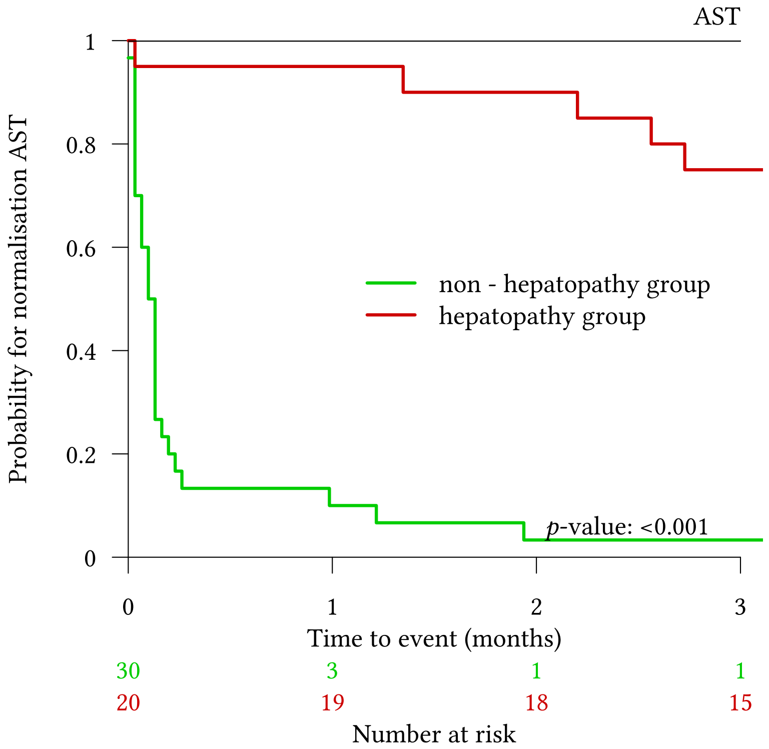

Abbildung 68: Wahrscheinlichkeit für das Erreichen der Normalisierung von GOT (KMgot.pdf, KMgot.png)

3.8 GPT

Das summary der Zeit bis zur Normalisierung ist:

```
 n mean    sd median  25%  75% min  max range
50 5.54 10.12   0.28 0.03 7.95   0 47.7  47.7
```

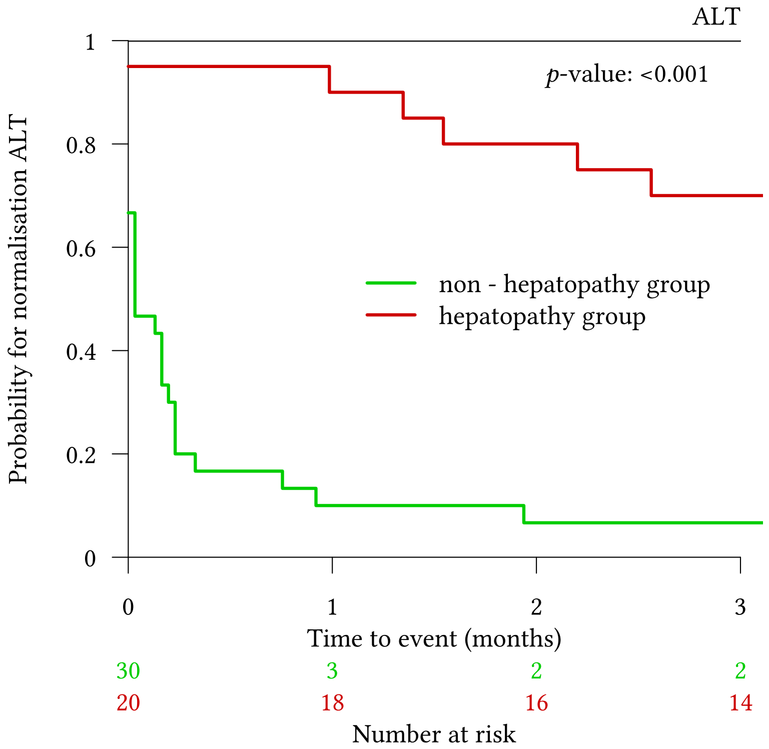

Abbildung 69: Wahrscheinlichkeit für das Erreichen der Normalisierung von GPT (KMgpt.pdf, KMgpt.png)

3.9 GGT

Das summary der Zeit bis zur Normalisierung ist:

```
 n mean    sd median  25%  75% min  max range missing
48 5.66 10.31   0.33 0.03 6.57   0 47.7  47.7       2
```

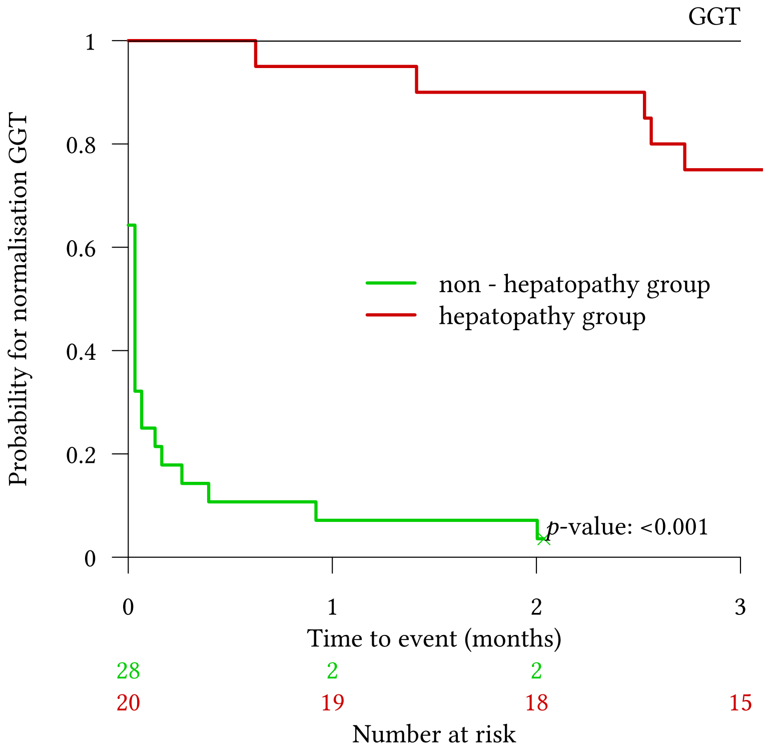

Abbildung 70: Wahrscheinlichkeit für das Erreichen der Normalisierung von GGT (KMggt.pdf, KMggt.png)

3.10 INR

Das summary der Zeit bis zur Normalisierung ist:

```
 n mean   sd median  25%  75%  min   max range
50 1.59 3.15   0.21 0.11 1.14 0.03 14.36 14.32
```

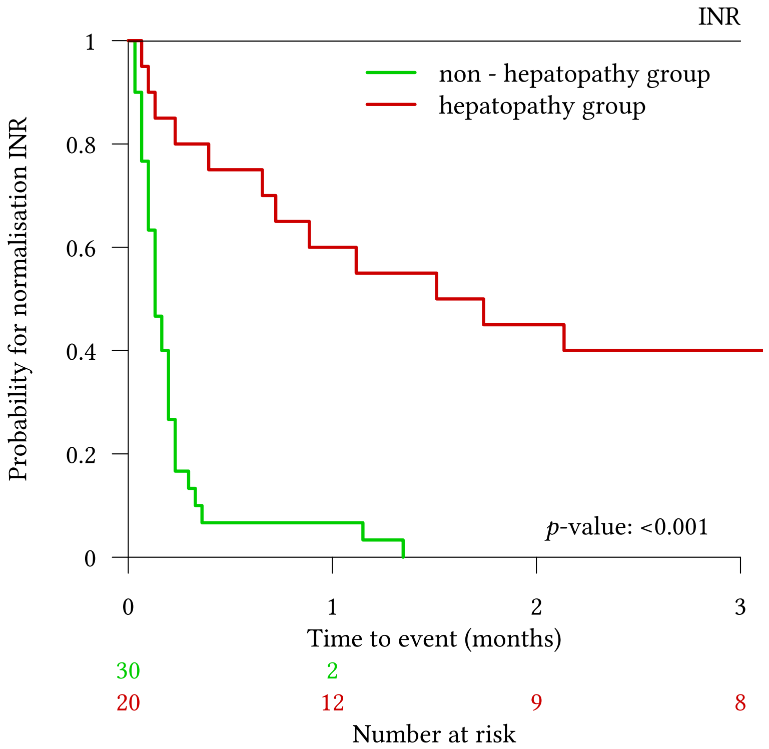

Abbildung 71: Wahrscheinlichkeit für das Erreichen der Normalisierung von INR (KMinr.pdf, KMinr.png)

3.11 KREA

Das summary der Zeit bis zur Normalisierung ist:

```
 n mean   sd median  25%  75%  min   max range
50 0.97 2.54   0.23 0.13 0.52 0.03 12.22 12.19
```

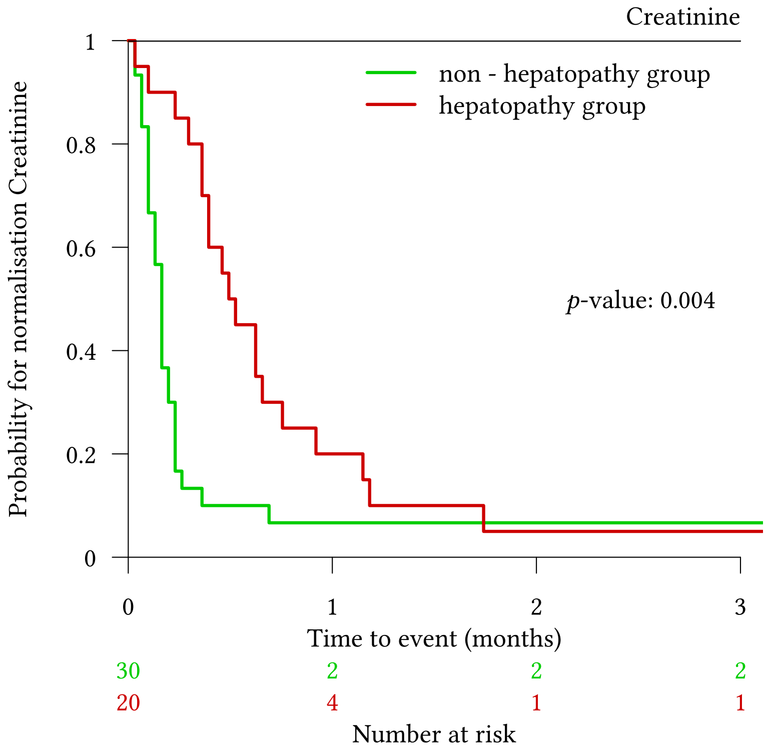

Abbildung 72: Wahrscheinlichkeit für das Erreichen der Normalisierung von Kreatinin (KMkrea.pdf, KMkrea.png)

4 p-Werte für table 1

Die Gruppierungsvariable ist die 2-stufige Variable „hepatopathy“ mit den Ausprägungen „no“ und „yes“.

	valid n	valid %
no	30	60.0
yes	20	40.0
Sum	50	100.0

Für metrische Variablen wird das arithmetische Mittel und die Standardabweichung berichtet und der p-Wert des t-Tests angegeben.

Für kategoriale Variablen werden Häufigkeiten und Prozente bereichtet und der p-Wert des chi²-Tests angegeben.

Das Alter wird in Monaten angegeben.

4.1 age

```
valid cases: 50, missing cases: 0
                        total               no            yes
age: mean (sd)     5.48 (6.96)    3.79 (3.87)    8.03 (9.52)
p-Wert des t-Tests für unabhängige Stichproben: 0.033
```

4.2 sex

```
valid cases: 50, missing cases: 0
           total         no       yes
female 20 (40.0)  7 (23.3) 13 (65.0)
male   30 (60.0) 23 (76.7)  7 (35.0)
p-Wert des chi²-Tests: 0.003
```

4.3 syndr.assc

```
valid cases: 50, missing cases: 0
                      total         no       yes
kein Syndrom      41 (82.0) 26 (86.7) 15 (75.0)
Syndrom vorhanden  9 (18.0)  4 (13.3)  5 (25.0)
p-Wert des chi²-Tests: 0.293
```

4.4 sts.eacts

```
valid cases: 50, missing cases: 0
                                   total                  no
sts.eacts: mean (sd)          3.24 (0.77)          3.13 (0.63)
                 yes
         3.40 (0.94)
p-Wert des t-Tests für unabhängige Stichproben: 0.235
```

4.5 time.cpb

```
valid cases: 50, missing cases: 0
                                  total                    no                  yes
time.cpb: mean (sd)          3.96 (1.58)          3.37 (0.98)          4.85 (1.90)
p-Wert des t-Tests für unabhängige Stichproben: <0.001
```

4.6 time.act

```
valid cases: 50, missing cases: 0
                                  total                    no                  yes
time.act: mean (sd)          2.05 (0.79)          1.90 (0.64)          2.28 (0.95)
p-Wert des t-Tests für unabhängige Stichproben: 0.090
```

4.7 ecmo

```
valid cases: 50, missing cases: 0
        total         no        yes
no   39 (78.0) 26 (86.7) 13 (65.0)
yes 11 (22.0)  4 (13.3)  7 (35.0)
p-Wert des chi²-Tests: 0.070
```

4.8 dialy

```
valid cases: 50, missing cases: 0
      total         no        yes
0 43 (86.0) 27 (90.0) 16 (80.0)
1  7 (14.0)  3 (10.0)  4 (20.0)
p-Wert des chi²-Tests: 0.318
```

4.9 secTC

```
valid cases: 50, missing cases: 0
      total         no        yes
0 20 (40.0) 15 (50.0)  5 (25.0)
1 30 (60.0) 15 (50.0) 15 (75.0)
p-Wert des chi²-Tests: 0.077
```

4.10 transf.ekz

```
valid cases: 49, missing cases: 1 (no=0, yes=1)
                                      total                    no
transf.ekz: mean (sd)       597.35 (425.85)       485.17 (321.20)
                  yes
      774.47 (513.12)
p-Wert des t-Tests für unabhängige Stichproben: 0.019
```

4.11 transf.ffp

```
valid cases: 49, missing cases: 1 (no=0, yes=1)
                                          total                       no
transf.ffp: mean (sd)       580.31 (350.19)       536.67 (281.77)
                  yes
      649.21 (436.95)
p-Wert des t-Tests für unabhängige Stichproben: 0.278
```

4.12 transf.tkz

```
valid cases: 49, missing cases: 1 (no=0, yes=1)
                                          total                       no
transf.tkz: mean (sd)       128.14 (175.88)       104.80 (135.66)
                  yes
      165.00 (224.72)
p-Wert des t-Tests für unabhängige Stichproben: 0.247
```

4.13 rh.dysfct

```
valid cases: 50, missing cases: 0
                                        total                     no
rh.dysfct: mean (sd)           1.50 (0.93)           1.07 (0.74)
                 yes
         2.15 (0.81)
p-Wert des t-Tests für unabhängige Stichproben: <0.001
```

4.14 zvs.min

```
valid cases: 50, missing cases: 0
                                    total                  no                 yes
zvs.min: mean (sd)       51.54 (12.40)       55.75 (12.37)       45.22 (9.65)
p-Wert des t-Tests für unabhängige Stichproben: 0.002
```

4.15 lakt.max

```
valid cases: 50, missing cases: 0
                                      total                    no                 yes
lakt.max: mean (sd)           5.52 (3.67)         4.51 (3.08)         7.04 (4.03)
p-Wert des t-Tests für unabhängige Stichproben: 0.015
```

4.16 proc.rel.fact.ple

```
valid cases: 50, missing cases: 0
      total         no       yes
0 29 (58.0) 23 (76.7)  6 (30.0)
1 21 (42.0)  7 (23.3) 14 (70.0)
p-Wert des chi²-Tests: 0.001
```

4.17 proc.rel.fact.cyt

```
valid cases: 50, missing cases: 0
      total        no       yes
0 41 (82.0) 26 (86.7) 15 (75.0)
1  9 (18.0)  4 (13.3)  5 (25.0)
p-Wert des chi²-Tests: 0.293
```

4.18 proc.rel.fact.diap

```
valid cases: 50, missing cases: 0
      total        no       yes
0 44 (88.0) 28 (93.3) 16 (80.0)
1  6 (12.0)   2 (6.7)  4 (20.0)
p-Wert des chi²-Tests: 0.155
```

4.19 proc.rel.fact.throm

```
valid cases: 50, missing cases: 0
      total        no       yes
0 45 (90.0) 26 (86.7) 19 (95.0)
1  5 (10.0)  4 (13.3)   1 (5.0)
p-Wert des chi²-Tests: 0.336
```

4.20 proc.rel.fact.hrs

```
valid cases: 50, missing cases: 0
      total        no       yes
0 23 (46.0) 19 (63.3)  4 (20.0)
1 27 (54.0) 11 (36.7) 16 (80.0)
p-Wert des chi²-Tests: 0.003
```

4.21 unsched.reop

```
valid cases: 50, missing cases: 0
        total        no       yes
no  35 (70.0) 26 (86.7)  9 (45.0)
yes 15 (30.0)  4 (13.3) 11 (55.0)
p-Wert des chi²-Tests: 0.002
```

4.22 infect

```
valid cases: 50, missing cases: 0
        total        no       yes
no  27 (54.0) 22 (73.3)  5 (25.0)
yes 23 (46.0)  8 (26.7) 15 (75.0)
p-Wert des chi²-Tests: <0.001
```

4.23 tpn

```
valid cases: 50, missing cases: 0
      total        no       yes
0 28 (56.0) 24 (80.0)  4 (20.0)
1 22 (44.0)  6 (20.0) 16 (80.0)
p-Wert des chi²-Tests: <0.001
```

4.24 vent.duration

```
valid cases: 50, missing cases: 0
                                            total                         no
vent.duration: mean (sd)            17.57 (25.47)             8.55 (11.61)
                         yes
          31.10 (33.85)
p-Wert des t-Tests für unabhängige Stichproben: 0.001
```

4.25 hosp.time

```
valid cases: 48, missing cases: 2 (no=1, yes=1)
                                    total                  no
hosp.time: mean (sd)        59.25 (62.76)        29.90 (29.10)
                     yes
      104.05 (73.89)
p-Wert des t-Tests für unabhängige Stichproben: <0.001
```

4.26 death

```
valid cases: 50, missing cases: 0
      total        no       yes
0 40 (80.0) 29 (96.7) 11 (55.0)
1 10 (20.0)   1 (3.3)  9 (45.0)
p-Wert des chi²-Tests: <0.001
```

4.27 bnp.max

```
valid cases: 50, missing cases: 0
                                      total                       no
   bnp.max: mean (sd)   68840.28 (192341.96)   32555.67 (29232.49)
                    yes
123267.20 (298174.40)
p-Wert des t-Tests für unabhängige Stichproben: 0.103
```

4.28 tnt.max

```
valid cases: 50, missing cases: 0
                              total                no                 yes
tnt.max: mean (sd)  6057.50 (3349.54)  5757.63 (3315.29)  6507.30 (3435.60)
p-Wert des t-Tests für unabhängige Stichproben: 0.444
```

4.29 gldh.max

```
valid cases: 50, missing cases: 0
                                  total                  no                 yes
gldh.max: mean (sd)     151.59 (211.50)      98.89 (214.34)     230.64 (185.14)
p-Wert des t-Tests für unabhängige Stichproben: 0.029
```

4.30 quick.min

```
valid cases: 50, missing cases: 0
                                    total                   no
quick.min: mean (sd)        44.17 (21.79)        51.66 (20.06)
                 yes
       32.95 (19.73)
p-Wert des t-Tests für unabhängige Stichproben: 0.002
```

4.31 alb.min

```
valid cases: 39, missing cases: 11 (no=9, yes=2)
                                total                  no                 yes
alb.min: mean (sd)       27.39 (6.60)       31.05 (6.16)       23.12 (4.12)
p-Wert des t-Tests für unabhängige Stichproben: <0.001
```

4.32 bili.max

```
valid cases: 50, missing cases: 0
                                  total                  no                 yes
bili.max: mean (sd)         4.59 (5.32)         2.44 (2.46)         7.80 (6.76)
p-Wert des t-Tests für unabhängige Stichproben: <0.001
```

4.33 got.max

```
valid cases: 50, missing cases: 0
                                total                  no                 yes
got.max: mean (sd)    454.02 (648.53)    355.20 (669.97)    602.25 (600.87)
p-Wert des t-Tests für unabhängige Stichproben: 0.190
```

4.34 gpt.max

```
valid cases: 50, missing cases: 0
                                total                  no                 yes
gpt.max: mean (sd)    262.16 (463.64)    153.20 (361.40)    425.60 (555.11)
p-Wert des t-Tests für unabhängige Stichproben: 0.041
```

4.35 ggt.max

```
valid cases: 47, missing cases: 3 (no=3, yes=0)
                                total                  no                 yes
ggt.max: mean (sd)   957.98 (1390.23)     97.11 (168.89)  2120.15 (1471.52)
p-Wert des t-Tests für unabhängige Stichproben: <0.001
```

4.36 proc.rel.fact

```
valid cases: 50, missing cases: 0
                                            total                           no
proc.rel.fact: mean (sd)              0.76 (0.43)                  0.60 (0.50)
                      yes
              1.00 (0.00)
p-Wert des t-Tests für unabhängige Stichproben: <0.001
```

4.37 proc.rel.fact2

```
valid cases: 50, missing cases: 0
        total        no        yes
no  12 (24.0) 12 (40.0)    0 (0.0)
yes 38 (76.0) 18 (60.0) 20 (100.0)
p-Wert des chi²-Tests: 0.001
```

4.38 use.nor

```
valid cases: 50, missing cases: 0
      total        no       yes
0 39 (78.0) 25 (83.3) 14 (70.0)
1 11 (22.0)  5 (16.7)  6 (30.0)
p-Wert des chi²-Tests: 0.265
```

4.39 use.supra

```
valid cases: 50, missing cases: 0
      total        no       yes
0  6 (12.0)  5 (16.7)   1 (5.0)
1 44 (88.0) 25 (83.3) 19 (95.0)
p-Wert des chi²-Tests: 0.214
```

4.40 use.milr

```
valid cases: 50, missing cases: 0
      total        no       yes
0   2 (4.0)   1 (3.3)   1 (5.0)
1 48 (96.0) 29 (96.7) 19 (95.0)
p-Wert des chi²-Tests: 0.768
```

4.41 lh.dysfct

```
valid cases: 50, missing cases: 0
                                    total                  no
lh.dysfct: mean (sd)          1.36 (0.88)         1.07 (0.87)
                  yes
          1.80 (0.70)
p-Wert des t-Tests für unabhängige Stichproben: 0.003
```

4.42 hypotens

```
valid cases: 50, missing cases: 0
                                  total                  no                 yes
hypotens: mean (sd)          1.28 (0.78)         0.90 (0.61)         1.85 (0.67)
p-Wert des t-Tests für unabhängige Stichproben: <0.001
```

4.43 inr.prop

```
valid cases: 50, missing cases: 0
                                  total                  no                 yes
inr.prop: mean (sd)          1.10 (0.14)         1.11 (0.17)         1.08 (0.08)
p-Wert des t-Tests für unabhängige Stichproben: 0.457
```

4.44 inr.max

```
valid cases: 50, missing cases: 0
                                total                  no                 yes
inr.max: mean (sd)         2.07 (1.09)         1.61 (0.46)         2.77 (1.38)
p-Wert des t-Tests für unabhängige Stichproben: <0.001
```

4.45 krea.prop

```
valid cases: 50, missing cases: 0
                                    total                  no
krea.prop: mean (sd)           0.37 (0.14)         0.37 (0.13)
                  yes
         0.38 (0.14)
p-Wert des t-Tests für unabhängige Stichproben: 0.816
```

4.46 krea.postop

```
valid cases: 50, missing cases: 0
                                        total                  no
krea.postop: mean (sd)            0.95 (0.45)         0.74 (0.28)
                    yes
           1.27 (0.47)
p-Wert des t-Tests für unabhängige Stichproben: <0.001
```

4.47 bnp.min

```
valid cases: 50, missing cases: 0
                                total                  no                 yes
bnp.min: mean (sd) 8328.64 (10253.38) 7884.07 (10432.27) 8995.50 (10209.65)
p-Wert des t-Tests für unabhängige Stichproben: 0.711
```

4.48 tnt.min

```
valid cases: 48, missing cases: 2 (no=1, yes=1)
                                total                  no                 yes
```

```
tnt.min: mean (sd)      46.12 (45.90)      50.69 (52.96)      39.16 (32.45)
p-Wert des t-Tests für unabhängige Stichproben: 0.400
```

4.49 gldh.min

```
valid cases: 44, missing cases: 6 (no=6, yes=0)
                                 total                      no                 yes
gldh.min: mean (sd)         9.47 (7.20)        10.45 (8.51)         8.29 (5.19)
p-Wert des t-Tests für unabhängige Stichproben: 0.325
```

4.50 alb.prop

```
valid cases: 39, missing cases: 11 (no=6, yes=5)
                                 total                      no                 yes
alb.prop: mean (sd)        33.95 (5.32)        32.26 (5.61)        36.66 (3.54)
p-Wert des t-Tests für unabhängige Stichproben: 0.010
```

4.51 bili.prop

```
valid cases: 49, missing cases: 1 (no=0, yes=1)
                                   total                      no
bili.prop: mean (sd)          1.50 (2.23)          1.65 (2.39)
                  yes
         1.26 (2.01)
p-Wert des t-Tests für unabhängige Stichproben: 0.557
```

4.52 got.prop

```
valid cases: 50, missing cases: 0
                                 total                      no                 yes
got.prop: mean (sd)       44.58 (24.07)       47.00 (28.27)       40.95 (15.86)
p-Wert des t-Tests für unabhängige Stichproben: 0.390
```

4.53 gpt.prop

```
valid cases: 50, missing cases: 0
                                 total                      no                 yes
gpt.prop: mean (sd)       27.46 (15.46)       28.53 (14.69)       25.85 (16.80)
p-Wert des t-Tests für unabhängige Stichproben: 0.553
```

4.54 ggt.prop

```
valid cases: 43, missing cases: 7 (no=4, yes=3)
                                 total                      no                 yes
ggt.prop: mean (sd)      58.95 (106.96)       34.08 (27.71)      97.00 (162.14)
p-Wert des t-Tests für unabhängige Stichproben: 0.058
```

Literatur

[1] R Core Team. *R: A Language and Environment for Statistical Computing*. R Foundation for Statistical Computing. Vienna, Austria, 2020. URL: https://www.r-project.org/.

Abbildungsverzeichnis

1 Wahrscheinlichkeit für Hepatopathy (sex.pdf, sex.png) 4
2 Wahrscheinlichkeit für Hepatopathy (age.pdf, age.png) 5
3 Wahrscheinlichkeit für Hepatopathy (cat.chd.pdf, cat.chd.png) 6
4 Wahrscheinlichkeit für Hepatopathy (sts.eacts.pdf, sts.eacts.png) 7
5 Wahrscheinlichkeit für Hepatopathy (unsched.reop.pdf, unsched.reop.png) 8
6 Wahrscheinlichkeit für Hepatopathy (hc.pre.op.pdf, hc.pre.op.png) 9
7 Wahrscheinlichkeit für Hepatopathy (time.cpb.pdf, time.cpb.png) 10
8 Wahrscheinlichkeit für Hepatopathy (time.act.pdf, time.act.png) 11
9 Wahrscheinlichkeit für Hepatopathy (time.act.pdf, time.act.png) 12
10 Wahrscheinlichkeit für Hepatopathy (ecmo.pdf, ecmo.png) 13
11 Wahrscheinlichkeit für Hepatopathy (ecmo.dur.pdf, ecmo.dur.png) 14
12 Wahrscheinlichkeit für Hepatopathy (secTC.pdf, secTC.png) 15
13 Wahrscheinlichkeit für Hepatopathy (rh.dysfct.pdf, rh.dysfct.png) 16
14 Wahrscheinlichkeit für Hepatopathy (vent.duration.pdf, vent.duration.png) 17
15 Wahrscheinlichkeit für Hepatopathy (vent.before.op.pdf, vent.before.op.png) 18
16 Wahrscheinlichkeit für Hepatopathy (dialy.pdf, dialy.png) 19
17 Wahrscheinlichkeit für Hepatopathy (dialy.dur.pdf, dialy.dur.png) 20
18 Wahrscheinlichkeit für Hepatopathy (hosp.time.pdf, hosp.time.png) 21

19 Wahrscheinlichkeit für Hepatopathy
(transf.ekz.pdf, transf.ekz.png) 22
20 Wahrscheinlichkeit für Hepatopathy
(transf.ekz.log.pdf, transf.ekz.log.png) 23
21 Wahrscheinlichkeit für Hepatopathy
(transf.ffp.pdf, transf.ffp.png) 24
22 Wahrscheinlichkeit für Hepatopathy
(transf.ffp.log.pdf, transf.ffp.log.png) 25
23 Wahrscheinlichkeit für Hepatopathy
(transf.tkz.pdf, transf.tkz.png) 26
24 Wahrscheinlichkeit für Hepatopathy
(transf.tkz.log.pdf, transf.tkz.log.png) 27
25 Wahrscheinlichkeit für Hepatopathy
(mix.zirk.pdf, mix.zirk.png) 28
26 Wahrscheinlichkeit für Hepatopathy
(zvs.min.pdf, zvs.min.png) 29
27 Wahrscheinlichkeit für Hepatopathy
(lakt.max.pdf, lakt.max.png) 30
28 Wahrscheinlichkeit für Hepatopathy
(lak.norm.pdf, lak.norm.png) 31
29 Wahrscheinlichkeit für Hepatopathy
(infect.pdf, infect.png) 32
30 Wahrscheinlichkeit für Hepatopathy
(bnp.min.pdf, bnp.min.png) 33
31 Wahrscheinlichkeit für Hepatopathy
(bnp.max.pdf, bnp.max.png) 34
32 Wahrscheinlichkeit für Hepatopathy
(tnt.min.pdf, tnt.min.png) 35
33 Wahrscheinlichkeit für Hepatopathy
(tnt.max.pdf, tnt.max.png) 36
34 Wahrscheinlichkeit für Hepatopathy
(gldh.min.pdf, gldh.min.png) 37
35 Wahrscheinlichkeit für Hepatopathy
(gldh.max.pdf, gldh.max.png) 38
36 Wahrscheinlichkeit für Hepatopathy
(quick.min.pdf, quick.min.png) 39
37 Wahrscheinlichkeit für Hepatopathy
(alb.min.pdf, alb.min.png) 40
38 Wahrscheinlichkeit für Hepatopathy
(bili.max.pdf, bili.max.png) 41
39 Wahrscheinlichkeit für Hepatopathy
(got.max.pdf, got.max.png) 42
40 Wahrscheinlichkeit für Hepatopathy
(gpt.max.pdf, gpt.max.png) 43

41 Wahrscheinlichkeit für Hepatopathy
(ggt.max.pdf, ggt.max.png) 44
42 Wahrscheinlichkeit für Hepatopathy
(proc.rel.fact.ple.pdf, proc.rel.fact.ple.png) 46
43 Wahrscheinlichkeit für Hepatopathy
(proc.rel.fact.cyt.pdf, proc.rel.fact.cyt.png) 47
44 Wahrscheinlichkeit für Hepatopathy
(proc.rel.fact.cyt.pdf, proc.rel.fact.cyt.png) 48
45 Wahrscheinlichkeit für Hepatopathy
(proc.rel.fact.diap.pdf, proc.rel.fact.diap.png) 49
46 Wahrscheinlichkeit für Hepatopathy
(proc.rel.fact.diap.pdf, proc.rel.fact.diap.png) 50
47 Wahrscheinlichkeit für Hepatopathy
(proc.rel.fact.throm.pdf, proc.rel.fact.throm.png) 51
48 Wahrscheinlichkeit für Hepatopathy
(proc.rel.fact.throm.pdf, proc.rel.fact.throm.png) 52
49 Wahrscheinlichkeit für Hepatopathy
(proc.rel.fact.hrs.pdf, proc.rel.fact.hrs.png) 53
50 Wahrscheinlichkeit für Hepatopathy
(proc.rel.fact.hrs.pdf, proc.rel.fact.hrs.png) 54
51 Wahrscheinlichkeit für Hepatopathy
(use.nor.pdf, use.nor.png) 55
52 Wahrscheinlichkeit für Hepatopathy
(use.nor.pdf, use.nor.png) 56
53 Wahrscheinlichkeit für Hepatopathy
(use.supra.pdf, use.supra.png) 57
54 Wahrscheinlichkeit für Hepatopathy
(use.supra.pdf, use.supra.png) 58
55 Wahrscheinlichkeit für Hepatopathy
(use.milr.pdf, use.milr.png) 59
56 Wahrscheinlichkeit für Hepatopathy
(use.milr.pdf, use.milr.png) 60
57 Wahrscheinlichkeit für Hepatopathy
(tpn.pdf, tpn.png) 61
58 Wahrscheinlichkeit für Hepatopathy
(tpn.pdf, tpn.png) 62
59 Wahrscheinlichkeit für Hepatopathy
(lh.dysfct.pdf, lh.dysfct.png) 63
60 Wahrscheinlichkeit für Hepatopathy
(hypotens.pdf, hypotens.png) 64
61 Effektplot des finalen Modells; alle unabhängigen Variablen
(EffMod1a.pdf, EffMod1a.png) 66
62 Wahrscheinlichkeit für das Erreichen der Normalisierung von BNP
(KMbnp.pdf, KMbnp.png) 67

63 Wahrscheinlichkeit für das Erreichen der Normalisierung von TNT
(KMtnt.pdf, KMtnt.png) 68
64 Wahrscheinlichkeit für das Erreichen der Normalisierung von GLDH
(KMgldh.pdf, KMgldh.png) 69
65 Wahrscheinlichkeit für das Erreichen der Normalisierung von QUICK (Prothrombin time)
(KMquick.pdf, KMquick.png) 70
66 Wahrscheinlichkeit für das Erreichen der Normalisierung von ALB
(KMalb.pdf, KMalb.png) 71
67 Wahrscheinlichkeit für das Erreichen der Normalisierung von BILI
(KMbili.pdf, KMbili.png) 72
68 Wahrscheinlichkeit für das Erreichen der Normalisierung von GOT
(KMgot.pdf, KMgot.png) 73
69 Wahrscheinlichkeit für das Erreichen der Normalisierung von GPT
(KMgpt.pdf, KMgpt.png) 74
70 Wahrscheinlichkeit für das Erreichen der Normalisierung von GGT
(KMggt.pdf, KMggt.png) 75
71 Wahrscheinlichkeit für das Erreichen der Normalisierung von INR
(KMinr.pdf, KMinr.png) 76
72 Wahrscheinlichkeit für das Erreichen der Normalisierung von Kreatinin
(KMkrea.pdf, KMkrea.png) 77
