# Supplementary material for: Impact of hepatopathy in pediatric patients after surgery for complex congenital heart disease
Source: PLoS One. 2021 Mar 25;16(3):e0248776. doi: 10.1371/journal.pone.0248776 (PMC7993827; doi:10.1371/journal.pone.0248776)
Supplement: S1 Material — (ZIP) [file pone.0248776.s001.zip › CardiacHepatopathy_analysis_SPSS.pdf]

```
 * Encoding: UTF-8.
* Encoding: .
* Encoding: .
* Encoding: .
* Encoding: .
* Encoding: .
* Encoding: .
* Encoding: .
* Encoding: .
* Encoding: .
  GET DATA
  /TYPE=XLSX
  /FILE='/Users/torbenkehl/@work/Kinderkardiologie/Studien/Hepatopathie nac
h CPB '
  + 'OP/Revision/dataset_Cardiac_heptopathy_anonym_final.xlsx'
  /SHEET=name 'car_hep_data'
  /CELLRANGE=FULL
  /READNAMES=ON
  /ASSUMEDSTRWIDTH=32767.
EXECUTE.
DATASET NAME procedere_associtaed_hepatopathy WINDOW=FRONT.
```

Dataset Name

Notes

Output Created		13-FEB-2021 14:16:37
Comments		
Input	Filter	<none>
	Weight	<none>
	Split File	<none>
	N of Rows in Working Data File	50
Syntax		DATASET NAME procedere_associtaed_hepatopathy WINDOW=FRONT.
Resources	Processor Time	00:00:00,00
	Elapsed Time	00:00:00,00

Warnings

The active dataset will replace the existing dataset named procedere_associtaed_hepatopathy.

```
 VARIABLE LABELS
pat_id 'patient ID'  age_op 'age at op' sex 'sex' numb_op 'numbers of opera
```

```
tion'
hepatopathy 'hepatopathy associated with cardiac procedere'
ltx 'liver transplantation (LTX) following cardiac procederes'
death 'death in follow up' diagn 'diagnosis'
cat_chd 'category of congenital heart disease (related to "Moss and Adams H
eart Disease in Infants, Children and Adolescents")'
asd 'atrial septum defect' vsd 'ventricular septum defect'
syndr_assc 'syndrome / genetic disorder' proc_rel_fact 'procedere related f
actors' opdat 'date of operation'

>Warning # 4461 in column 84.  Text: opdat
>An unknown variable name was specified on the VAR LABELS command.  The nam
e
>and the label will be ignored.
op_techn 'operation procedere' sts_eacts 'STS-EACTS score' op_hybrid 'hybri
d operation ?'
index_op 'Index OP'
proc_relev 'op procedere relevant for statistical analysis?'
unsched_reop 'unscheduled reintervention (re-operation / re-cath)?'
hc_pre_op 'cardiac catheter' time_cpb 'cardiopulmonary bypass time (hh:mm)'
 time_act 'aortic clamp time (hh:mm)' time_reperf 'reperfusion time (hh:mm)
'
ecmo 'ECMO after operation?'
ecmo_dur 'duration of ECMO? (days)' secTC 'secondary chest closure?'
rh_dysfct 'right heart dysfunction after operation?'
lh_dysfct 'left heart dysfunction after operation?'
hypotens 'Hypotension requiring therapy after operation?'
pat_rel_fact 'patient related factors'
proc_rel_fact_ple 'postoperative pleural effusion?'
proc_rel_fact_cyt 'postoperative pleural chylothorax?'
proc_rel_fact_diap 'postoperative diaphragmatic paresis?'
proc_rel_fact_throm 'postoperative thrombosis?'
proc_rel_fact_hrs 'postoperative cardiac arrhythmias'
use_nor 'postoperative use of noradrenalin'
use_supra 'postoperative use of suprarenin'
use_milr  'postoperative use of milrinon'
vent_duration 'duration of ventilation (hh:mm)' vent_before_op 'indication
for mechanical ventilation before operation'
dialy 'dialysis after operation?' dialy_dur 'duration of dialysis (days)'
tpn 'total parental nutrition'
hosp_time 'duration of hospitalisation (days)'
transf_ekz 'tranfusion of red blood cells (ml)' transf_ffp  'tranfusion of
fresh frozen plasma (ml)' transf_tkz  'tranfusion of platelets (ml)'
mix_zirk 'mix circulation before operation' zvs_preop_max 'central venous s
aturtion maximum before operation (%)'
zvs_min 'central venous saturation min. after proced. (%)'
lakt_op 'lactate perioperative (mmol/l)' lakt_max 'lactate maximum (mmol/l)
 after operation' lak_norm 'duration of normalisation of perioperative lact
ate (d)'
infect 'infection after operation?'
abx_amp_sul 'ampicilin/sulbactam' abx_cefotax 'cefotaxim' abx_genta 'gentam
icin' abx_tobra 'tobramycin' abx_vanco 'vancomycin'
abx_mero 'meropenem' abx_teico 'teicoplanin' abx_fluc 'flucloaxcilin' abx_l
```

```
inez 'linezolid' abx_piptaz 'piperacilin/tazobactam'
bnp_min 'BNP minimum (ng/ml)' bnp_max 'BNP maximum (ng/ml)'  bnp_norm 'tren
d to normalisation BNP (d)'
tnt_min 'TNT minimum (ng/ml)' tnt_max 'TNT maximum (ng/ml)'  tnt_norm 'tren
d to normalisation TNT (d)'
gldh_min 'GLDH minimum (U/l)' gldh_max 'GLDH maximum (U/l)' gldh_norm 'tren
d to normalisation GLDH (d)'
inr_prop 'INR preoperative (%)' inr_max 'INR maximum (%)' inr_norm 'trend t
o normalisation INR (d)'
krea_prop 'Creatinin preoperative' krea_postop 'Creatinin maximum ' krea_no
rm 'trend to normalisation Creatinin (d)'
alb_prop 'albumine preoperative (g/dl)' alb_min 'albumine minimum postopera
tive (g/dl)' alb_norm 'trend to normalisation Albumin (d)'
bili_prop  'bilirubine preoperative (g/dl)' bili_max  'bilirubine maximum p
ostoperative (g/dl)' bili_norm 'trend to normalisation Bilirubin (d)'
got_prop 'GOT minimum (U/l)' got_max 'GOT maximum (U/l)' got_norm 'trend to
 normalisation GOT (d)'
gpt_prop 'GPT minimum (U/l)' gpt_max 'GPT maximum (U/l)' gpt_norm 'trend to
 normalisation GPT (d)'
ggt_prop 'gGT minimum (U/l)' ggt_max 'gGT maximum (U/l)'  ggt_norm 'trend t
o normalisation gGT (d)'
liver_us 'ultrasound of the liver after operation?' liv_us_siz 'ultrasound
pathologic enlargement of the liver?' liv_us_paren 'ultrasound pathologic p
arenchym of the liver?' liv_us_perf 'ultrasound pathologic perfusion of the
 liver?'
liver_histo 'liver biopsy after operation?'  liv_histo_chol 'histopathologi
c findings: cholestasis'  liv_histo_fibr 'histopathologic findings: fibrosi
s'
liv_histo_cirr 'histopathologic findings: cirrhosis' liv_histo_necr 'histop
athologic findings: necrosis?'.

FORMATS time_cpb time_act time_reperf (TIME5) /  bnp_norm tnt_norm gldh_nor
m inr_norm krea_norm alb_norm bili_norm got_norm gpt_norm ggt_norm(EDATE10)
.
FORMATS vent_duration (TIME5).
FORMATS lakt_max (F2.1).

MISSING VALUES  zvs_preop_max  zvs_min lakt_op lakt_max lak_norm
    bnp_min bnp_max tnt_min tnt_max
    gldh_min gldh_max alb_prop alb_min bili_prop bili_max got_prop got_max
    gpt_prop gpt_max ggt_prop ggt_max inr_prop inr_max inr_norm krea_prop k
rea_postop krea_norm(-999).

MISSING VALUES  transf_ekz transf_ffp transf_tkz (-999).

MISSING VALUES infect (-999).

MISSING VALUES zvs_preop_max zvs_min lakt_op lakt_max lak_norm (-999).

VALUE LABELS
sex 0 'male' 1 'female'
/hepatopathy 0 'no' 1 'yes'
/ltx 0 'no LTX' 1 'LTX executed' 2'evluation for LTX' 3 'listing for LTX'
```

/death 0 'no' 1 'yes'
/cat_chd 1 'I Septal defects (e.g. VSD, AVSD)' 2 'II Arterial abnormalities (e.g. Aortic arch abnormalities)' 3 'III Venous abnormalities (e.g. TAPVC)'
4 'IV Tricuspid valve abnormalities (e.g. M. Ebstein)' 5 'V Right ventricle and Pulmonary outflow abnormalities (e.g. TOF, PA)' 6 'VI Mitral valve abnormalities'
7 'VII Left ventricle outflow abnormalities (e.g. HLHC/HLHS, Shone complex)' 8 'VIII Abnormalities of the origin of the great Arteries (e.g. dTGA, cTGA, DORV)'
9 'IX Complex Cardiac abnormalities (e.g. DILV, Univentricle)' 10 ' X Cardiomyopathy (e.g. DCM)'
/asd 0 'no ASD' 1 'ASD I' 2 'ASD II' 3 'PFO'
/vsd 0 'no VSD' 1 'VSD' 2 'muscular VSD' 3 'Inlet VSD' 4 'malalignment VSD' 5 'perimembranous VSD' 6 'subpulmonary VSD' 7 'subaortic VSD' 8 'doubly commited VSD'
/index_op 0 'no' 1 'yes'
/pat_rel_fact 0 'not present' 1 'present'
/syndr_assc 0 'not present' 1 'trisomy 21' 2 'DiGeorge syndrome' 3 'CHARGE associtaion' 4 'VACTERL associtaion' 5 'others'
/op_hybrid 0 'no' 1 'yes'
/hc_pre_op 0 'no' 1 'diagnostic cardiac catheter' 2 interventional cardiac catheter'
/proc_relev 0 'no' 1 'yes'
/unsched_reop 0 'no' 1 'yes'
/ecmo 0 'no' 1 'yes'
/secTC 0 'no' 1 'yes'
/rh_dysfct 0 'no right heart dysfunction' 1 'mild right heart dysfunction'
2 'moderate right heart dysfunction' 3 'serve right heart dysfunction'
/lh_dysfct 0 'no left heart dysfunction' 1 'mild left heart dysfunction' 2 'moderate left heart dysfunction' 3 'serve left heart dysfunction'
/hypotens 0 'no hypotension' 1 'mild hypotension' 2 'moderate hypotension'
3 'serve hypotension'
/proc_rel_fact 0 'not present' 1 'present'
/proc_rel_fact_ple 0 'not present' 1 'present'
/proc_rel_fact_cyt 0 'not present' 1 'present'
/proc_rel_fact_diap 0 'not present' 1 'present'
/proc_rel_fact_throm 0 'not present' 1 'present'
/proc_rel_fact_hrs 0 'not present' 1 'junctional ectopic tachycardia' 2 'other tachycardia' 3 'higher grade av block' 4 'transient bradycardia'
/use_nor 0 'no' 1 'yes'
/use_supra 0 'no' 1 'yes'
/use_milr 0 'no' 1 'yes'
/vent_before_op 0 'no' 1 'yes'
/dialy 0 'no' 1 'yes'
/tpn 0 'no' 1 'yes'
/mix_zirk 0 'no' 1 'yes'
/infect 0 'no' 1 'yes' -999 'n.s.'
/liver_us 0 'no' 1 'yes'
/liv_us_siz 0 'no' 1 'liver enlargement' 2 'hepatomegaly' 3 'condition after LTX'
/liv_us_paren 0 'no alterations of the liver parenchym' 1 'milde alterations of the liver parenchym' 2 'moderate alterations of the liver parenchym' 3

```
 'fibrosis' 4 cirrhosis'
/liv_us_perf 0 'no perfusion alterations of the liver parenchym' 1 'moderat
e perfusion alterations of the liver parenchym' 2 'retrograde portal vein f
low'
/liver_histo 0 'no biopsy' 1 'biopsy executed'
/liv_histo_fibr 0 'no' 1 'yes'
/liv_histo_cirr 0 'no' 1 'yes'
/liv_histo_necr 0 'no' 1 'yes'
/abx_amp_sul 0 'no' 1 'yes'
/abx_cefotax 0 'no' 1 'yes'
/abx_genta 0 'no' 1 'yes'
/abx_tobra 0 'no' 1 'yes'
/abx_vanco 0 'no' 1 'yes'
/abx_mero 0 'no' 1 'yes'
/abx_teico 0 'no' 1 'yes'
/abx_fluc 0 'no' 1 'yes'
/abx_linez 0 'no' 1 'yes'
/abx_piptaz 0 'no' 1 'yes'
/transf_ekz transf_ffp transf_tkz zvs_preop_max zvs_min lakt_op lakt_max la
k_norm
    bnp_min bnp_max tnt_min tnt_max
    gldh_min gldh_max inr_prop inr_max krea_prop krea_postop alb_prop alb_m
in bili_prop bili_max got_prop got_max
    gpt_prop gpt_max ggt_prop ggt_max -999 'n.s.'.



RECODE proc_rel_fact_hrs(0=0) (1 thru 5=1) INTO proc_rel_fact_hrs_neu.
VARIABLE LABELS  proc_rel_fact_hrs_neu 'KO HRS'.
EXECUTE.


FREQUENCIES sex.
```

Frequencies

Notes

Output Created		13-FEB-2021 14:16:37
Comments		
Input	Active Dataset	procedere_associtaed_hepatopathy
	Filter	<none>
	Weight	<none>
	Split File	<none>
	N of Rows in Working Data File	50
Missing Value Handling	Definition of Missing	User-defined missing values are treated as missing.
	Cases Used	Statistics are based on all cases with valid data.
Syntax		FREQUENCIES sex.
Resources	Processor Time	00:00:00,00
	Elapsed Time	00:00:00,00

Statistics

sex

N	Valid	50
	Missing	0

sex

		Frequency	Percent	Valid Percent	Cumulative Percent
Valid	male	30	60,0	60,0	60,0
	female	20	40,0	40,0	100,0
	Total	50	100,0	100,0	

```
CROSSTABS
 /TABLES=cat_chd BY hepatopathy
 /FORMAT=AVALUE TABLES
 /STATISTICS=CHISQ
 /CELLS=COUNT COLUMN
 /COUNT ROUND CELL
 /METHOD=EXACT TIMER(5).
```

Crosstabs

Notes

Output Created		13-FEB-2021 14:16:37
Comments		
Input	Active Dataset	procedere_associtaed_hepatopathy
	Filter	<none>
	Weight	<none>
	Split File	<none>
	N of Rows in Working Data File	50
Missing Value Handling	Definition of Missing	User-defined missing values are treated as missing.
	Cases Used	Statistics for each table are based on all the cases with valid data in the specified range(s) for all variables in each table.
Syntax		CROSSTABS<br>/TABLES=cat_chd BY hepatopathy<br>/FORMAT=AVALUE TABLES<br>/STATISTICS=CHISQ<br>/CELLS=COUNT COLUMN<br>/COUNT ROUND CELL<br>/METHOD=EXACT TIMER(5).
Resources	Processor Time	00:00:00,03
	Elapsed Time	00:00:00,00
	Dimensions Requested	2
	Cells Available	524245
	Time for Exact Statistics	0:00:00,01

Case Processing Summary

	Cases					
	Valid		Missing		Total	
	N	Percent	N	Percent	N	Percent
category of congenital heart disease (related to "Moss and Adams Heart Disease in Infants, Children and Adolescents") * hepatopathy associated with cardiac procedere	50	100,0%	0	0,0%	50	100,0%

category of congenital heart disease (related to "Moss and Adams Heart Disease in Infants, Children and Adolescents") * hepatopathy associated with cardiac procedere Crosstabulation

			hepatopathy associated with
			no
category of congenital heart disease (related to "Moss and Adams Heart Disease in Infants, Children and Adolescents")	I Septal defects (e.g. VSD, AVSD)	Count	3
		% within hepatopathy associated with cardiac procedere	10,0%
	II Arterial abnormalities (e.g. Aortic arch abnormalities)	Count	0
		% within hepatopathy associated with cardiac procedere	0,0%
	III Venous abnormalities (e.g. TAPVC)	Count	1
		% within hepatopathy associated with cardiac procedere	3,3%
	V Right ventricle and Pulmonary outflow abnormalities (e.g. TOF, PA)	Count	7
		% within hepatopathy associated with cardiac procedere	23,3%
	VII Left ventricle outflow abnormalities (e.g. HLHC/HLHS, Shone complex)	Count	0
		% within hepatopathy associated with cardiac procedere	0,0%
	VIII Abnormalities of the origin of the great Arteries (e.g. dTGA, cTGA, DORV)	Count	17
		% within hepatopathy associated with cardiac procedere	56,7%
	IX Complex Cardiac abnormalities (e.g. DILV, Univentricle)	Count	2
		% within hepatopathy associated with cardiac procedere	6,7%
	X Cardiomyopathy (e.g. DCM)	Count	0
		% within hepatopathy associated with cardiac procedere	0,0%
Total		Count	30
		% within hepatopathy associated with cardiac procedere	100,0%

category of congenital heart disease (related to "Moss and Adams Heart Disease in Infants, Children and Adolescents") * hepatopathy associated with cardiac procedere Crosstabulation

			hepatopathy associated with...
			yes
category of congenital heart disease (related to "Moss and Adams Heart Disease in Infants, Children and Adolescents")	I Septal defects (e.g. VSD, AVSD)	Count	4
		% within hepatopathy associated with cardiac procedere	20,0%
	II Arterial abnormalities (e.g. Aortic arch abnormalities)	Count	1
		% within hepatopathy associated with cardiac procedere	5,0%
	III Venous abnormalities (e.g. TAPVC)	Count	0
		% within hepatopathy associated with cardiac procedere	0,0%
	V Right ventricle and Pulmonary outflow abnormalities (e.g. TOF, PA)	Count	7
		% within hepatopathy associated with cardiac procedere	35,0%
	VII Left ventricle outflow abnormalities (e.g. HLHC/HLHS, Shone complex)	Count	1
		% within hepatopathy associated with cardiac procedere	5,0%
	VIII Abnormalities of the origin of the great Arteries (e.g. dTGA, cTGA, DORV)	Count	5
		% within hepatopathy associated with cardiac procedere	25,0%
	IX Complex Cardiac abnormalities (e.g. DILV, Univentricle)	Count	1
		% within hepatopathy associated with cardiac procedere	5,0%
	X Cardiomyopathy (e.g. DCM)	Count	1
		% within hepatopathy associated with cardiac procedere	5,0%
Total		Count	20
		% within hepatopathy associated with cardiac procedere	100,0%

category of congenital heart disease (related to "Moss and Adams Heart Disease in Infants, Children and Adolescents") * hepatopathy associated with cardiac procedere Crosstabulation

			Total
category of congenital heart disease (related to "Moss and Adams Heart Disease in Infants, Children and Adolescents")	I Septal defects (e.g. VSD, AVSD)	Count	7
		% within hepatopathy associated with cardiac procedere	14,0%
	II Arterial abnormalities (e.g. Aortic arch abnormalities)	Count	1
		% within hepatopathy associated with cardiac procedere	2,0%
	III Venous abnormalities (e.g. TAPVC)	Count	1
		% within hepatopathy associated with cardiac procedere	2,0%
	V Right ventricle and Pulmonary outflow abnormalities (e.g. TOF, PA)	Count	14
		% within hepatopathy associated with cardiac procedere	28,0%
	VII Left ventricle outflow abnormalities (e.g. HLHC/HLHS, Shone complex)	Count	1
		% within hepatopathy associated with cardiac procedere	2,0%
	VIII Abnormalities of the origin of the great Arteries (e.g. dTGA, cTGA, DORV)	Count	22
		% within hepatopathy associated with cardiac procedere	44,0%
	IX Complex Cardiac abnormalities (e.g. DILV, Univentricle)	Count	3
		% within hepatopathy associated with cardiac procedere	6,0%
	X Cardiomyopathy (e.g. DCM)	Count	1
		% within hepatopathy associated with cardiac procedere	2,0%
Total		Count	50
		% within hepatopathy associated with cardiac procedere	100,0%

Chi-Square Tests

	Value	df	Asymptotic Significance (2-sided)	Exact Sig. (2-sided)	Exact Sig. (1-sided)
Pearson Chi-Square	9,398[a]	7	,225	,178	
Likelihood Ratio	10,931	7	,142	,201	
Fisher's Exact Test	9,283			,137	
Linear-by-Linear Association	2,248[b]	1	,134	,144	,076
N of Valid Cases	50				

Chi-Square Tests

	Point Probability
Pearson Chi-Square	
Likelihood Ratio	
Fisher's Exact Test	
Linear-by-Linear Association	,014
N of Valid Cases	

a. 12 cells (75,0%) have expected count less than 5. The minimum expected count is ,40.

b. The standardized statistic is -1,499.

```
CROSSTABS
  /TABLES=syndr_assc BY hepatopathy
  /FORMAT=AVALUE TABLES
  /STATISTICS=CHISQ
  /CELLS=COUNT COLUMN
  /COUNT ROUND CELL
  /METHOD=EXACT TIMER(5).
```

Crosstabs

Notes

Output Created		13-FEB-2021 14:16:37
Comments		
Input	Active Dataset	procedere_associtaed_hepatopathy
	Filter	<none>
	Weight	<none>
	Split File	<none>
	N of Rows in Working Data File	50
Missing Value Handling	Definition of Missing	User-defined missing values are treated as missing.
	Cases Used	Statistics for each table are based on all the cases with valid data in the specified range(s) for all variables in each table.
Syntax		CROSSTABS /TABLES=syndr_assc BY hepatopathy /FORMAT=AVALUE TABLES /STATISTICS=CHISQ /CELLS=COUNT COLUMN /COUNT ROUND CELL /METHOD=EXACT TIMER(5).
Resources	Processor Time	00:00:00,01
	Elapsed Time	00:00:00,00
	Dimensions Requested	2
	Cells Available	524245
	Time for Exact Statistics	0:00:00,00

Case Processing Summary

	Cases					
	Valid		Missing		Total	
	N	Percent	N	Percent	N	Percent
syndrome / genetic disorder * hepatopathy associated with cardiac procedere	50	100,0%	0	0,0%	50	100,0%

syndrome / genetic disorder * hepatopathy associated with cardiac procedere Crosstabulation

			hepatopathy associated with
			no
syndrome / genetic disorder	not present	Count	26
		% within hepatopathy associated with cardiac procedere	86,7%
	trisomy 21	Count	2
		% within hepatopathy associated with cardiac procedere	6,7%
	DiGeorge syndrome	Count	1
		% within hepatopathy associated with cardiac procedere	3,3%
	CHARGE associtaion	Count	0
		% within hepatopathy associated with cardiac procedere	0,0%
	VACTERL associtaion	Count	1
		% within hepatopathy associated with cardiac procedere	3,3%
	others	Count	0
		% within hepatopathy associated with cardiac procedere	0,0%
Total		Count	30
		% within hepatopathy associated with cardiac procedere	100,0%

syndrome / genetic disorder * hepatopathy associated with cardiac procedere Crosstabulation

			hepatopathy associated with...
			yes
syndrome / genetic disorder	not present	Count	15
		% within hepatopathy associated with cardiac procedere	75,0%
	trisomy 21	Count	1
		% within hepatopathy associated with cardiac procedere	5,0%
	DiGeorge syndrome	Count	1
		% within hepatopathy associated with cardiac procedere	5,0%
	CHARGE associtaion	Count	1
		% within hepatopathy associated with cardiac procedere	5,0%
	VACTERL associtaion	Count	1
		% within hepatopathy associated with cardiac procedere	5,0%
	others	Count	1
		% within hepatopathy associated with cardiac procedere	5,0%
Total		Count	20
		% within hepatopathy associated with cardiac procedere	100,0%

syndrome / genetic disorder * hepatopathy associated with cardiac procedere Crosstabulation

			Total
syndrome / genetic disorder	not present	Count	41
		% within hepatopathy associated with cardiac procedere	82,0%
	trisomy 21	Count	3
		% within hepatopathy associated with cardiac procedere	6,0%
	DiGeorge syndrome	Count	2
		% within hepatopathy associated with cardiac procedere	4,0%
	CHARGE associtaion	Count	1
		% within hepatopathy associated with cardiac procedere	2,0%
	VACTERL associtaion	Count	2
		% within hepatopathy associated with cardiac procedere	4,0%
	others	Count	1
		% within hepatopathy associated with cardiac procedere	2,0%
Total		Count	50
		% within hepatopathy associated with cardiac procedere	100,0%

Chi-Square Tests

	Value	df	Asymptotic Significance (2-sided)	Exact Sig. (2-sided)	Exact Sig. (1-sided)
Pearson Chi-Square	3,421[a]	5	,635	,831	
Likelihood Ratio	4,087	5	,537	,883	
Fisher's Exact Test	3,911			,698	
Linear-by-Linear Association	2,068[b]	1	,150	,181	,102
N of Valid Cases	50				

Chi-Square Tests

	Point Probability
Pearson Chi-Square	
Likelihood Ratio	
Fisher's Exact Test	
Linear-by-Linear Association	,037
N of Valid Cases	

a. 10 cells (83,3%) have expected count less than 5. The minimum expected count is ,40.

b. The standardized statistic is 1,438.

```
CROSSTABS
  /TABLES=unsched_reop BY hepatopathy
  /FORMAT=AVALUE TABLES
  /STATISTICS=CHISQ
  /CELLS=COUNT COLUMN
  /COUNT ROUND CELL
  /METHOD=EXACT TIMER(5).
```

Crosstabs

Notes

Output Created		13-FEB-2021 14:16:37
Comments		
Input	Active Dataset	procedere_associtaed_hepatopathy
	Filter	<none>
	Weight	<none>
	Split File	<none>
	N of Rows in Working Data File	50
Missing Value Handling	Definition of Missing	User-defined missing values are treated as missing.
	Cases Used	Statistics for each table are based on all the cases with valid data in the specified range(s) for all variables in each table.
Syntax		CROSSTABS<br><br>/TABLES=unsched_reop BY hepatopathy<br>/FORMAT=AVALUE TABLES<br>/STATISTICS=CHISQ<br>/CELLS=COUNT COLUMN<br>/COUNT ROUND CELL<br>/METHOD=EXACT TIMER(5).
Resources	Processor Time	00:00:00,01
	Elapsed Time	00:00:00,00
	Dimensions Requested	2
	Cells Available	524245
	Time for Exact Statistics	0:00:00,00

Case Processing Summary

	Cases					
	Valid		Missing		Total	
	N	Percent	N	Percent	N	Percent
unscheduled reintervention (re-operation / re-cath)? * hepatopathy associated with cardiac procedere	50	100,0%	0	0,0%	50	100,0%

unscheduled reintervention (re-operation / re-cath)? * hepatopathy associated with cardiac procedere Crosstabulation

			hepatopathy associated with cardiac procedere	
			no	yes
unscheduled reintervention (re-operation / re-cath)?	no	Count	26	9
		% within hepatopathy associated with cardiac procedere	86,7%	45,0%
	yes	Count	4	11
		% within hepatopathy associated with cardiac procedere	13,3%	55,0%
Total		Count	30	20
		% within hepatopathy associated with cardiac procedere	100,0%	100,0%

unscheduled reintervention (re-operation / re-cath)? * hepatopathy associated with cardiac procedere Crosstabulation

			Total
unscheduled reintervention (re-operation / re-cath)?	no	Count	35
		% within hepatopathy associated with cardiac procedere	70,0%
	yes	Count	15
		% within hepatopathy associated with cardiac procedere	30,0%
Total		Count	50
		% within hepatopathy associated with cardiac procedere	100,0%

Chi-Square Tests

	Value	df	Asymptotic Significance (2-sided)	Exact Sig. (2-sided)	Exact Sig. (1-sided)
Pearson Chi-Square	9,921[a]	1	,002	,004	,002
Continuity Correction[b]	8,036	1	,005		
Likelihood Ratio	10,000	1	,002	,004	,002
Fisher's Exact Test				,004	,002
Linear-by-Linear Association	9,722[c]	1	,002	,004	,002
N of Valid Cases	50				

Chi-Square Tests

	Point Probability
Pearson Chi-Square	
Continuity Correction[b]	
Likelihood Ratio	
Fisher's Exact Test	
Linear-by-Linear Association	,002
N of Valid Cases	

a. 0 cells (0,0%) have expected count less than 5. The minimum expected count is 6,00.

b. Computed only for a 2x2 table

c. The standardized statistic is 3,118.

```
CROSSTABS
 /TABLES=ecmo BY hepatopathy
 /FORMAT=AVALUE TABLES
 /STATISTICS=CHISQ
 /CELLS=COUNT COLUMN
 /COUNT ROUND CELL
 /METHOD=EXACT TIMER(5).
```

Crosstabs

Notes

Output Created		13-FEB-2021 14:16:37
Comments		
Input	Active Dataset	procedere_associtaed_hepatopathy
	Filter	<none>
	Weight	<none>
	Split File	<none>
	N of Rows in Working Data File	50
Missing Value Handling	Definition of Missing	User-defined missing values are treated as missing.
	Cases Used	Statistics for each table are based on all the cases with valid data in the specified range(s) for all variables in each table.
Syntax		CROSSTABS<br>/TABLES=ecmo BY hepatopathy<br>/FORMAT=AVALUE TABLES<br>/STATISTICS=CHISQ<br>/CELLS=COUNT COLUMN<br>/COUNT ROUND CELL<br>/METHOD=EXACT TIMER(5).
Resources	Processor Time	00:00:00,01
	Elapsed Time	00:00:00,00
	Dimensions Requested	2
	Cells Available	524245
	Time for Exact Statistics	0:00:00,00

Case Processing Summary

	Cases					
	Valid		Missing		Total	
	N	Percent	N	Percent	N	Percent
ECMO after operation? * hepatopathy associated with cardiac procedere	50	100,0%	0	0,0%	50	100,0%

ECMO after operation? * hepatopathy associated with cardiac procedere Crosstabulation

			hepatopathy associated with cardiac procedere	
			no	yes
ECMO after operation?	no	Count	26	13
		% within hepatopathy associated with cardiac procedere	86,7%	65,0%
	yes	Count	4	7
		% within hepatopathy associated with cardiac procedere	13,3%	35,0%
Total		Count	30	20
		% within hepatopathy associated with cardiac procedere	100,0%	100,0%

ECMO after operation? * hepatopathy associated with cardiac procedere Crosstabulation

			Total
ECMO after operation?	no	Count	39
		% within hepatopathy associated with cardiac procedere	78,0%
	yes	Count	11
		% within hepatopathy associated with cardiac procedere	22,0%
Total		Count	50
		% within hepatopathy associated with cardiac procedere	100,0%

Chi-Square Tests

	Value	df	Asymptotic Significance (2-sided)	Exact Sig. (2-sided)	Exact Sig. (1-sided)
Pearson Chi-Square	3,283[a]	1	,070	,090	,073
Continuity Correction[b]	2,142	1	,143		
Likelihood Ratio	3,232	1	,072	,090	,073
Fisher's Exact Test				,090	,073
Linear-by-Linear Association	3,217[c]	1	,073	,090	,073
N of Valid Cases	50				

Chi-Square Tests

	Point Probability
Pearson Chi-Square	
Continuity Correction[b]	
Likelihood Ratio	
Fisher's Exact Test	
Linear-by-Linear Association	,057
N of Valid Cases	

a. 1 cells (25,0%) have expected count less than 5. The minimum expected count is 4,40.

b. Computed only for a 2x2 table

c. The standardized statistic is 1,794.

```
CROSSTABS
  /TABLES=dialy BY hepatopathy
  /FORMAT=AVALUE TABLES
  /STATISTICS=CHISQ
  /CELLS=COUNT COLUMN
  /COUNT ROUND CELL
  /METHOD=EXACT TIMER(5).
```

Crosstabs

Notes

Output Created		13-FEB-2021 14:16:37
Comments		
Input	Active Dataset	procedere_associtaed_hepatopathy
	Filter	<none>
	Weight	<none>
	Split File	<none>
	N of Rows in Working Data File	50
Missing Value Handling	Definition of Missing	User-defined missing values are treated as missing.
	Cases Used	Statistics for each table are based on all the cases with valid data in the specified range(s) for all variables in each table.
Syntax		CROSSTABS /TABLES=dialy BY hepatopathy /FORMAT=AVALUE TABLES /STATISTICS=CHISQ /CELLS=COUNT COLUMN /COUNT ROUND CELL /METHOD=EXACT TIMER(5).
Resources	Processor Time	00:00:00,01
	Elapsed Time	00:00:00,00
	Dimensions Requested	2
	Cells Available	524245
	Time for Exact Statistics	0:00:00,00

Case Processing Summary

	Cases					
	Valid		Missing		Total	
	N	Percent	N	Percent	N	Percent
dialysis after operation? * hepatopathy associated with cardiac procedere	50	100,0%	0	0,0%	50	100,0%

dialysis after operation? * hepatopathy associated with cardiac procedere Crosstabulation

			hepatopathy associated with cardiac procedere	
			no	yes
dialysis after operation?	no	Count	27	16
		% within hepatopathy associated with cardiac procedere	90,0%	80,0%
	yes	Count	3	4
		% within hepatopathy associated with cardiac procedere	10,0%	20,0%
Total		Count	30	20
		% within hepatopathy associated with cardiac procedere	100,0%	100,0%

dialysis after operation? * hepatopathy associated with cardiac procedere Crosstabulation

			Total
dialysis after operation?	no	Count	43
		% within hepatopathy associated with cardiac procedere	86,0%
	yes	Count	7
		% within hepatopathy associated with cardiac procedere	14,0%
Total		Count	50
		% within hepatopathy associated with cardiac procedere	100,0%

Chi-Square Tests

	Value	df	Asymptotic Significance (2-sided)	Exact Sig. (2-sided)	Exact Sig. (1-sided)
Pearson Chi-Square	,997[a]	1	,318	,416	,277
Continuity Correction[b]	,339	1	,560		
Likelihood Ratio	,975	1	,323	,416	,277
Fisher's Exact Test				,416	,277
Linear-by-Linear Association	,977[c]	1	,323	,416	,277
N of Valid Cases	50				

Chi-Square Tests

	Point Probability
Pearson Chi-Square	
Continuity Correction[b]	
Likelihood Ratio	
Fisher's Exact Test	
Linear-by-Linear Association	,197
N of Valid Cases	

a. 2 cells (50,0%) have expected count less than 5. The minimum expected count is 2,80.

b. Computed only for a 2x2 table

c. The standardized statistic is ,988.

```
CROSSTABS
 /TABLES=rh_dysfct BY hepatopathy
 /FORMAT=AVALUE TABLES
 /STATISTICS=CHISQ
 /CELLS=COUNT COLUMN
 /COUNT ROUND CELL
 /METHOD=EXACT TIMER(5).
```

Crosstabs

Notes

Output Created		13-FEB-2021 14:16:37
Comments		
Input	Active Dataset	procedere_associtaed_hepatopathy
	Filter	<none>
	Weight	<none>
	Split File	<none>
	N of Rows in Working Data File	50
Missing Value Handling	Definition of Missing	User-defined missing values are treated as missing.
	Cases Used	Statistics for each table are based on all the cases with valid data in the specified range(s) for all variables in each table.
Syntax		CROSSTABS<br>/TABLES=rh_dysfct BY hepatopathy<br>/FORMAT=AVALUE TABLES<br>/STATISTICS=CHISQ<br>/CELLS=COUNT COLUMN<br>/COUNT ROUND CELL<br>/METHOD=EXACT TIMER(5).
Resources	Processor Time	00:00:00,01
	Elapsed Time	00:00:00,00
	Dimensions Requested	2
	Cells Available	524245
	Time for Exact Statistics	0:00:00,01

Case Processing Summary

	Cases					
	Valid		Missing		Total	
	N	Percent	N	Percent	N	Percent
right heart dysfunction after operation? * hepatopathy associated with cardiac procedere	50	100,0%	0	0,0%	50	100,0%

right heart dysfunction after operation? * hepatopathy associated with cardiac procedere Crosstabulation

			hepatopathy associated with
			no
right heart dysfunction after operation?	no right heart dysfunction	Count	6
		% within hepatopathy associated with cardiac procedere	20,0%
	mild right heart dysfunction	Count	17
		% within hepatopathy associated with cardiac procedere	56,7%
	moderate right heart dysfunction	Count	6
		% within hepatopathy associated with cardiac procedere	20,0%
	serve right heart dysfunction	Count	1
		% within hepatopathy associated with cardiac procedere	3,3%
Total		Count	30
		% within hepatopathy associated with cardiac procedere	100,0%

right heart dysfunction after operation? * hepatopathy associated with cardiac procedere Crosstabulation

			hepatopathy associated with...
			yes
right heart dysfunction after operation?	no right heart dysfunction	Count	0
		% within hepatopathy associated with cardiac procedere	0,0%
	mild right heart dysfunction	Count	5
		% within hepatopathy associated with cardiac procedere	25,0%
	moderate right heart dysfunction	Count	7
		% within hepatopathy associated with cardiac procedere	35,0%
	serve right heart dysfunction	Count	8
		% within hepatopathy associated with cardiac procedere	40,0%
Total		Count	20
		% within hepatopathy associated with cardiac procedere	100,0%

right heart dysfunction after operation? * hepatopathy associated with cardiac procedere Crosstabulation

			Total
right heart dysfunction after operation?	no right heart dysfunction	Count	6
		% within hepatopathy associated with cardiac procedere	12,0%
	mild right heart dysfunction	Count	22
		% within hepatopathy associated with cardiac procedere	44,0%
	moderate right heart dysfunction	Count	13
		% within hepatopathy associated with cardiac procedere	26,0%
	serve right heart dysfunction	Count	9
		% within hepatopathy associated with cardiac procedere	18,0%
Total		Count	50
		% within hepatopathy associated with cardiac procedere	100,0%

Chi-Square Tests

	Value	df	Asymptotic Significance (2-sided)	Exact Sig. (2-sided)	Exact Sig. (1-sided)
Pearson Chi-Square	16,736[a]	3	,001	,000	
Likelihood Ratio	19,495	3	,000	,000	
Fisher's Exact Test	16,355			,000	
Linear-by-Linear Association	16,237[b]	1	,000	,000	,000
N of Valid Cases	50				

Chi-Square Tests

	Point Probability
Pearson Chi-Square	
Likelihood Ratio	
Fisher's Exact Test	
Linear-by-Linear Association	,000
N of Valid Cases	

a. 3 cells (37,5%) have expected count less than 5. The minimum expected count is 2,40.

b. The standardized statistic is 4,030.

```
MEANS TABLES=  rh_dysfct BY hepatopathy
   hepatopathy
 /CELLS=MEAN MEDIAN MIN MAX STDDEV.
```

Means

Notes

Output Created		13-FEB-2021 14:16:37
Comments		
Input	Active Dataset	procedere_associtaed_hepatopathy
	Filter	<none>
	Weight	<none>
	Split File	<none>
	N of Rows in Working Data File	50
Missing Value Handling	Definition of Missing	For each dependent variable in a table, user-defined missing values for the dependent and all grouping variables are treated as missing.
	Cases Used	Cases used for each table have no missing values in any independent variable, and not all dependent variables have missing values.
Syntax		MEANS TABLES= rh_dysfct BY hepatopathy hepatopathy /CELLS=MEAN MEDIAN MIN MAX ...
Resources	Processor Time	00:00:00,00
	Elapsed Time	00:00:00,00

Case Processing Summary

	Cases					
	Included		Excluded		Total	
	N	Percent	N	Percent	N	Percent
right heart dysfunction after operation? * hepatopathy associated with cardiac procedere	50	100,0%	0	0,0%	50	100,0%

right heart dysfunction after operation? * hepatopathy associated with cardiac procedere

right heart dysfunction after operation?

hepatopathy associated with cardiac procedere	Mean	Median	Minimum	Maximum	Std. Deviation
no	1,07	1,00	no right heart dysfunction	serve right heart dysfunction	,740
yes	2,15	2,00	mild right heart dysfunction	serve right heart dysfunction	,813
Total	1,50	1,00	no right heart dysfunction	serve right heart dysfunction	,931

```
CROSSTABS
  /TABLES=lh_dysfct BY hepatopathy
  /FORMAT=AVALUE TABLES
  /STATISTICS=CHISQ
  /CELLS=COUNT COLUMN
  /COUNT ROUND CELL
  /METHOD=EXACT TIMER(5).
```

Crosstabs

Notes

Output Created		13-FEB-2021 14:16:37
Comments		
Input	Active Dataset	procedere_associtaed_hepatopathy
	Filter	<none>
	Weight	<none>
	Split File	<none>
	N of Rows in Working Data File	50
Missing Value Handling	Definition of Missing	User-defined missing values are treated as missing.
	Cases Used	Statistics for each table are based on all the cases with valid data in the specified range(s) for all variables in each table.
Syntax		CROSSTABS /TABLES=lh_dysfct BY hepatopathy /FORMAT=AVALUE TABLES /STATISTICS=CHISQ /CELLS=COUNT COLUMN /COUNT ROUND CELL /METHOD=EXACT TIMER(5).
Resources	Processor Time	00:00:00,01
	Elapsed Time	00:00:00,00
	Dimensions Requested	2
	Cells Available	524245
	Time for Exact Statistics	0:00:00,01

Case Processing Summary

	Cases					
	Valid		Missing		Total	
	N	Percent	N	Percent	N	Percent
left heart dysfunction after operation? * hepatopathy associated with cardiac procedere	50	100,0%	0	0,0%	50	100,0%

left heart dysfunction after operation? * hepatopathy associated with cardiac procedere Crosstabulation

			hepatopathy associated with
			no
left heart dysfunction after operation?	no left heart dysfunction	Count	6
		% within hepatopathy associated with cardiac procedere	20,0%
	mild left heart dysfunction	Count	20
		% within hepatopathy associated with cardiac procedere	66,7%
	moderate left heart dysfunction	Count	0
		% within hepatopathy associated with cardiac procedere	0,0%
	serve left heart dysfunction	Count	4
		% within hepatopathy associated with cardiac procedere	13,3%
Total		Count	30
		% within hepatopathy associated with cardiac procedere	100,0%

left heart dysfunction after operation? * hepatopathy associated with cardiac procedere Crosstabulation

			hepatopathy associated with..
			yes
left heart dysfunction after operation?	no left heart dysfunction	Count	0
		% within hepatopathy associated with cardiac procedere	0,0%
	mild left heart dysfunction	Count	7
		% within hepatopathy associated with cardiac procedere	35,0%
	moderate left heart dysfunction	Count	10
		% within hepatopathy associated with cardiac procedere	50,0%
	serve left heart dysfunction	Count	3
		% within hepatopathy associated with cardiac procedere	15,0%
Total		Count	20
		% within hepatopathy associated with cardiac procedere	100,0%

left heart dysfunction after operation? * hepatopathy associated with cardiac procedere Crosstabulation

			Total
left heart dysfunction after operation?	no left heart dysfunction	Count	6
		% within hepatopathy associated with cardiac procedere	12,0%
	mild left heart dysfunction	Count	27
		% within hepatopathy associated with cardiac procedere	54,0%
	moderate left heart dysfunction	Count	10
		% within hepatopathy associated with cardiac procedere	20,0%
	serve left heart dysfunction	Count	7
		% within hepatopathy associated with cardiac procedere	14,0%
Total		Count	50
		% within hepatopathy associated with cardiac procedere	100,0%

Chi-Square Tests

	Value	df	Asymptotic Significance (2-sided)	Exact Sig. (2-sided)	Exact Sig. (1-sided)
Pearson Chi-Square	21,252[a]	3	,000	,000	
Likelihood Ratio	26,837	3	,000	,000	
Fisher's Exact Test	21,807			,000	
Linear-by-Linear Association	8,428[b]	1	,004	,004	,003
N of Valid Cases	50				

Chi-Square Tests

	Point Probability
Pearson Chi-Square	
Likelihood Ratio	
Fisher's Exact Test	
Linear-by-Linear Association	,002
N of Valid Cases	

a. 5 cells (62,5%) have expected count less than 5. The minimum expected count is 2,40.

b. The standardized statistic is 2,903.

```
MEANS TABLES= lh_dysfct BY hepatopathy
   hepatopathy
 /CELLS=MEAN MEDIAN MIN MAX STDDEV.
```

Means

Notes

Output Created		13-FEB-2021 14:16:37
Comments		
Input	Active Dataset	procedere_associtaed_hepatopathy
	Filter	<none>
	Weight	<none>
	Split File	<none>
	N of Rows in Working Data File	50
Missing Value Handling	Definition of Missing	For each dependent variable in a table, user-defined missing values for the dependent and all grouping variables are treated as missing.
	Cases Used	Cases used for each table have no missing values in any independent variable, and not all dependent variables have missing values.
Syntax		MEANS TABLES= lh_dysfct BY hepatopathy hepatopathy /CELLS=MEAN MEDIAN MIN MAX ...
Resources	Processor Time	00:00:00,00
	Elapsed Time	00:00:00,00

Case Processing Summary

	Cases					
	Included		Excluded		Total	
	N	Percent	N	Percent	N	Percent
left heart dysfunction after operation? * hepatopathy associated with cardiac procedere	50	100,0%	0	0,0%	50	100,0%

left heart dysfunction after operation? * hepatopathy associated with cardiac procedere

left heart dysfunction after operation?

hepatopathy associated with cardiac procedere	Mean	Median	Minimum	Maximum	Std. Deviation
no	1,07	1,00	no left heart dysfunction	serve left heart dysfunction	,868
yes	1,80	2,00	mild left heart dysfunction	serve left heart dysfunction	,696
Total	1,36	1,00	no left heart dysfunction	serve left heart dysfunction	,875

```
CROSSTABS
  /TABLES= hypotens BY hepatopathy
  /FORMAT=AVALUE TABLES
  /STATISTICS=CHISQ
  /CELLS=COUNT COLUMN
  /COUNT ROUND CELL
  /METHOD=EXACT TIMER(5).
```

Crosstabs

Notes

Output Created		13-FEB-2021 14:16:37
Comments		
Input	Active Dataset	procedere_associtaed_hepatopathy
	Filter	<none>
	Weight	<none>
	Split File	<none>
	N of Rows in Working Data File	50
Missing Value Handling	Definition of Missing	User-defined missing values are treated as missing.
	Cases Used	Statistics for each table are based on all the cases with valid data in the specified range(s) for all variables in each table.
Syntax		CROSSTABS<br>/TABLES= hypotens BY hepatopathy<br>/FORMAT=AVALUE TABLES<br>/STATISTICS=CHISQ<br>/CELLS=COUNT COLUMN<br>/COUNT ROUND CELL<br>/METHOD=EXACT TIMER(5).
Resources	Processor Time	00:00:00,01
	Elapsed Time	00:00:00,00
	Dimensions Requested	2
	Cells Available	524245
	Time for Exact Statistics	0:00:00,00

Case Processing Summary

	Cases					
	Valid		Missing		Total	
	N	Percent	N	Percent	N	Percent
Hypotension requiring therapy after operation? * hepatopathy associated with cardiac procedere	50	100,0%	0	0,0%	50	100,0%

Hypotension requiring therapy after operation? * hepatopathy associated with cardiac procedere Crosstabulation

			hepatopathy associated with
			no
Hypotension requiring therapy after operation?	no hypotension	Count	7
		% within hepatopathy associated with cardiac procedere	23,3%
	mild hypotension	Count	19
		% within hepatopathy associated with cardiac procedere	63,3%
	moderate hypotension	Count	4
		% within hepatopathy associated with cardiac procedere	13,3%
	serve hypotension	Count	0
		% within hepatopathy associated with cardiac procedere	0,0%
Total		Count	30
		% within hepatopathy associated with cardiac procedere	100,0%

Hypotension requiring therapy after operation? * hepatopathy associated with cardiac procedere Crosstabulation

			hepatopathy associated with...
			yes
Hypotension requiring therapy after operation?	no hypotension	Count	0
		% within hepatopathy associated with cardiac procedere	0,0%
	mild hypotension	Count	6
		% within hepatopathy associated with cardiac procedere	30,0%
	moderate hypotension	Count	11
		% within hepatopathy associated with cardiac procedere	55,0%
	serve hypotension	Count	3
		% within hepatopathy associated with cardiac procedere	15,0%
Total		Count	20
		% within hepatopathy associated with cardiac procedere	100,0%

Hypotension requiring therapy after operation? * hepatopathy associated with cardiac procedere Crosstabulation

			Total
Hypotension requiring therapy after operation?	no hypotension	Count	7
		% within hepatopathy associated with cardiac procedere	14,0%
	mild hypotension	Count	25
		% within hepatopathy associated with cardiac procedere	50,0%
	moderate hypotension	Count	15
		% within hepatopathy associated with cardiac procedere	30,0%
	serve hypotension	Count	3
		% within hepatopathy associated with cardiac procedere	6,0%
Total		Count	50
		% within hepatopathy associated with cardiac procedere	100,0%

Chi-Square Tests

	Value	df	Asymptotic Significance (2-sided)	Exact Sig. (2-sided)	Exact Sig. (1-sided)
Pearson Chi-Square	18,778[a]	3	,000	,000	
Likelihood Ratio	22,350	3	,000	,000	
Fisher's Exact Test	18,069			,000	
Linear-by-Linear Association	17,642[b]	1	,000	,000	,000
N of Valid Cases	50				

Chi-Square Tests

	Point Probability
Pearson Chi-Square	
Likelihood Ratio	
Fisher's Exact Test	
Linear-by-Linear Association	,000
N of Valid Cases	

a. 4 cells (50,0%) have expected count less than 5. The minimum expected count is 1,20.

b. The standardized statistic is 4,200.

```
MEANS TABLES=  hypotens BY hepatopathy
   hepatopathy
 /CELLS=MEAN MEDIAN MIN MAX STDDEV.
```

Means

Notes

Output Created		13-FEB-2021 14:16:37
Comments		
Input	Active Dataset	procedere_associtaed_hepatopathy
	Filter	<none>
	Weight	<none>
	Split File	<none>
	N of Rows in Working Data File	50
Missing Value Handling	Definition of Missing	For each dependent variable in a table, user-defined missing values for the dependent and all grouping variables are treated as missing.
	Cases Used	Cases used for each table have no missing values in any independent variable, and not all dependent variables have missing values.
Syntax		MEANS TABLES= hypotens BY hepatopathy hepatopathy /CELLS=MEAN MEDIAN MIN MAX ...
Resources	Processor Time	00:00:00,00
	Elapsed Time	00:00:00,00

Case Processing Summary

	Cases					
	Included		Excluded		Total	
	N	Percent	N	Percent	N	Percent
Hypotension requiring therapy after operation? * hepatopathy associated with cardiac procedere	50	100,0%	0	0,0%	50	100,0%

Hypotension requiring therapy after operation? * hepatopathy associated with cardiac procedere

Hypotension requiring therapy after operation?

hepatopathy associated with cardiac procedere	Mean	Median	Minimum	Maximum	Std. Deviation
no	,90	1,00	no hypotension	moderate hypotension	,607
yes	1,85	2,00	mild hypotension	serve hypotension	,671
Total	1,28	1,00	no hypotension	serve hypotension	,784

```
CROSSTABS
 /TABLES=proc_rel_fact BY hepatopathy
 /FORMAT=AVALUE TABLES
 /STATISTICS=CHISQ
 /CELLS=COUNT COLUMN
 /COUNT ROUND CELL
 /METHOD=EXACT TIMER(5).
```

Crosstabs

Notes

Output Created		13-FEB-2021 14:16:37
Comments		
Input	Active Dataset	procedere_associtaed_hepatopathy
	Filter	<none>
	Weight	<none>
	Split File	<none>
	N of Rows in Working Data File	50
Missing Value Handling	Definition of Missing	User-defined missing values are treated as missing.
	Cases Used	Statistics for each table are based on all the cases with valid data in the specified range(s) for all variables in each table.
Syntax		CROSSTABS<br><br>/TABLES=proc_rel_fact BY hepatopathy<br>/FORMAT=AVALUE TABLES<br>/STATISTICS=CHISQ<br>/CELLS=COUNT COLUMN<br>/COUNT ROUND CELL<br>/METHOD=EXACT TIMER(5).
Resources	Processor Time	00:00:00,01
	Elapsed Time	00:00:00,00
	Dimensions Requested	2
	Cells Available	524245
	Time for Exact Statistics	0:00:00,00

Case Processing Summary

	Cases					
	Valid		Missing		Total	
	N	Percent	N	Percent	N	Percent
procedere related factors * hepatopathy associated with cardiac procedere	50	100,0%	0	0,0%	50	100,0%

procedere related factors * hepatopathy associated with cardiac procedere Crosstabulation

			hepatopathy associated with cardiac procedere	
			no	yes
procedere related factors	not present	Count	12	0
		% within hepatopathy associated with cardiac procedere	40,0%	0,0%
	present	Count	18	20
		% within hepatopathy associated with cardiac procedere	60,0%	100,0%
Total		Count	30	20
		% within hepatopathy associated with cardiac procedere	100,0%	100,0%

procedere related factors * hepatopathy associated with cardiac procedere Crosstabulation

			Total
procedere related factors	not present	Count	12
		% within hepatopathy associated with cardiac procedere	24,0%
	present	Count	38
		% within hepatopathy associated with cardiac procedere	76,0%
Total		Count	50
		% within hepatopathy associated with cardiac procedere	100,0%

Chi-Square Tests

	Value	df	Asymptotic Significance (2-sided)	Exact Sig. (2-sided)	Exact Sig. (1-sided)
Pearson Chi-Square	10,526[a]	1	,001	,001	,001
Continuity Correction[b]	8,448	1	,004		
Likelihood Ratio	14,727	1	,000	,001	,001
Fisher's Exact Test				,001	,001
Linear-by-Linear Association	10,316[c]	1	,001	,001	,001
N of Valid Cases	50				

Chi-Square Tests

	Point Probability
Pearson Chi-Square	
Continuity Correction[b]	
Likelihood Ratio	
Fisher's Exact Test	
Linear-by-Linear Association	,001
N of Valid Cases	

a. 1 cells (25,0%) have expected count less than 5. The minimum expected count is 4,80.

b. Computed only for a 2x2 table

c. The standardized statistic is 3,212.

```
CROSSTABS
 /TABLES=infect BY hepatopathy
 /FORMAT=AVALUE TABLES
 /STATISTICS=CHISQ
 /CELLS=COUNT COLUMN
 /COUNT ROUND CELL
 /METHOD=EXACT TIMER(5).
```

Crosstabs

Notes

Output Created		13-FEB-2021 14:16:37
Comments		
Input	Active Dataset	procedere_associtaed_hepatopathy
	Filter	<none>
	Weight	<none>
	Split File	<none>
	N of Rows in Working Data File	50
Missing Value Handling	Definition of Missing	User-defined missing values are treated as missing.
	Cases Used	Statistics for each table are based on all the cases with valid data in the specified range(s) for all variables in each table.
Syntax		CROSSTABS /TABLES=infect BY hepatopathy /FORMAT=AVALUE TABLES /STATISTICS=CHISQ /CELLS=COUNT COLUMN /COUNT ROUND CELL /METHOD=EXACT TIMER(5).
Resources	Processor Time	00:00:00,01
	Elapsed Time	00:00:00,00
	Dimensions Requested	2
	Cells Available	524245
	Time for Exact Statistics	0:00:00,00

Case Processing Summary

	Cases					
	Valid		Missing		Total	
	N	Percent	N	Percent	N	Percent
infection after operation? * hepatopathy associated with cardiac procedere	50	100,0%	0	0,0%	50	100,0%

infection after operation? * hepatopathy associated with cardiac procedere Crosstabulation

			hepatopathy associated with cardiac procedere	
			no	yes
infection after operation?	no	Count	22	5
		% within hepatopathy associated with cardiac procedere	73,3%	25,0%
	yes	Count	8	15
		% within hepatopathy associated with cardiac procedere	26,7%	75,0%
Total		Count	30	20
		% within hepatopathy associated with cardiac procedere	100,0%	100,0%

infection after operation? * hepatopathy associated with cardiac procedere Crosstabulation

			Total
infection after operation?	no	Count	27
		% within hepatopathy associated with cardiac procedere	54,0%
	yes	Count	23
		% within hepatopathy associated with cardiac procedere	46,0%
Total		Count	50
		% within hepatopathy associated with cardiac procedere	100,0%

Chi-Square Tests

	Value	df	Asymptotic Significance (2-sided)	Exact Sig. (2-sided)	Exact Sig. (1-sided)
Pearson Chi-Square	11,286[a]	1	,001	,001	,001
Continuity Correction[b]	9,424	1	,002		
Likelihood Ratio	11,706	1	,001	,001	,001
Fisher's Exact Test				,001	,001
Linear-by-Linear Association	11,060[c]	1	,001	,001	,001
N of Valid Cases	50				

Chi-Square Tests

	Point Probability
Pearson Chi-Square	
Continuity Correction[b]	
Likelihood Ratio	
Fisher's Exact Test	
Linear-by-Linear Association	,001
N of Valid Cases	

a. 0 cells (0,0%) have expected count less than 5. The minimum expected count is 9,20.

b. Computed only for a 2x2 table

c. The standardized statistic is 3,326.

```
CROSSTABS
 /TABLES=sex BY hepatopathy
 /FORMAT=AVALUE TABLES
 /STATISTICS=CHISQ
 /CELLS=COUNT COLUMN
 /COUNT ROUND CELL
 /METHOD=EXACT TIMER(5).
```

Crosstabs

Notes

Output Created		13-FEB-2021 14:16:37
Comments		
Input	Active Dataset	procedere_associtaed_hepatopathy
	Filter	<none>
	Weight	<none>
	Split File	<none>
	N of Rows in Working Data File	50
Missing Value Handling	Definition of Missing	User-defined missing values are treated as missing.
	Cases Used	Statistics for each table are based on all the cases with valid data in the specified range(s) for all variables in each table.
Syntax		CROSSTABS /TABLES=sex BY hepatopathy /FORMAT=AVALUE TABLES /STATISTICS=CHISQ /CELLS=COUNT COLUMN /COUNT ROUND CELL /METHOD=EXACT TIMER(5).
Resources	Processor Time	00:00:00,01
	Elapsed Time	00:00:00,00
	Dimensions Requested	2
	Cells Available	524245
	Time for Exact Statistics	0:00:00,00

Case Processing Summary

	Cases					
	Valid		Missing		Total	
	N	Percent	N	Percent	N	Percent
sex * hepatopathy associated with cardiac procedere	50	100,0%	0	0,0%	50	100,0%

sex * hepatopathy associated with cardiac procedere Crosstabulation

			hepatopathy associated with cardiac procedere		
			no	yes	Total
sex	male	Count	23	7	30
		% within hepatopathy associated with cardiac procedere	76,7%	35,0%	60,0%
	female	Count	7	13	20
		% within hepatopathy associated with cardiac procedere	23,3%	65,0%	40,0%
Total		Count	30	20	50
		% within hepatopathy associated with cardiac procedere	100,0%	100,0%	100,0%

Chi-Square Tests

	Value	df	Asymptotic Significance (2-sided)	Exact Sig. (2-sided)	Exact Sig. (1-sided)
Pearson Chi-Square	8,681[a]	1	,003	,007	,004
Continuity Correction[b]	7,031	1	,008		
Likelihood Ratio	8,807	1	,003	,007	,004
Fisher's Exact Test				,007	,004
Linear-by-Linear Association	8,507[c]	1	,004	,007	,004
N of Valid Cases	50				

Chi-Square Tests

	Point Probability
Pearson Chi-Square	
Continuity Correction[b]	
Likelihood Ratio	
Fisher's Exact Test	
Linear-by-Linear Association	,003
N of Valid Cases	

a. 0 cells (0,0%) have expected count less than 5. The minimum expected count is 8,00.

b. Computed only for a 2x2 table

c. The standardized statistic is 2,917.

```
CROSSTABS
  /TABLES=death BY hepatopathy
  /FORMAT=AVALUE TABLES
  /STATISTICS=CHISQ
  /CELLS=COUNT COLUMN
  /COUNT ROUND CELL
  /METHOD=EXACT TIMER(5).
```

Crosstabs

Notes

Output Created		13-FEB-2021 14:16:37
Comments		
Input	Active Dataset	procedere_associtaed_hepatopathy
	Filter	<none>
	Weight	<none>
	Split File	<none>
	N of Rows in Working Data File	50
Missing Value Handling	Definition of Missing	User-defined missing values are treated as missing.
	Cases Used	Statistics for each table are based on all the cases with valid data in the specified range(s) for all variables in each table.
Syntax		CROSSTABS /TABLES=death BY hepatopathy /FORMAT=AVALUE TABLES /STATISTICS=CHISQ /CELLS=COUNT COLUMN /COUNT ROUND CELL /METHOD=EXACT TIMER(5).
Resources	Processor Time	00:00:00,01
	Elapsed Time	00:00:00,00
	Dimensions Requested	2
	Cells Available	524245
	Time for Exact Statistics	0:00:00,00

Case Processing Summary

	Cases					
	Valid		Missing		Total	
	N	Percent	N	Percent	N	Percent
death in follow up * hepatopathy associated with cardiac procedere	50	100,0%	0	0,0%	50	100,0%

death in follow up * hepatopathy associated with cardiac procedere Crosstabulation

			hepatopathy associated with cardiac procedere		Total
			no	yes	
death in follow up	no	Count	29	11	40
		% within hepatopathy associated with cardiac procedere	96,7%	55,0%	80,0%
	yes	Count	1	9	10
		% within hepatopathy associated with cardiac procedere	3,3%	45,0%	20,0%
Total		Count	30	20	50
		% within hepatopathy associated with cardiac procedere	100,0%	100,0%	100,0%

Chi-Square Tests

	Value	df	Asymptotic Significance (2-sided)	Exact Sig. (2-sided)	Exact Sig. (1-sided)
Pearson Chi-Square	13,021[a]	1	,000	,001	,001
Continuity Correction[b]	10,547	1	,001		
Likelihood Ratio	13,746	1	,000	,001	,001
Fisher's Exact Test				,001	,001
Linear-by-Linear Association	12,760[c]	1	,000	,001	,001
N of Valid Cases	50				

Chi-Square Tests

	Point Probability
Pearson Chi-Square	
Continuity Correction[b]	
Likelihood Ratio	
Fisher's Exact Test	
Linear-by-Linear Association	,000
N of Valid Cases	

a. 1 cells (25,0%) have expected count less than 5. The minimum expected count is 4,00.

b. Computed only for a 2x2 table

c. The standardized statistic is 3,572.

```
T-TEST GROUPS=hepatopathy(0 1)
  /MISSING=ANALYSIS
  /VARIABLES=time_cpb time_act time_reperf
  /CRITERIA=CI(.95).
```

T-Test

Notes

Output Created		13-FEB-2021 14:16:37
Comments		
Input	Active Dataset	procedere_associtaed_hepatopathy
	Filter	<none>
	Weight	<none>
	Split File	<none>
	N of Rows in Working Data File	50
Missing Value Handling	Definition of Missing	User defined missing values are treated as missing.
	Cases Used	Statistics for each analysis are based on the cases with no missing or out-of-range data for any variable in the analysis.
Syntax		T-TEST GROUPS=hepatopathy(0 1)<br>/MISSING=ANALYSIS<br>/VARIABLES=time_cpb time_act time_reperf<br>/CRITERIA=CI(.95).
Resources	Processor Time	00:00:00,00
	Elapsed Time	00:00:00,00

Group Statistics

	hepatopathy associated with cardiac procedere	N	Mean	Std. Deviation
cardiopulmonary bypass time (hh:mm)	no	30	3:22	0:58
	yes	20	4:50	1:54
aortic clamp time (hh:mm)	no	30	1:53	0:38
	yes	20	2:17	0:56
reperfusion time (hh:mm)	no	30	1:02	0:36
	yes	20	1:51	1:13

Group Statistics

	hepatopathy associated with cardiac procedere	Std. Error Mean
cardiopulmonary bypass time (hh:mm)	no	0:10
	yes	0:25
aortic clamp time (hh:mm)	no	0:07
	yes	0:12
reperfusion time (hh:mm)	no	0:06
	yes	0:16

Independent Samples Test

		Levene's Test for Equality of Variances		t-test for Equality ..
		F	Sig.	t
cardiopulmonary bypass time (hh:mm)	Equal variances assumed	14,741	,000	-3,601
	Equal variances not assumed			-3,197
aortic clamp time (hh: mm)	Equal variances assumed	7,681	,008	-1,732
	Equal variances not assumed			-1,607
reperfusion time (hh:mm)	Equal variances assumed	6,916	,011	-3,116
	Equal variances not assumed			-2,754

Independent Samples Test

		t-test for Equality of Means		
		df	Sig. (2-tailed)	Mean Difference
cardiopulmonary bypass time (hh:mm)	Equal variances assumed	48	,001	-1:28
	Equal variances not assumed	25,829	,004	-1:28
aortic clamp time (hh: mm)	Equal variances assumed	48	,090	-0:23
	Equal variances not assumed	30,633	,118	-0:23
reperfusion time (hh:mm)	Equal variances assumed	48	,003	-0:48
	Equal variances not assumed	25,383	,011	-0:48

Independent Samples Test

		t-test for Equality of Means	
		Std. Error Difference	95% Confidence ... Lower
cardiopulmonary bypass time (hh:mm)	Equal variances assumed	0:24	-2:17
	Equal variances not assumed	0:27	-2:25
aortic clamp time (hh: mm)	Equal variances assumed	0:13	-0:50
	Equal variances not assumed	0:14	-0:52
reperfusion time (hh:mm)	Equal variances assumed	0:15	-1:20
	Equal variances not assumed	0:17	-1:25

Independent Samples Test

		t-test for Equality of ... 95% Confidence Interval of the ... Upper
cardiopulmonary bypass time (hh:mm)	Equal variances assumed	-0:39
	Equal variances not assumed	-0:31
aortic clamp time (hh: mm)	Equal variances assumed	0:03
	Equal variances not assumed	0:06
reperfusion time (hh:mm)	Equal variances assumed	-0:17
	Equal variances not assumed	-0:12

```
T-TEST GROUPS=hepatopathy(0 1)
 /MISSING=ANALYSIS
 /VARIABLES=sts_eacts
 /CRITERIA=CI(.95).
```

T-Test

Notes

Output Created		13-FEB-2021 14:16:37
Comments		
Input	Active Dataset	procedere_associtaed_hepatopathy
	Filter	<none>
	Weight	<none>
	Split File	<none>
	N of Rows in Working Data File	50
Missing Value Handling	Definition of Missing	User defined missing values are treated as missing.
	Cases Used	Statistics for each analysis are based on the cases with no missing or out-of-range data for any variable in the analysis.
Syntax		T-TEST GROUPS=hepatopathy(0 1)<br>/MISSING=ANALYSIS<br>/VARIABLES=sts_eacts<br>/CRITERIA=CI(.95).
Resources	Processor Time	00:00:00,00
	Elapsed Time	00:00:00,00

Group Statistics

	hepatopathy associated with cardiac procedere	N	Mean	Std. Deviation
STS-EACTS score	no	30	3,13	,629
	yes	20	3,40	,940

Group Statistics

	hepatopathy associated with cardiac procedere	Std. Error Mean
STS-EACTS score	no	,115
	yes	,210

Independent Samples Test

		Levene's Test for Equality of Variances		t-test for Equality ..
		F	Sig.	t
STS-EACTS score	Equal variances assumed	8,768	,005	-1,204
	Equal variances not assumed			-1,113

Independent Samples Test

		t-test for Equality of Means		
		df	Sig. (2-tailed)	Mean Difference
STS-EACTS score	Equal variances assumed	48	,235	-,267
	Equal variances not assumed	30,255	,274	-,267

Independent Samples Test

		t-test for Equality of Means		
			95% Confidence Interval of the Difference	
		Std. Error Difference	Lower	Upper
STS-EACTS score	Equal variances assumed	,222	-,712	,179
	Equal variances not assumed	,240	-,756	,222

```
T-TEST GROUPS=hepatopathy(0 1)
 /MISSING=ANALYSIS
 /VARIABLES=ecmo
 /CRITERIA=CI(.95).
```

T-Test

Notes

Output Created		13-FEB-2021 14:16:37
Comments		
Input	Active Dataset	procedere_associtaed_hepatopathy
	Filter	<none>
	Weight	<none>
	Split File	<none>
	N of Rows in Working Data File	50
Missing Value Handling	Definition of Missing	User defined missing values are treated as missing.
	Cases Used	Statistics for each analysis are based on the cases with no missing or out-of-range data for any variable in the analysis.
Syntax		T-TEST GROUPS=hepatopathy(0 1)<br>/MISSING=ANALYSIS<br>/VARIABLES=ecmo<br>/CRITERIA=CI(.95).
Resources	Processor Time	00:00:00,00
	Elapsed Time	00:00:00,00

Group Statistics

	hepatopathy associated with cardiac procedere	N	Mean	Std. Deviation
ECMO after operation?	no	30	,13	,346
	yes	20	,35	,489

Group Statistics

	hepatopathy associated with cardiac procedere	Std. Error Mean
ECMO after operation?	no	,063
	yes	,109

Independent Samples Test

		Levene's Test for Equality of Variances		t-test for Equality ..
		F	Sig.	t
ECMO after operation?	Equal variances assumed	12,698	,001	-1,837
	Equal variances not assumed			-1,715

Independent Samples Test

		t-test for Equality of Means		
		df	Sig. (2-tailed)	Mean Difference
ECMO after operation?	Equal variances assumed	48	,072	-,217
	Equal variances not assumed	31,467	,096	-,217

Independent Samples Test

		t-test for Equality of Means		
		Std. Error Difference	95% Confidence Interval of the Difference	
			Lower	Upper
ECMO after operation?	Equal variances assumed	,118	-,454	,021
	Equal variances not assumed	,126	-,474	,041

```
T-TEST GROUPS=hepatopathy(0 1)
  /MISSING=ANALYSIS
  /VARIABLES=secTC
  /CRITERIA=CI(.95).
```

T-Test

Notes

Output Created		13-FEB-2021 14:16:37
Comments		
Input	Active Dataset	procedere_associtaed_hepatopathy
	Filter	<none>
	Weight	<none>
	Split File	<none>
	N of Rows in Working Data File	50
Missing Value Handling	Definition of Missing	User defined missing values are treated as missing.
	Cases Used	Statistics for each analysis are based on the cases with no missing or out-of-range data for any variable in the analysis.
Syntax		T-TEST GROUPS=hepatopathy(0 1)<br>/MISSING=ANALYSIS<br>/VARIABLES=secTC<br>/CRITERIA=CI(.95).
Resources	Processor Time	00:00:00,00
	Elapsed Time	00:00:00,00

Group Statistics

	hepatopathy associated with cardiac procedere	N	Mean	Std. Deviation
secondary chest closure?	no	30	,50	,509
	yes	20	,75	,444

Group Statistics

	hepatopathy associated with cardiac procedere	Std. Error Mean
secondary chest closure?	no	,093
	yes	,099

Independent Samples Test

		Levene's Test for Equality of Variances		t-test for Equality ..
		F	Sig.	t
secondary chest closure?	Equal variances assumed	9,600	,003	-1,789
	Equal variances not assumed			-1,839

Independent Samples Test

		t-test for Equality of Means		
		df	Sig. (2-tailed)	Mean Difference
secondary chest closure?	Equal variances assumed	48	,080	-,250
	Equal variances not assumed	44,464	,073	-,250

Independent Samples Test

		t-test for Equality of Means	
		Std. Error Difference	95% Confidence ... Lower
secondary chest closure?	Equal variances assumed	,140	-,531
	Equal variances not assumed	,136	-,524

Independent Samples Test

		t-test for Equality of ... 95% Confidence Interval of the ...
		Upper
secondary chest closure?	Equal variances assumed	,031
	Equal variances not assumed	,024

```
T-TEST GROUPS=hepatopathy(0 1)
 /MISSING=ANALYSIS
 /VARIABLES=hosp_time
 /CRITERIA=CI(.95).
```

T-Test

Notes

Output Created		13-FEB-2021 14:16:37
Comments		
Input	Active Dataset	procedere_associtaed_hepatopathy
	Filter	<none>
	Weight	<none>
	Split File	<none>
	N of Rows in Working Data File	50
Missing Value Handling	Definition of Missing	User defined missing values are treated as missing.
	Cases Used	Statistics for each analysis are based on the cases with no missing or out-of-range data for any variable in the analysis.
Syntax		T-TEST GROUPS=hepatopathy(0 1)<br>/MISSING=ANALYSIS<br>/VARIABLES=hosp_time<br>/CRITERIA=CI(.95).
Resources	Processor Time	00:00:00,00
	Elapsed Time	00:00:00,00

Group Statistics

	hepatopathy associated with cardiac procedere	N	Mean	Std. Deviation
duration of hospitalisation (days)	no	30	29,50	28,673
	yes	20	101,70	72,688

Group Statistics

	hepatopathy associated with cardiac procedere	Std. Error Mean
duration of hospitalisation (days)	no	5,235
	yes	16,253

Independent Samples Test

		Levene's Test for Equality of Variances		t-test for Equality ..
		F	Sig.	t
duration of hospitalisation (days)	Equal variances assumed	10,057	,003	-4,916
	Equal variances not assumed			-4,228

Independent Samples Test

		t-test for Equality of Means		
		df	Sig. (2-tailed)	Mean Difference
duration of hospitalisation (days)	Equal variances assumed	48	,000	-72,200
	Equal variances not assumed	22,984	,000	-72,200

Independent Samples Test

		t-test for Equality of Means	
		Std. Error Difference	95% Confidence ... Lower
duration of hospitalisation (days)	Equal variances assumed	14,686	-101,728
	Equal variances not assumed	17,076	-107,525

Independent Samples Test

		t-test for Equality of ... 95% Confidence Interval of the ...
		Upper
duration of hospitalisation (days)	Equal variances assumed	-42,672
	Equal variances not assumed	-36,875

```
T-TEST GROUPS=hepatopathy(0 1)
 /MISSING=ANALYSIS
 /VARIABLES= infect
 /CRITERIA=CI(.95).
```

T-Test

Notes

Output Created		13-FEB-2021 14:16:37
Comments		
Input	Active Dataset	procedere_associtaed_hepatopathy
	Filter	<none>
	Weight	<none>
	Split File	<none>
	N of Rows in Working Data File	50
Missing Value Handling	Definition of Missing	User defined missing values are treated as missing.
	Cases Used	Statistics for each analysis are based on the cases with no missing or out-of-range data for any variable in the analysis.
Syntax		T-TEST GROUPS=hepatopathy(0 1)<br>/MISSING=ANALYSIS<br>/VARIABLES= infect<br>/CRITERIA=CI(.95).
Resources	Processor Time	00:00:00,00
	Elapsed Time	00:00:00,00

Group Statistics

	hepatopathy associated with cardiac procedere	N	Mean	Std. Deviation
infection after operation?	no	30	,27	,450
	yes	20	,75	,444

Group Statistics

	hepatopathy associated with cardiac procedere	Std. Error Mean
infection after operation?	no	,082
	yes	,099

Independent Samples Test

		Levene's Test for Equality of Variances		t-test for Equality ..
		F	Sig.	t
infection after operation?	Equal variances assumed	,067	,796	-3,741
	Equal variances not assumed			-3,750

Independent Samples Test

		t-test for Equality of Means		
		df	Sig. (2-tailed)	Mean Difference
infection after operation?	Equal variances assumed	48	,000	-,483
	Equal variances not assumed	41,226	,001	-,483

Independent Samples Test

		t-test for Equality of Means	
		Std. Error Difference	95% Confidence ...
			Lower
infection after operation?	Equal variances assumed	,129	-,743
	Equal variances not assumed	,129	-,744

Independent Samples Test

		t-test for Equality of ...
		95% Confidence Interval of the ...
		Upper
infection after operation?	Equal variances assumed	-,224
	Equal variances not assumed	-,223

```
T-TEST GROUPS=hepatopathy(0 1)
 /MISSING=ANALYSIS
 /VARIABLES= dialy
 /CRITERIA=CI(.95).
```

T-Test

Notes

Output Created		13-FEB-2021 14:16:37
Comments		
Input	Active Dataset	procedere_associtaed_hepatopathy
	Filter	<none>
	Weight	<none>
	Split File	<none>
	N of Rows in Working Data File	50
Missing Value Handling	Definition of Missing	User defined missing values are treated as missing.
	Cases Used	Statistics for each analysis are based on the cases with no missing or out-of-range data for any variable in the analysis.
Syntax		T-TEST GROUPS=hepatopathy(0 1) /MISSING=ANALYSIS /VARIABLES= dialy /CRITERIA=CI(.95).
Resources	Processor Time	00:00:00,00
	Elapsed Time	00:00:00,00

Group Statistics

	hepatopathy associated with cardiac procedere	N	Mean	Std. Deviation
dialysis after operation?	no	30	,10	,305
	yes	20	,20	,410

Group Statistics

	hepatopathy associated with cardiac procedere	Std. Error Mean
dialysis after operation?	no	,056
	yes	,092

Independent Samples Test

		Levene's Test for Equality of Variances		t-test for Equality ..
		F	Sig.	t
dialysis after operation?	Equal variances assumed	3,920	,053	-,988
	Equal variances not assumed			-,932

Independent Samples Test

		t-test for Equality of Means		
		df	Sig. (2-tailed)	Mean Difference
dialysis after operation?	Equal variances assumed	48	,328	-,100
	Equal variances not assumed	32,677	,358	-,100

Independent Samples Test

		t-test for Equality of Means	
		Std. Error Difference	95% Confidence ... Lower
dialysis after operation?	Equal variances assumed	,101	-,303
	Equal variances not assumed	,107	-,318

Independent Samples Test

		t-test for Equality of ... 95% Confidence Interval of the...
		Upper
dialysis after operation?	Equal variances assumed	,103
	Equal variances not assumed	,118

```
T-TEST GROUPS=hepatopathy(0 1)
 /MISSING=ANALYSIS
 /VARIABLES=transf_ekz transf_ffp transf_tkz
 /CRITERIA=CI(.95).
```

T-Test

Notes

Output Created		13-FEB-2021 14:16:37
Comments		
Input	Active Dataset	procedere_associtaed_hepatopathy
	Filter	<none>
	Weight	<none>
	Split File	<none>
	N of Rows in Working Data File	50
Missing Value Handling	Definition of Missing	User defined missing values are treated as missing.
	Cases Used	Statistics for each analysis are based on the cases with no missing or out-of-range data for any variable in the analysis.
Syntax		T-TEST GROUPS=hepatopathy(0 1)<br>/MISSING=ANALYSIS<br><br>/VARIABLES=transf_ekz transf_ffp transf_tkz<br>/CRITERIA=CI(.95).
Resources	Processor Time	00:00:00,00
	Elapsed Time	00:00:00,00

Group Statistics

	hepatopathy associated with cardiac procedere	N	Mean	Std. Deviation
tranfusion of red blood cells (ml)	no	30	485,17	321,203
	yes	20	833,25	530,742
tranfusion of fresh frozen plasma (ml)	no	30	536,67	281,772
	yes	20	699,25	422,282
tranfusion of platelets (ml)	no	30	104,80	135,657
	yes	20	174,25	219,025

Group Statistics

	hepatopathy associated with cardiac procedere	Std. Error Mean
tranfusion of red blood cells (ml)	no	58,643
	yes	118,678
tranfusion of fresh frozen plasma (ml)	no	51,444
	yes	94,425
tranfusion of platelets (ml)	no	24,768
	yes	48,975

Independent Samples Test

		Levene's Test for Equality of Variances		t-test for Equality ..
		F	Sig.	t
tranfusion of red blood cells (ml)	Equal variances assumed	8,256	,006	-2,892
	Equal variances not assumed			-2,630
tranfusion of fresh frozen plasma (ml)	Equal variances assumed	6,639	,013	-1,636
	Equal variances not assumed			-1,512
tranfusion of platelets (ml)	Equal variances assumed	2,727	,105	-1,387
	Equal variances not assumed			-1,265

Independent Samples Test

		t-test for Equality of Means		
		df	Sig. (2-tailed)	Mean Difference
tranfusion of red blood cells (ml)	Equal variances assumed	48	,006	-348,083
	Equal variances not assumed	28,306	,014	-348,083
tranfusion of fresh frozen plasma (ml)	Equal variances assumed	48	,108	-162,583
	Equal variances not assumed	30,210	,141	-162,583
tranfusion of platelets (ml)	Equal variances assumed	48	,172	-69,450
	Equal variances not assumed	28,730	,216	-69,450

Independent Samples Test

		t-test for Equality of Means	
		Std. Error Difference	95% Confidence ... Lower
tranfusion of red blood cells (ml)	Equal variances assumed	120,359	-590,080
	Equal variances not assumed	132,376	-619,112
tranfusion of fresh frozen plasma (ml)	Equal variances assumed	99,396	-362,432
	Equal variances not assumed	107,530	-382,124
tranfusion of platelets (ml)	Equal variances assumed	50,089	-170,161
	Equal variances not assumed	54,882	-181,742

Independent Samples Test

		t-test for Equality of ... 95% Confidence Interval of the... Upper
tranfusion of red blood cells (ml)	Equal variances assumed	-106,086
	Equal variances not assumed	-77,055
tranfusion of fresh frozen plasma (ml)	Equal variances assumed	37,265
	Equal variances not assumed	56,957
tranfusion of platelets (ml)	Equal variances assumed	31,261
	Equal variances not assumed	42,842

```
T-TEST GROUPS=hepatopathy(0 1)
  /MISSING=ANALYSIS
  /VARIABLES= age_op
  /CRITERIA=CI(.95).
```

T-Test

Notes

Output Created		13-FEB-2021 14:16:37
Comments		
Input	Active Dataset	procedere_associtaed_hepatopathy
	Filter	<none>
	Weight	<none>
	Split File	<none>
	N of Rows in Working Data File	50
Missing Value Handling	Definition of Missing	User defined missing values are treated as missing.
	Cases Used	Statistics for each analysis are based on the cases with no missing or out-of-range data for any variable in the analysis.
Syntax		T-TEST GROUPS=hepatopathy(0 1) /MISSING=ANALYSIS /VARIABLES= age_op /CRITERIA=CI(.95).
Resources	Processor Time	00:00:00,00
	Elapsed Time	00:00:00,00

Group Statistics

	hepatopathy associated with cardiac procedere	N	Mean	Std. Deviation	Std. Error Mean
age at op	no	30	115,27	117,806	21,508
	yes	20	244,35	289,836	64,809

Independent Samples Test

		Levene's Test for Equality of Variances		t-test for Equality of Means	
		F	Sig.	t	df
age at op	Equal variances assumed	9,723	,003	-2,191	48
	Equal variances not assumed			-1,890	23,231

Independent Samples Test

		t-test for Equality of Means		
		Sig. (2-tailed)	Mean Difference	Std. Error Difference
age at op	Equal variances assumed	,033	-129,083	58,904
	Equal variances not assumed	,071	-129,083	68,285

Independent Samples Test

		t-test for Equality of Means	
		95% Confidence Interval of the Difference	
		Lower	Upper
age at op	Equal variances assumed	-247,519	-10,648
	Equal variances not assumed	-270,264	12,097

```
CROSSTABS
  /TABLES=liver_histo BY hepatopathy
  /FORMAT=AVALUE TABLES
  /STATISTICS=CHISQ
  /CELLS=COUNT COLUMN
  /COUNT ROUND CELL
  /METHOD=EXACT TIMER(5).
```

Crosstabs

Notes

Output Created		13-FEB-2021 14:16:37
Comments		
Input	Active Dataset	procedere_associtaed_hepatopathy
	Filter	<none>
	Weight	<none>
	Split File	<none>
	N of Rows in Working Data File	50
Missing Value Handling	Definition of Missing	User-defined missing values are treated as missing.
	Cases Used	Statistics for each table are based on all the cases with valid data in the specified range(s) for all variables in each table.
Syntax		CROSSTABS /TABLES=liver_histo BY hepatopathy /FORMAT=AVALUE TABLES /STATISTICS=CHISQ /CELLS=COUNT COLUMN /COUNT ROUND CELL /METHOD=EXACT TIMER(5).
Resources	Processor Time	00:00:00,01
	Elapsed Time	00:00:00,00
	Dimensions Requested	2
	Cells Available	524245
	Time for Exact Statistics	0:00:00,00

Case Processing Summary

	Cases					
	Valid		Missing		Total	
	N	Percent	N	Percent	N	Percent
liver biopsy after operation? * hepatopathy associated with cardiac procedere	50	100,0%	0	0,0%	50	100,0%

liver biopsy after operation? * hepatopathy associated with cardiac procedere Crosstabulation

			hepatopathy associated with
			no
liver biopsy after operation?	no biopsy	Count	30
		% within hepatopathy associated with cardiac procedere	100,0%
	biopsy executed	Count	0
		% within hepatopathy associated with cardiac procedere	0,0%
Total		Count	30
		% within hepatopathy associated with cardiac procedere	100,0%

liver biopsy after operation? * hepatopathy associated with cardiac procedere Crosstabulation

			hepatopathy associated with...	
			yes	Total
liver biopsy after operation?	no biopsy	Count	10	40
		% within hepatopathy associated with cardiac procedere	50,0%	80,0%
	biopsy executed	Count	10	10
		% within hepatopathy associated with cardiac procedere	50,0%	20,0%
Total		Count	20	50
		% within hepatopathy associated with cardiac procedere	100,0%	100,0%

Chi-Square Tests

	Value	df	Asymptotic Significance (2-sided)	Exact Sig. (2-sided)	Exact Sig. (1-sided)
Pearson Chi-Square	18,750[a]	1	,000	,000	,000
Continuity Correction[b]	15,755	1	,000		
Likelihood Ratio	22,314	1	,000	,000	,000
Fisher's Exact Test				,000	,000
Linear-by-Linear Association	18,375[c]	1	,000	,000	,000
N of Valid Cases	50				

Chi-Square Tests

	Point Probability
Pearson Chi-Square	
Continuity Correction[b]	
Likelihood Ratio	
Fisher's Exact Test	
Linear-by-Linear Association	,000
N of Valid Cases	

a. 1 cells (25,0%) have expected count less than 5. The minimum expected count is 4,00.

b. Computed only for a 2x2 table

c. The standardized statistic is 4,287.

```
CROSSTABS
 /TABLES=liv_histo_cirr BY hepatopathy
 /FORMAT=AVALUE TABLES
 /STATISTICS=CHISQ
 /CELLS=COUNT COLUMN
 /COUNT ROUND CELL
 /METHOD=EXACT TIMER(5).
```

Crosstabs

Notes

Output Created		13-FEB-2021 14:16:37
Comments		
Input	Active Dataset	procedere_associtaed_hepatopathy
	Filter	<none>
	Weight	<none>
	Split File	<none>
	N of Rows in Working Data File	50
Missing Value Handling	Definition of Missing	User-defined missing values are treated as missing.
	Cases Used	Statistics for each table are based on all the cases with valid data in the specified range(s) for all variables in each table.
Syntax		CROSSTABS /TABLES=liv_histo_cirr BY hepatopathy /FORMAT=AVALUE TABLES /STATISTICS=CHISQ /CELLS=COUNT COLUMN /COUNT ROUND CELL /METHOD=EXACT TIMER(5).
Resources	Processor Time	00:00:00,01
	Elapsed Time	00:00:00,00
	Dimensions Requested	2
	Cells Available	524245
	Time for Exact Statistics	0:00:00,00

Case Processing Summary

	Cases					
	Valid		Missing		Total	
	N	Percent	N	Percent	N	Percent
histopathologic findings: cirrhosis * hepatopathy associated with cardiac procedere	50	100,0%	0	0,0%	50	100,0%

histopathologic findings: cirrhosis * hepatopathy associated with cardiac procedere Crosstabulation

			hepatopathy associated with cardiac procedere	
			no	yes
histopathologic findings: cirrhosis	no	Count	30	10
		% within hepatopathy associated with cardiac procedere	100,0%	50,0%
	yes	Count	0	10
		% within hepatopathy associated with cardiac procedere	0,0%	50,0%
Total		Count	30	20
		% within hepatopathy associated with cardiac procedere	100,0%	100,0%

histopathologic findings: cirrhosis * hepatopathy associated with cardiac procedere Crosstabulation

			Total
histopathologic findings: cirrhosis	no	Count	40
		% within hepatopathy associated with cardiac procedere	80,0%
	yes	Count	10
		% within hepatopathy associated with cardiac procedere	20,0%
Total		Count	50
		% within hepatopathy associated with cardiac procedere	100,0%

Chi-Square Tests

	Value	df	Asymptotic Significance (2-sided)	Exact Sig. (2-sided)	Exact Sig. (1-sided)
Pearson Chi-Square	18,750[a]	1	,000	,000	,000
Continuity Correction[b]	15,755	1	,000		
Likelihood Ratio	22,314	1	,000	,000	,000
Fisher's Exact Test				,000	,000
Linear-by-Linear Association	18,375[c]	1	,000	,000	,000
N of Valid Cases	50				

Chi-Square Tests

	Point Probability
Pearson Chi-Square	
Continuity Correction[b]	
Likelihood Ratio	
Fisher's Exact Test	
Linear-by-Linear Association	,000
N of Valid Cases	

a. 1 cells (25,0%) have expected count less than 5. The minimum expected count is 4,00.

b. Computed only for a 2x2 table

c. The standardized statistic is 4,287.

```
USE ALL.
COMPUTE filter_patient_histo=(liver_histo = 1).
VARIABLE LABELS filter_patient_histo 'numb_op = 1 (FILTER)'.
VALUE LABELS filter_patient_histo 0 'Not Selected' 1 'Selected'.
FORMATS filter_patient_histo (f1.0).
FILTER BY filter_patient_histo.
EXECUTE.
```

```
FREQUENCIES liver_histo.
```

Frequencies

Notes

Output Created		13-FEB-2021 14:16:37
Comments		
Input	Active Dataset	procedere_associtaed_hepatopathy
	Filter	numb_op = 1 (FILTER)
	Weight	<none>
	Split File	<none>
	N of Rows in Working Data File	10
Missing Value Handling	Definition of Missing	User-defined missing values are treated as missing.
	Cases Used	Statistics are based on all cases with valid data.
Syntax		FREQUENCIES liver_histo.
Resources	Processor Time	00:00:00,00
	Elapsed Time	00:00:00,00

Statistics

liver biopsy after operation?

N	Valid	10
	Missing	0

liver biopsy after operation?

		Frequency	Percent	Valid Percent	Cumulative Percent
Valid	biopsy executed	10	100,0	100,0	100,0

```
FREQUENCIES liv_histo_cirr .
```

Frequencies

Notes

Output Created		13-FEB-2021 14:16:37
Comments		
Input	Active Dataset	procedere_associtaed_hepatopathy
	Filter	numb_op = 1 (FILTER)
	Weight	<none>
	Split File	<none>
	N of Rows in Working Data File	10
Missing Value Handling	Definition of Missing	User-defined missing values are treated as missing.
	Cases Used	Statistics are based on all cases with valid data.
Syntax		FREQUENCIES liv_histo_cirr .
Resources	Processor Time	00:00:00,00
	Elapsed Time	00:00:00,00

Statistics

histopathologic findings: cirrhosis

N	Valid	10
	Missing	0

histopathologic findings: cirrhosis

		Frequency	Percent	Valid Percent	Cumulative Percent
Valid	yes	10	100,0	100,0	100,0

```
USE all.
```

```
SAVE
  OUTFILE='/Users/torbenkehl/@work/Kinderkardiologie/Studien/Hepatopathie nach CPB OP/Revision/hepatopathy_cpb_anonym.sav'
  /COMPRESSED.
```
